# Intra-Tumoral Heterogeneity in Metastatic Potential and Survival Signaling between Iso-Clonal HCT116 and HCT116b Human Colon Carcinoma Cell Lines

**DOI:** 10.1371/journal.pone.0060299

**Published:** 2013-04-01

**Authors:** Sanjib Chowdhury, Melanie Ongchin, Elizabeth Sharratt, Ivan Dominguez, Jing Wang, Michael G. Brattain, Ashwani Rajput

**Affiliations:** 1 Eppley Cancer Center, University of Nebraska Medical Center, Omaha, Nebraska, United States of America; 2 Department of Surgery, State University of New York at Buffalo, Buffalo, New York, United States of America; 3 Department of Pharmacology & Therapeutics, Roswell Park Cancer Institute, Buffalo, New York, United States of America; 4 Division of Surgical Oncology, Department of Surgery, University of New Mexico Health Sciences Center, Albuquerque, New Mexico, United States of America; University of Navarra, Spain

## Abstract

**Background:**

Colorectal cancer (CRC) metastasis is a leading cause of cancer-related deaths in the United States. The molecular mechanisms underlying this complex, multi-step pathway are yet to be completely elucidated. Recent reports have stressed the importance of intra-tumoral heterogeneity in the development of a metastatic phenotype. The purpose of this study was to characterize the intra-tumoral phenotypic heterogeneity between two iso-clonal human colon cancer sublines HCT116 and HCT116b on their ability to undergo metastatic colonization and survive under growth factor deprivation stress (GFDS).

**Materials and Methods:**

HCT116 and HCT116b cells were transfected with green fluorescence protein and subcutaneously injected into BALB/c nude male mice. Once xenografts were established, they were excised and orthotopically implanted into other male BALB/c nude mice using microsurgical techniques. Animal tissues were studied for metastases using histochemical techniques. Microarray analysis was performed to generate gene signatures associated with each subline. *In vitro* assessment of growth factor signaling pathway was performed under GFDS for 3 and 5 days.

**Results:**

Both HCT116 and HCT116b iso-clonal variants demonstrated 100% primary tumor growth, invasion and peritoneal spread. However, HCT116 was highly metastatic with 68% metastasis observed in liver and/or lungs compared to 4% in HCT116b. Microarray analysis revealed an upregulation of survival and metastatic genes in HCT116 cells compared to HCT116b cells. *In vitro* analysis showed that HCT116 upregulated survival and migratory signaling proteins and downregulated apoptotic agents under GFDS. However, HCT116b cells effectively showed the opposite response under stress inducing cell death.

**Conclusions:**

We demonstrate the importance of clonal variation in determining metastatic potential of colorectal cancer cells using the HCT116/HCT116b iso-clonal variants in an orthotopic metastatic mouse model. Determination of clonal heterogeneity in patient tumors can serve as useful tools to identify clinically relevant biomarkers for diagnostic and therapeutic assessment of metastatic colorectal cancer.

## Introduction

Colorectal cancer (CRC) is a major contributor of cancer-related deaths in the United States [1]. Metastasis to distant organ sites significantly affects the mortality rate resulting from the disease [1], [2]. The Cancer Genome Atlas Network has recently reported the multi-dimensional genomic changes associated with CRC with the goal to provide deeper insight into the pathophysiology of CRC to identify potential therapeutic targets [3]. Identification and characterization of novel molecular targets for CRC metastasis is a pressing need since to date there are no effective anti-metastatic therapies available. Tumor cells display remarkable clonal heterogeneity due to both genetic and non-genetic influences [4]. Several clinically important phenotypes including the ability to undergo metastatic colonization have been attributed to this clonal variance [4]. Therefore understanding the extent of difference between these clonal variants is crucial for effectively targeting these clones. The characterization using in vivo models of the clonal heterogeneity arising from a single patient's colon tumor is still discrete.

Numerous *in vitro* and *in vivo* assays have been developed to study CRC progression. However, none of these techniques are effective in recapitulating the multi-step dissemination process. We have developed an orthotopic mouse CRC metastasis model that can quantitatively and qualitatively reproduce the metastatic phenotype of the human disease to the liver and/or lungs in an *in vivo* setting [1], [5], [6], [7], [8], [9]. Earlier work from our lab has characterized several human colon carcinoma cell lines using the orthotopic model [1], [6], [10]. In this study, we compared the iso-clonal human colon carcinoma cell lines HCT116 and HCT116b isolated from the same patient primary colon carcinoma [11]. Previously, we have demonstrated *in vitro* that HCT cells are growth factor-independent [12]. In contrast, HCT116b cells are growth factor-dependent subcompartment of the malignant HCT116 cells [12]. These isogenic cell lines demonstrate the clonal variance associated with malignant progression within tumors *in vivo* [6], [12].

Comparison of HCT116 and HCT116b cells *in vivo* showed a significant difference in their ability to form metastatic deposits. Microarray analysis comparing the primary colon carcinoma arising from the two iso-clonal variants revealed striking differences in their gene signature. *In vitro* analysis of the two iso-clonal sublines revealed differences in cell survival and motility signaling under GFDS conditions.

## Materials and Methods

### Cell Culture

HCT116 and HCT116b sublines were isolated from a primary tissue culture of a single human colon carcinoma as described by Brattain *et al*. [11]. The two cell lines were adapted to growth in serum-free medium [11], [13], [14] consisting of McCoy's 5A medium (Sigma) supplemented with amino acids, pyruvate, and antibiotics (designated SM) containing the growth factors transferrin (4 µg/ml; Sigma), insulin (20 µg/ml; Sigma) and EGF (5 ng/ml; Collaborative Research) [15].

### Green Fluorescence Protein (GFP) Transfection

Packaging cells, 293 GP (Clontech, Mountain View, CA), were co-transfected with a plasmid encoding VSVG envelope protein and a retroviral vector encoding GFP and the G418 resistance gene using FuGene (Invitrogen, Carlsbad, CA). The viruses were collected 48 h later and used to infect HCT116 and HCT116b cells. After 48 h, the infected HCT116 and HCT116b cells were selected by treatment with G418 for 5 d. This resulted in a stable transfection.

### Animals

Male athymic BALB/c nude mice between 4–6 weeks of age were acquired from the National Cancer Institute (NCI). Housing for these animals was maintained in a HEPA-filtrated environment within sterilized cages. All animals were subjected to a daily 12 hr light/12-hr dark cycle. All animal procedures were conducted with approval of and in compliance with the Roswell Park Cancer Institute Institutional Animal Care and Use Committee (IACUC).

### Orthotopic Implantation and Imaging

Exponentially growing GFP-labeled HCT116 and HCT116b cells (5×10^6^ cells) were inoculated subcutaneously onto the dorsal surfaces of separate BALB/c nude male mice. Once the xenografts were established, measuring approximately 500 mm3, they were excised and minced into 1 mm3 pieces. Two of these pieces were then orthotopically implanted into the colons of other male BALB/c nude mice as previously reported. Briefly, for operative procedures, animals were anesthetized with isoflurane inhalation. A 1 cm laparotomy was performed, and both the cecum and ascending colon were exteriorized. Using 7× magnification and microsurgical techniques, the serosa was disrupted in two different locations. The 1 mm3 pieces of xenograft were sub-serosally implanted using a 9-0 nylon suture at the two points of serosal disruption. The bowel was then returned to the peritoneal cavity and the abdomen was closed with interrupted 5-0 vicryl sutures.

### Imaging

Starting at 1 wk post-implantation, animals were anesthetized with a 1∶1 mixture of ketamine (10 mg/mL) and xylazine (1 mg/mL) by intraperitoneal injection (0.01 mL/mg) and weekly GFP fluorescence imaging was performed for up to 7 wk. Specifically, GFP fluorescence imaging was performed using a light box illuminated by fiberoptic lighting at 470 nm (Illumatool BLS; Lightools Research, Encinitas, CA) using a Retiga EXi color CCD camera (QImaging, Burnaby BC, Canada). High-resolution images were captured directly using a MS-Windows based PC. Images were visually optimized for contrast and brightness using commercial software (Adobe Photoshop, CS2; Adobe, San Jose, CA). Excitation of GFP in the light box facilitated identification of primary and metastatic disease by direct near-real time visualization of fluorescence in live animals.

### H and E Staining

At approximately 6–8 weeks post-implantation, the animals were euthanized. The colon, including any primary tumor, was harvested in addition to the liver, lungs, and heart. These organs were explanted, imaged, and immediately placed in 10% neutral buffered formalin fixative for 24 h. The tissues were then processed and embedded in paraffin. Slides were then cut for hematoxylin and eosin (H and E).

### TUNEL and Ki67 Staining

Serial sections were cut to complement the H and E sections and were stained with an IgG1 rabbit polyclonal antibody for Ki-67 (Dako North America, Inc., Carpinteria, CA). Slides from paraffin embedded tissue blocks were stained according to the Apotag (Oncor, Gaithersburg, MD) terminal nucleotidyl transferase-mediated nick end labeling (TUNEL) assay kit. The Ki67 and TUNEL assay protocols have been described in details in our previous publications [1], [6], [7], [8], [9].

### Apoptosis Assays

Apoptosis assays were performed to determine the response of HCT116 and HCT116b cells to growth factor deprivation stress (GFDS) [16]. Cells were seeded in 24-well plates and allowed to grow to 80% confluent. The cells were then changed to starving medium (SM) for 2 or 4 days. Apoptosis assays were then performed using a DNA fragmentation ELISA kit as described in the manufacturer's protocol (Invitrogen).

### Transwell Motility Assays

Transwell motility assays were performed utilizing 8- µm pore, 6.5 mm polycarbonate transwell filters (Corning Costar Corp.). After trypsinizing the cells, single cell suspensions were seeded in supplemental McCoy's 5A medium in the absence of growth factors onto the upper surface of the filters and allowed to migrate towards McCoy's 5A medium with 10% FBS. After 18 hr incubation, MTT was added to the medium. The cells on the upper surface of the filter were removed with a cotton swab, and the cells that had migrated to the underside of the filter were visualized under the microscope and dissolved in DMSO. Absorbance was read at 570 nm.

### Western Blot Analysis

Cells were lysed in a Tris-HCl based buffer containing 0.5% NP-40 and appropriate protease and phosphate inhibitors as described previously [1], [15]. Protein concentration was determined by bicinhoninic acid assay (Pierce).

### Gene Expression Analysis

The microarray dataset comparing the HCT116 and HCT116b primary colon tumors has been deposited at the Gene Expression Omnibus (GEO). The accession number to the dataset is GSE44381.

### Bioinformatics Analysis

Bioinformatics analysis was performed using the ingenuity pathway analysis (IPA) software tool obtained from the UNMC bioinformatics core facility.

## Results

### High variance in metastatic rates between HCT116 and HCT116b cells

**Figure 1:**
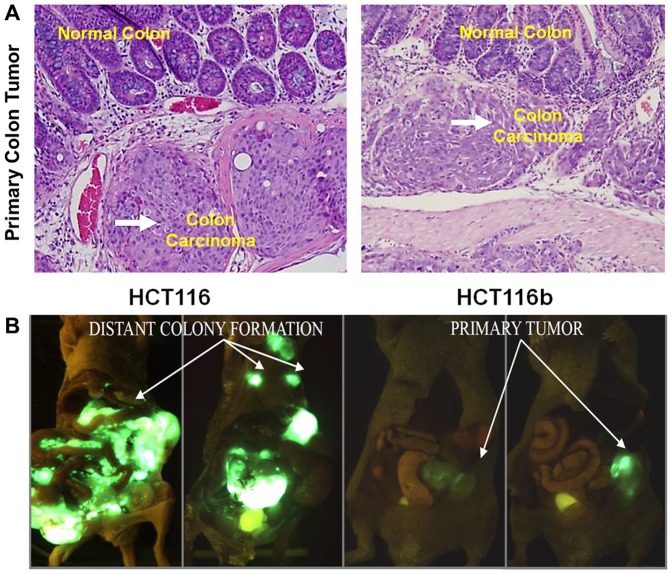
Intra-tumoral heterogeneity in the ability to develop metastatic colonization. (*A*) H&E section demonstrating normal colonic area and primary colon carcinoma (magnification 40×). (*B*) 4 to 6 week old male BALB/c nude mice were orthotopically implanted with HCT116 or HCT116b tumor xenografts. GFP imaging revealed primary tumor growth in both HCT116 and HCT116b animals. However, distant metastasis was only observed in HCT116 implanted animals by week 6.

We compared the clonal variation in metastatic capability between HCT116 cells and its iso-clonal variant HCT116b by utilizing the colonic orthotopic implantation of subcutaneously grown xenografts. The HCT116 animals were euthanized between 6–8 weeks. The HCT 116b were sacrificed on day 63 (9 weeks). The HCT116 animals had to be put down earlier because of tumor burden and animal cachexia in accordance with IACUC regulations. As shown in Table 1, both cell lines gives rise to an invasive primary tumor in 100% of the animals implanted as demonstrated by invasive sheet observed during the immunohistochemical evaluation of the HCT116 and HCT116b primary tumors (Figure 1A). Strikingly, however, the cell lines vary dramatically with respect to their metastatic potential. HCT116 implanted mice generated histologically confirmed metastasis to liver and/or lungs in 68% animals (Figure 1B). HCT116 cells colonized to the liver in 21/40 (52%) and to the lungs in 19/40 (48%) of mice. In contrast, HCT116b cells showed a liver and/or lungs metastatic rate of only about 8%. HCT116b cells colonized to the liver in 2/49 (4%) and to the lungs in 2/49 (4%) of mice (Table 1).

**Table 1:** Results of orthotopic implantation of HCT116 and HCT116b tumor xenografts demonstrating the primary invasion and metastases based on histologic evaluation.

	Local Invasion	Liver Mets	Lung Mets	Lung &/or liver Mets
HCT 116	40/40	21/40(52%)	19/40(48%)	27/40 (68%)
HCT116b	49/49	2/49 (4%)	2/49(4%)	4/49 (8%)

### Increased TUNEL staining observed in HCT116b primary tumors

**Figure 2:**
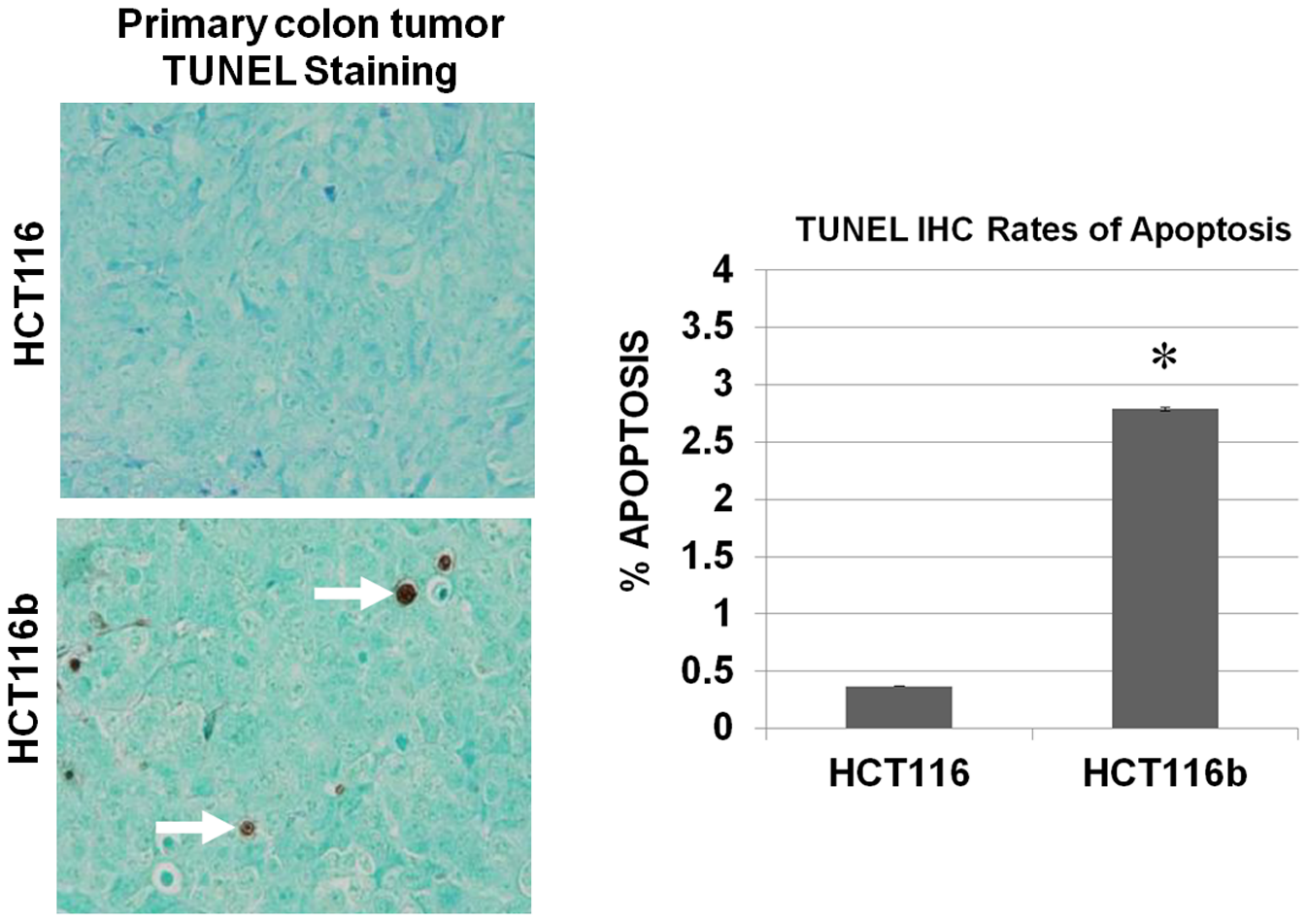
Increased cell survival associated with HCT116 cells. Comparison of primary tumor sections of HCT116 and HCT116b mice by TUNEL staining as mentioned in Materials and Methods to determine their apoptotic rates. HCT116b tumor tissues had high TUNEL staining indicative of cell death. However, HCT116 tumor tissues showed negligible response to TUNEL staining indicating high rate of cell survival.

We next characterized the HCT116 and HCT116b primary colonic tumors for their effect on cell survival and proliferation. As indicated in Figure 2, HCT116 showed an increased cell survival signaling as reflected by lower TUNEL staining rates compared to HCT116b tumors indicating that the repression of metastatic colonization in HCT116b is associated with repression of cell survival signaling *in vivo*. Interestingly, no change in Ki67 staining was observed between HCT116 and HCT116b primary tumors indicating no difference in proliferation rate *in vivo* between these clonal variants (data not shown).

### Microarray analysis profiling gene signatures associated with HCT116 and HCT116b tumors

**Figure 3:**
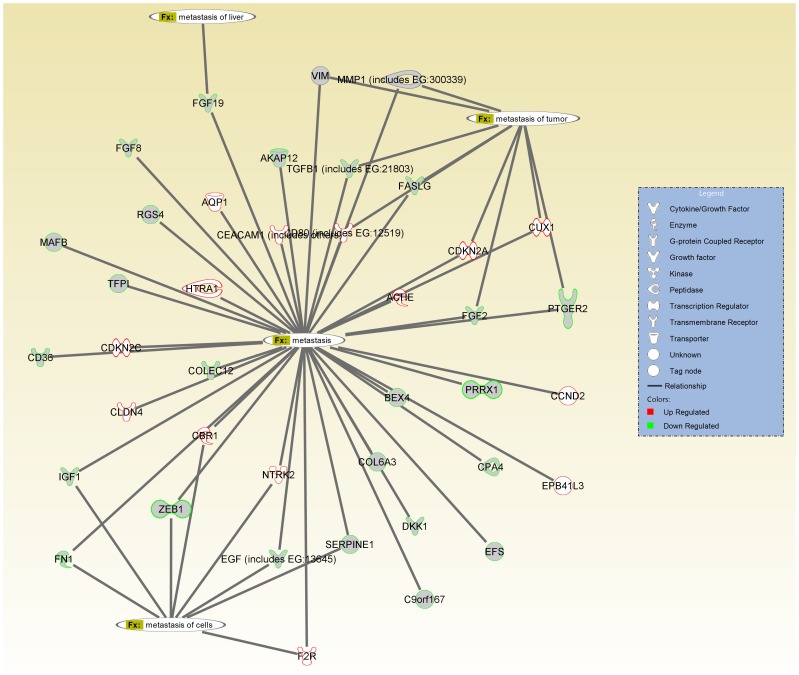
Comparison of genes signatures associated with metastasis signaling between HCT116 and HCT116b microarray datasets by Ingenuity Pathway Analysis (IPA) tool. The molecules highlighted in green are upregulated in the highly metastatic HCT116 cells and downregulated in poorly metastatic iso-clonal HCT116b cells. The molecules marked in red are upregulated in HCT116b cells compared to HCT116 cells.

Next we sought to determine the differences in gene expression between HCT116 and HCT116b primary colon carcinoma tumor samples. Transcription profiles of the samples were generated using the Affymetrix HGU133plus2.0 genechips. A heat map dendrogram generated and ranked using the 2-fold up- or down-regulation cut-off is shown in the Figure S1. A summary of genes differentially regulated between the two iso-clonal tumors have also been shown in Table S1. Gene ontology analysis was performed to delineate the differences in gene expression between different cellular compartments. As shown in Figure S2, 26% of all genes were differentially regulated in the cytoplasm. There was a 23% difference in gene expression profiles in the plasma membrane followed by 17% in the nucleus and 10% in the extracellular space. We next performed the Ingenuity Pathway Analysis (IPA) to categorize the difference in gene signatures between HCT116 and HCT116b tumors according to their molecular participation in cell survival and metastasis. Strikingly, several genes involved in providing cancer cells with the survival advantage were observed to be downregulated in HCT116b cells. Some of the genes that were differentially regulated in HCT116 and HCT116b cells have been shown in Figure 3. Among these are growth factor ligands IGF1, EGF, TGFβ and FGF that are significantly repressed in HCT116b. Similarly, genes involved in conferring metastatic ability, such as MMPs, fibronectin 1 (FN1) and vimentin (VIM) are transcriptionally repressed in HCT116b. In contrast, cyclin dependent kinase inhibitors, such as CDKN2A and CDKN2C were upregulated in HCT116b cells.

### *In vitro* analysis of cell death markers in HCT116 and HCT116b cells under GFDS

Previously, we examined the effects of nutrient and growth factor deprivation *in vitro* in HCT116 and HCT116b human colon carcinoma cells [12]. Under conditions of nutrient deprivation-mediated growth arrest, HCT116b cells required nutrients and growth factor supplementation for the reinitiation of DNA synthesis. However, HCT116 only required nutrients and showed a transcriptional upregulation of TGFα during stress [12]. Therefore the combined intrinsic ability of HCT116 cells to survive under stress and concomitantly re-enter cell cycle in response to nutrient exposure without exogenous growth factors would seem to provide the malignant cells with a substantial growth advantage in situations of limited blood supply and during the process of metastasis. Based on these prior observations and our current gene signatures obtained by microarray analysis, we hypothesized that the downstream survival pathways associated with malignant progression in CRC would be differentially regulated in HCT116 and HCT116b cell under stress conditions.

**Figure 4:**
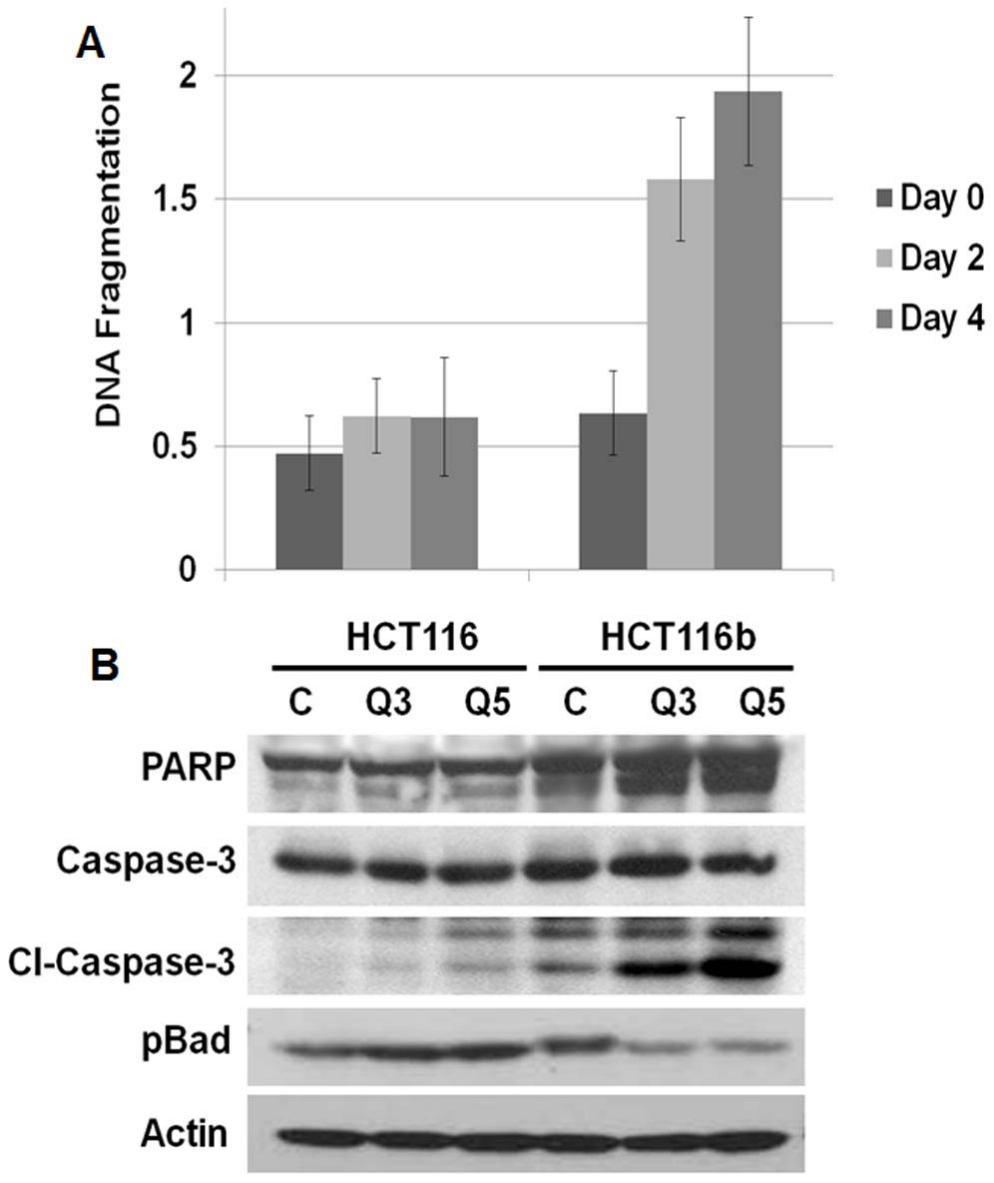
Cell death response under growth factor deprivation stress (GFDS). (*A*) HCT116 cells are resistant to GFDS-induced cell death as determined by DNA fragmentation assay. However, HCT116b iso-clonal cells induce cell death under GFDS. (*B*) HCT116b cells induce cell death by time-dependent increase in PARP and caspase 3 cleavages and dephosphorylation of anti-apoptotic pBad protein.

We first performed a DNA fragmentation assay to examine the level of cell death in these cells under nutrient and growth factor deprivation stress (GFDS) [12], [16]. Confluent cells in culture were used as control (reference point) and compared to quiescent (Q) cells that have been deprived of exogenous growth factors for 2 and 4 days respectively (termed as Q2 and Q4 respectively). HCT116 cells did not show any increase in apoptosis under GFDS for 4 days. However, HCT116b cells showed approximately 2-fold increase in cell death by Q4 (Figure 4A). We next performed western blot analysis of HCT116 and HCT116b cells under GFDS conditions to investigate the protein expression of different apoptotic markers. Interestingly, we observed a stable expression of total-PARP and -caspase-3 in HCT116 cells. In contrast, HCT116b cells showed a time-dependent increase in cleaved-PARP and -caspase-3 indicative of increased apoptosis. Similarly, we observed phosphorylation of anti-apoptotic marker pBad in HCT116 cells. However, pBad expression progressively reduced under GFDS in HCT116b cells (Figure 4B).

### In vitro analysis of cell survival signaling pathways in HCT116 and HCT116b cells under GFDS

**Figure 5:**
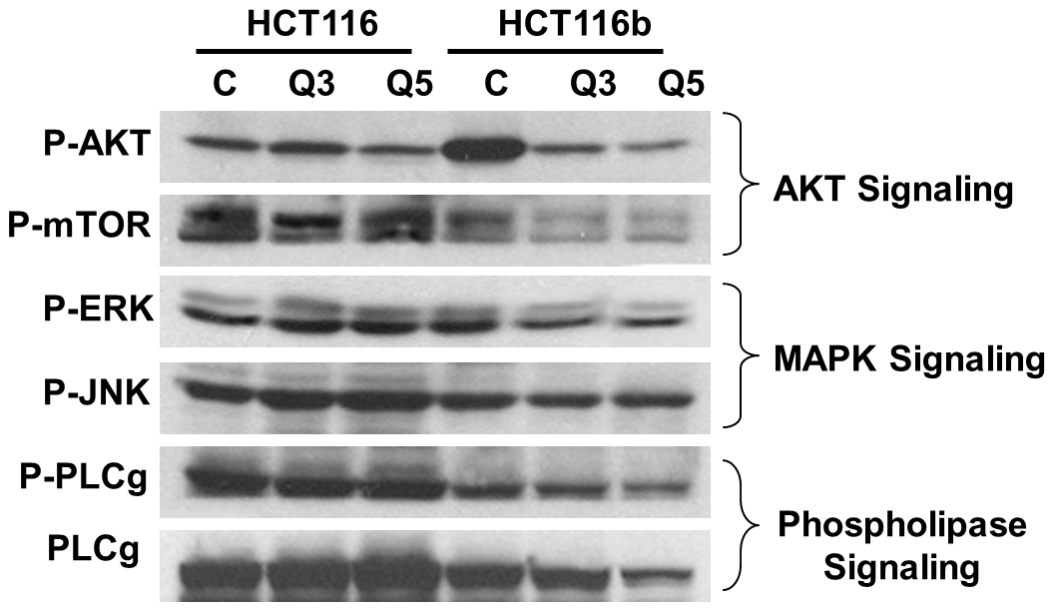
Cell survival signaling response under growth factor deprivation stress (GFDS). HCT116 cells upregulated AKT, MAPK and PLC signaling under GFDS conditions to counter stress. In contrast, HCT116b cells downregulated these signaling molecules by dephosphorylating and thereby inactivating AKT, ERK and PLC gamma to induced cell death.

We next determined the level of phosphorylation under GFDS of major signal transduction pathways such as the mitogen activated protein kinase (MAPK), phosphatidylinositol 3-kinase (PI_3_K) and phospholipase Cγ (PLCγ) pathways which control proliferation and cell survival in colon cancer [8], [16], [17], [18]. As shown in Figure 5, we observed a reduction in phosphorylation of AKT, mTOR, ERK and PLCγ proteins in HCT116b cells under GFDS. However, HCT116 cells continued to demonstrate high phosphorylation activity of these oncogenes indicative of its growth-factor independent cell survival machinery to counter stress.

### In vitro analysis of cell motility related signaling pathways in HCT116 and HCT116b cells under GFDS

**Figure 6:**
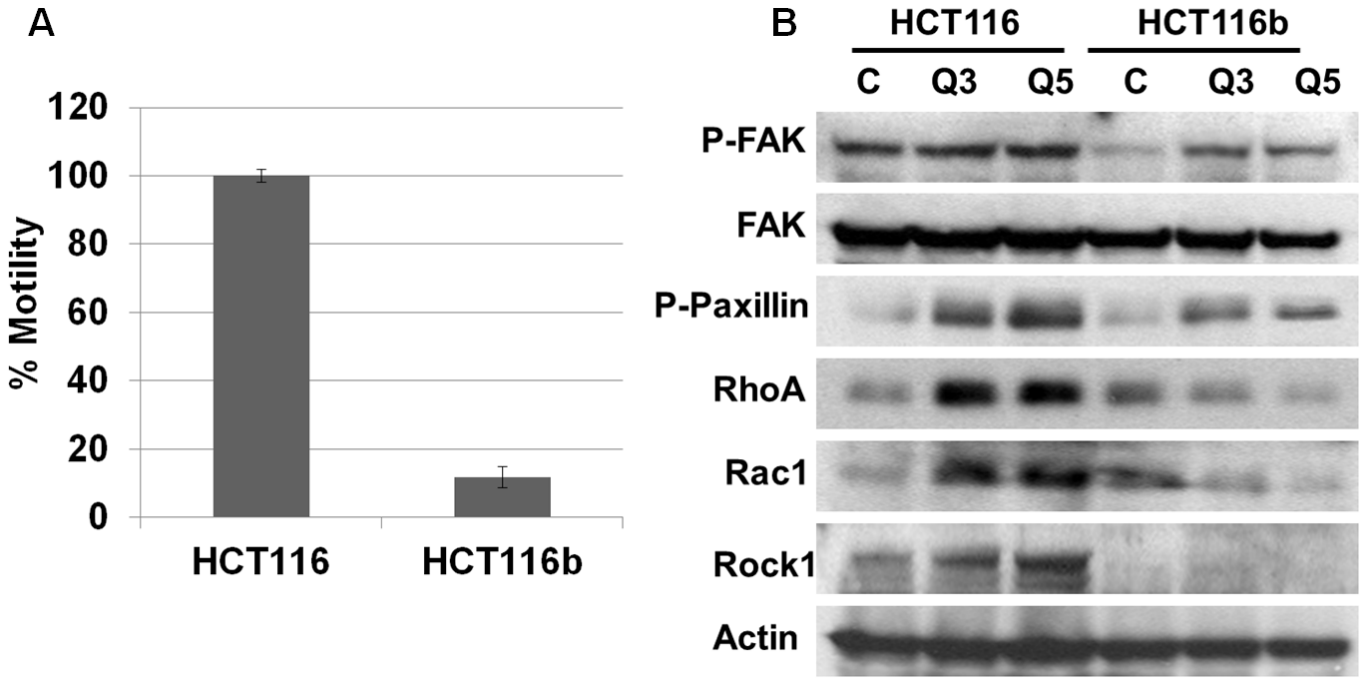
Differential cell motility responses between HCT116 and HCT116b cells under growth factor deprivation stress (GFDS). (*A*) HCT116 cells were observed to be highly motile in comparison to HCT116b cells as demonstrated by Transwell Assay. (*B*) HCT116 cells upregulated the invasion-motility associated proteins p-Fak, p-Paxillin, RhoA, Rac1 and Rock1 under GFDS conditions. In contrast, HCT116b cells downregulated RhoA, Rac1 and Rock1 proteins and showed a lower increase of p-Fak and p-Paxillin under similar conditions.

Invasion and migration are the two initial steps that facilitate the multi-step cascade of dissemination of cancer cells to distant organ sites [19]. We determined the migratory properties of HCT116 and HCT116b cells under conditions of GFDS for 5 days using the transwell motility assay as described in the materials and methods section. Under GFDS, the HCT116b showed approximately 90% reduction in motility compared to HCT116 cells (Figure 6A). To identify the contributions of oncogenic signaling molecules in regulating motility of these cells, we performed western blot analysis and analyzed for several biochemical markers associated with invasion and migration of cancer cells. RhoA, ROCK1 and Rac1 proteins are GTPases that regulate F-actin and thereby regulate cancer cell migration and have been implicated in metastasis of several types of cancer including CRC [19]. As described in Figure 6B, RhoA, Rac1 and ROCK1 protein expression decreased in HCT116b cells under GFDS. In contrast, under stress conditions there was an increase in the protein expression of these GTPases in HCT116 cells. We further observed an increase in the phosphorylation of FAK and paxillin in HCT116 cells under GFDS that are also implicated in CRC progression and metastasis [20].

## Discussion

In this study, we explored the phenotypic heterogeneity of clonal variants isolated from the same patient primary colon carcinoma using an orthotopic mouse metastasis model for studying CRC metastasis. As evident from the recent review by Marusyk et al [4], there are both genetic and non-genetic causes of phenotypic heterogeneity of cancer cells. The intra-tumoral heterogeneity has substantial implication in the prognosis, subtype categorization, risk of metastasis and the development of therapeutic resistance [4]. Inter-tumoral heterogeneity has been well studied and has provided several model systems to study the initiation and progression of cancer. However, the drawback of these model systems is that the comparison of non-isogenic cell lines has the potential to generate erroneous conclusions since the biological and growth regulatory properties may reflect individual variations of such cells [12].

We utilized the iso-clonal human colon carcinoma cell lines HCT116 and HCT116b to study the intra-tumoral heterogeneity in colon cancer cells. These cell lines were isolated and developed into primary tissue cultures directly from a single primary tumor, thus providing a unique opportunity to assess iso-clonal variations in genetic heterogeneity in an *in vivo* metastatic setting. Initial studies comparing these two clones showed differences in their cellular morphology [11]. The HCT116b cells were differentiated and weakly tumorigenic in comparison with the HCT116 cell lines [11]. HCT116 cells have been extensively characterized and demonstrated exogenous growth factors independence for DNA synthesis and growth. Awwad et al [12] made the striking observation that endogenously produced growth factor activation in colon cancer cells lead to their exogenous growth factor independence. These cells can survive under nutrient and growth factor deprivation in comparison to their iso-clonal variants that are growth-factor dependent. This report demonstrated that the development of growth factor independent phenotype represents clonal progression within the tumor *in vivo*.

This study investigated the metastatic properties of the two iso-clones utilizing an *in vivo* mouse metastasis model developed in our laboratory. This model utilizes orthotopic xenograft transplants from human CRC cell lines that recapitulate the nature of metastatic pattern of the disease in CRC patients in that liver and lung metastases are observed after the growth of primary tumors in the colon [1], [6], [7], [8], [9], [10]. The HCT116 cells were found to be highly metastatic with a metastatic rate of 68% in liver and/or lungs. The iso-clonal weakly tumorigenic and differentiated cell line HCT116b was found to be poorly metastatic with a metastatic colonization rate of about 4%. We observed high variability in the primary tumor burden between the two experimental groups (HCT116 and HCT116b) as well as within the same group receiving orthotopically implanted xenograft tumors (HCT116 or HCT116b). Due to this inter- and intra-tumor variability in tumor burden, we performed Ki-67 staining to measure the cell proliferation rate between several HCT116 and HCT116b primary tumors in order to address the possibility that higher level of metastasis might be a consequence of increased primary tumor burden. Interestingly, HCT116 and HCT116b implanted animals had no difference in their rate of cell proliferation in the primary tumors, irrespective of the tumor burden as indicated by Ki-67 staining. However, HCT116 primary tumors have a significantly lower level of TUNEL staining indicative of low apoptotic index, and enhanced cell survival capabilities compared to HCT116b primary tumors.

In this work, we made the novel observation that both iso-clonal variants HCT116 and HCT116b cells showed 100% primary tumor invasion but different rates of metastases. Further analysis of the HCT116 and HCT116b primary colonic tumors showed similar rates of cell proliferation as demonstrated by Ki-67 staining. In contrast, these tumors showed clonal variability in their cell survival capabilities. HCT116b primary tumors showed increased TUNEL staining, which is indicative of cell death compared to HCT116 primary tumors. These findings indicate that the HCT116 and HCT116b cells have developed variability in their ability to form progressively growing colonies at the distal sites rather than modulating their dissemination from the primary tumors sites. This result also suggested that the low rate of distant metastasis in HCT116b cells might be related to stress-induced cell death in the foreign microenvironment for growth of the colon cancer cells in the liver and/or lungs. As evident from the *in vitro* data presented in Figures 4–6, induction of stress by growth factor deprivation in HCT116b cells led to increased cell death through the downregulation of several key oncogenic molecules that are known to participate in the regulation of aberrant cell survival and metastasis. Ki-67 and TUNEL staining has been used extensively in numerous clinical and pre-clinical studies. Ki-67 has been clinically relevant having predictive and prognostic value. In contrast, TUNEL staining, which is indicative of apoptotic index has been extensively linked to patient survival. The results obtained in this study are significant because they raise the clinical importance of cell survival signaling that could be playing a major role in the dissemination of colon cancer cells. Therefore, successful targeting of these pathways would be an effective strategy to target the treatment of metastasis.

We assessed the gene signatures associated with these clones using microarray and bioinformatics analysis. As represented in the heat map dendrogram (Figure S1), as expected numerous genes were differentially regulated between HCT116 and HCT116b cells. Several of these genes are markers associated with increased stemness, angiogenesis, invasion-migration, growth, survival and metastasis of the HCT116 clones. We focused our attention specifically to the genes affecting the cell survival and metastasis functions of CRC. The linkage of aberrant cell survival signaling for various steps in metastasis has been established with particular emphasis on the importance of aberrant cell survival to successful colonization at the metastatic site [21]. Therefore, the understanding of mechanisms that govern cell survival fate of highly metastatic cells would lead to the understanding of a new paradigm for control of metastatic potential and would provide the basis for developing novel strategies for the treatment of metastases. Several growth factor signaling pathways were differentially regulated between the HCT116 and HCT116b cells analyzed under GFDS conditions for 3 to 5 days. An important criterion selected for this study was the selection of up to Day 5 under quiescence state (Q5) as the time at which the experiments were performed. Previous work by Wang et al [22] has shown that at this stage, these cells show a minimal baseline incorporation of ^3^H thymidine yet retain greater than 95% viability. Basal ^3^H thymidine incorporation is minimal because nutrients from the growth factor free culture medium have been exhausted. ^3^H thymidine incorporation is restored by addition of fresh nutrients to these cells without any exogenous growth factors [23]. Restriction of nutrients past 5 days results in release of the cells from the culture plate and death. Therefore, we posited that operationally, 5 days post-confluency should be a time when the cell's endogenous cell survival and cell cycle re-entry capabilities are maximized. Since these functions are closely aligned with malignancy it is reasonable to project that the endogenous signaling mechanisms exhibited by these cells at this point are relevant to the maintenance of the metastatic phenotype of these cells. The PI3K/AKT, MAPK and PLC pathways are implicated in cell survival and metastasis of cancer cells. In this study, we demonstrate for the first time, the variation of activation of these signaling nodes under GFDS comparing the two iso-clonal sublines. We further demonstrated the increased expression of proteins involved in invasion-migration of CRC cells as a demonstration of the cancer cells extraordinary ability to evade stress mechanisms under anchorage-independent conditions.

The subject of intra-tumoral phenotypic heterogeneity in metastatic dissemination remains mostly unexplored. Marusyk et al [4] documented several distinct sources of phenotypic heterogeneity within tumor cell population. It has been reported that such variation in tumor population are an integration of multiple genetic and non-genetic factors. Previously, the association of intra-tumor heterogeneity with poor prognosis has been demonstrated in esophageal and breast cancer [24], [25]. In this study, we demonstrate the relevance of intra-tumoral heterogeneity in colorectal cancer metastasis using the orthotopic metastasis mouse model. The highly metastatic HCT116 clonal variant showed remarkable heterogeneity in gene signatures associated with cell survival and metastatic capabilities in comparison to the poorly metastatic HCT116b clones. The HCT116 cells displayed resistance to nutrient stress by upregulating the protein expression of several key oncogenes associated with aberrant cell survival and metastasis of CRC. In contrast, the poorly metastatic isoclonal variant HCT116b cells downregulated these proteins under similar GFDS conditions demonstrating strikingly opposite response to induce cell death under survival-associated stress.

## Supporting Information

Figure S1 **Gene expression analysis between HCT116 and HCT116b primary colon carcinoma tumor samples.**
(TIF)

Figure S2 **Gene ontology showing % difference in gene expression in different cellular compartments.**
(TIF)

Table S1 **List of genes differentially regulated between HCT116 and HCT116b primary colon carcinoma tumor samples.**
(PDF)

## References

[1] Chowdhury S, Howell GM, Rajput A, Teggart CA, Brattain LE, et al (2011) Identification of a novel TGFbeta/PKA signaling transduceome in mediating control of cell survival and metastasis in colon cancer. PLoS One 6: e19335. 21559296 10.1371/journal.pone.0019335 PMC3086924

[2] Markowitz SD, Bertagnolli MM (2009) Molecular origins of cancer: Molecular basis of colorectal cancer. N Engl J Med 361: 2449–2460. 20018966 10.1056/NEJMra0804588 PMC2843693

[3] TCGA (2012) Comprehensive molecular characterization of human colon and rectal cancer. Nature 487: 330–337. 22810696 10.1038/nature11252 PMC3401966

[4] Marusyk A, Almendro V, Polyak K (2012) Intra-tumour heterogeneity: a looking glass for cancer? Nat Rev Cancer 12: 323–334. 22513401 10.1038/nrc3261

[5] Guo XN, Rajput A, Rose R, Hauser J, Beko A, et al (2007) Mutant PIK3CA-bearing colon cancer cells display increased metastasis in an orthotopic model. Cancer Res 67: 5851–5858. 17575153 10.1158/0008-5472.CAN-07-0049

[6] Rajput A, Dominguez San Martin I, Rose R, Beko A, Levea C, et al (2008) Characterization of HCT116 human colon cancer cells in an orthotopic model. J Surg Res 147: 276–281. 17961596 10.1016/j.jss.2007.04.021

[7] Rajput A, Koterba AP, Kreisberg JI, Foster JM, Willson JK, et al (2007) A novel mechanism of resistance to epidermal growth factor receptor antagonism in vivo. Cancer Res 67: 665–673. 17234777 10.1158/0008-5472.CAN-06-2773

[8] Wang J, Kuropatwinski K, Hauser J, Rossi MR, Zhou Y, et al (2007) Colon carcinoma cells harboring PIK3CA mutations display resistance to growth factor deprivation induced apoptosis. Mol Cancer Ther 6: 1143–1150. 17363507 10.1158/1535-7163.MCT-06-0555

[9] Wang J, Rajput A, Kan JL, Rose R, Liu XQ, et al (2009) Knockdown of Ron kinase inhibits mutant phosphatidylinositol 3-kinase and reduces metastasis in human colon carcinoma. J Biol Chem 284: 10912–10922. 19224914 10.1074/jbc.M809551200 PMC2667777

[10] Ongchin M, Sharratt E, Dominguez I, Simms N, Wang J, et al (2009) The effects of epidermal growth factor receptor activation and attenuation of the TGFbeta pathway in an orthotopic model of colon cancer. J Surg Res 156: 250–256. 19524264 10.1016/j.jss.2009.02.002

[11] Brattain MG, Marks ME, McCombs J, Finely W, Brattain DE (1983) Characterization of human colon carcinoma cell lines isolated from a single primary tumour. Br J Cancer 47: 373–381. 6830688 10.1038/bjc.1983.56 PMC2011309

[12] Awwad RA, Sergina N, Yang H, Ziober B, Willson JK, et al (2003) The role of transforming growth factor alpha in determining growth factor independence. Cancer Res 63: 4731–4738. 12907656

[13] Boyd DD, Levine AE, Brattain DE, McKnight MK, Brattain MG (1988) Comparison of growth requirements of two human intratumoral colon carcinoma cell lines in monolayer and soft agarose. Cancer Res 48: 2469–2474. 3281751

[14] Brattain MG, Levine AE, Chakrabarty S, Yeoman LC, Willson JK, et al (1984) Heterogeneity of human colon carcinoma. Cancer Metastasis Rev 3: 177–191. 6437669 10.1007/BF00048384

[15] Chowdhury S, Howell GM, Teggart CA, Chowdhury A, Person JJ, et al (2011) Histone deacetylase inhibitor belinostat represses survivin expression through reactivation of transforming growth factor beta (TGFbeta) receptor II leading to cancer cell death. J Biol Chem 286: 30937–30948. 21757750 10.1074/jbc.M110.212035 PMC3162453

[16] Wang J, Yang L, Yang J, Kuropatwinski K, Wang W, et al (2008) Transforming growth factor beta induces apoptosis through repressing the phosphoinositide 3-kinase/AKT/survivin pathway in colon cancer cells. Cancer Res 68: 3152–3160. 18451140 10.1158/0008-5472.CAN-07-5348

[17] Fang JY, Richardson BC (2005) The MAPK signalling pathways and colorectal cancer. Lancet Oncol 6: 322–327. 15863380 10.1016/S1470-2045(05)70168-6

[18] Noh DY, Lee YH, Kim SS, Kim YI, Ryu SH, et al (1994) Elevated content of phospholipase C-gamma 1 in colorectal cancer tissues. Cancer 73: 36–41. 8275435 10.1002/1097-0142(19940101)73:1<36::aid-cncr2820730108>3.0.co;2-5

[19] Gulhati P, Bowen KA, Liu J, Stevens PD, Rychahou PG, et al (2011) mTORC1 and mTORC2 regulate EMT, motility, and metastasis of colorectal cancer via RhoA and Rac1 signaling pathways. Cancer Res 71: 3246–3256. 21430067 10.1158/0008-5472.CAN-10-4058 PMC3085654

[20] Crowe DL, Ohannessian A (2004) Recruitment of focal adhesion kinase and paxillin to beta1 integrin promotes cancer cell migration via mitogen activated protein kinase activation. BMC Cancer 4: 18. 15132756 10.1186/1471-2407-4-18 PMC416481

[21] Mehlen P, Puisieux A (2006) Metastasis: a question of life or death. Nat Rev Cancer 6: 449–458. 16723991 10.1038/nrc1886

[22] Wang D, Patil S, Li W, Humphrey LE, Brattain MG, et al (2002) Activation of the TGFalpha autocrine loop is downstream of IGF-I receptor activation during mitogenesis in growth factor dependent human colon carcinoma cells. Oncogene 21: 2785–2796. 11973637 10.1038/sj.onc.1205375

[23] Jiang D, Yang H, Willson JK, Liang J, Humphrey LE, et al (1998) Autocrine transforming growth factor alpha provides a growth advantage to malignant cells by facilitating re-entry into the cell cycle from suboptimal growth states. J Biol Chem 273: 31471–31479. 9813060 10.1074/jbc.273.47.31471

[24] Maley CC, Galipeau PC, Finley JC, Wongsurawat VJ, Li X, et al (2006) Genetic clonal diversity predicts progression to esophageal adenocarcinoma. Nat Genet 38: 468–473. 16565718 10.1038/ng1768

[25] Park SY, Gonen M, Kim HJ, Michor F, Polyak K (2010) Cellular and genetic diversity in the progression of in situ human breast carcinomas to an invasive phenotype. J Clin Invest 120: 636–644. 20101094 10.1172/JCI40724 PMC2810089

