# Supplementary figures and images for: Intra-Tumoral Heterogeneity in Metastatic Potential and Survival Signaling between Iso-Clonal HCT116 and HCT116b Human Colon Carcinoma Cell Lines
Source: PLoS One. 2013 Apr 1;8(4):e60299. doi: 10.1371/journal.pone.0060299 (PMC3613369; doi:10.1371/journal.pone.0060299)

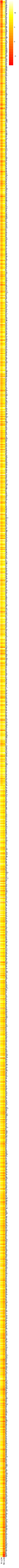

Supplement: Figure S1 — Gene expression analysis between HCT116 and HCT116b primary colon carcinoma tumor samples. (TIF) [file pone.0060299.s001.tif]

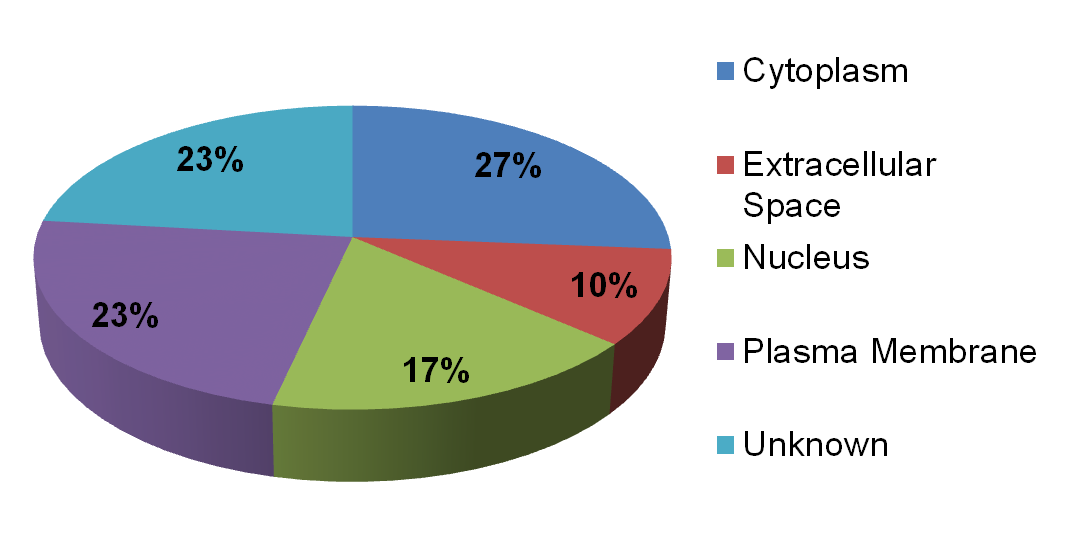

Supplement: Figure S2 — Gene ontology showing % difference in gene expression in different cellular compartments. (TIF) [file pone.0060299.s002.tif]
