# Supplementary material for: Intra-Tumoral Heterogeneity in Metastatic Potential and Survival Signaling between Iso-Clonal HCT116 and HCT116b Human Colon Carcinoma Cell Lines
Source: PLoS One. 2013 Apr 1;8(4):e60299. doi: 10.1371/journal.pone.0060299 (PMC3613369; doi:10.1371/journal.pone.0060299)
Supplement: Table S1 — List of genes differentially regulated between HCT116 and HCT116b primary colon carcinoma tumor samples. (PDF) [file pone.0060299.s003.pdf]

SYMBOL	HCT116.AV	HCT116b.A	HCT116b/H	SEARCH_KEY	ILMN_GENE	CHROMOSOME
GPR83	551.6559	0.004047	0	ILMN_165214	GPR83	11
CST6	2841.894	0.125638	0	ILMN_20022	CST6	
CYP24A1	5395.758	0.446911	0	ILMN_21296	CYP24A1	20
PLA2G7	1007.268	0.122711	0	ILMN_29396	PLA2G7	6
PTGER3	27.36418	0.009324	0	ILMN_11251	PTGER3	1
IGSF2	72.50452	0.035216	0	ILMN_24629	IGSF2	
NAP1L5	1254.937	0.853378	0	ILMN_5355	NAP1L5	4
LAIR2	116.3532	0.098785	0	ILMN_2857	LAIR2	19
EREG	545.5273	0.500692	0	ILMN_17131	EREG	4
MMP10	750.5863	0.750928	0	ILMN_4442	MMP10	11
FGG	73.12473	0.07544	0	ILMN_25813	FGG	4
NOS1	1183.164	1.234322	0	ILMN_12873	NOS1	12
RFTN1	1197.104	1.283154	0	ILMN_29470	RFTN1	3
PON3	4614.394	5.043413	0	ILMN_19416	PON3	7
ARMCX5	2022.116	2.843327	0	ILMN_9239	ARMCX5	X
DUOXA1	658.1518	1.00064	0	ILMN_28994	DUOXA1	15
RCAN2	2272.713	3.596267	0	ILMN_25728	RCAN2	6
OR2AE1	801.6594	1.291675	0	ILMN_10081	OR2AE1	7
TLL1	2738.881	4.429685	0	ILMN_15434	TLL1	4
OR51G1	112.1309	0.193313	0	ILMN_13587	OR51G1	11
LILRA5	129.1529	0.240046	0	ILMN_13562	LILRA5	19
MARK1	3146.739	6.609578	0	ILMN_26873	MARK1	1
GMDS	5148.389	11.11692	0	ILMN_16535	GMDS	6
B3GALNT1	1113.639	2.508816	0	ILMN_136965	B3GALNT1	3
RGR	408.8005	0.922998	0	ILMN_28272	RGR	10
PHLDA3	45.55379	0.106473	0	ILMN_24559	PHLDA3	1
HLA-DRA	3167.342	7.460907	0	ILMN_7314	HLA-DRA	6
MEIS2	1064.935	2.618348	0	ILMN_180109	MEIS2	15
RAB7B	689.1187	1.871226	0	ILMN_11485	RAB7B	
COL24A1	565.3725	1.595936	0	ILMN_14134	COL24A1	1
LOC285735	814.9795	2.334117	0	ILMN_1408	LOC285735	6
BVES	269.6741	0.773192	0	ILMN_25037	BVES	6
DTNA	1373.94	4.400959	0	ILMN_28503	DTNA	18
OR4K5	271.1867	0.924506	0	ILMN_162937	OR4K5	14
CYP4F11	2032.022	7.002419	0	ILMN_9376	CYP4F11	19
SOX11	259.316	0.896821	0	ILMN_28038	SOX11	2
NR5A2	2446.363	8.893986	0	ILMN_14912	NR5A2	1
C5orf4	233.2111	0.853515	0	ILMN_21073	C5ORF4	5
RGS17	1915.225	7.428099	0	ILMN_25210	RGS17	6
PXT1	140.674	0.550488	0	ILMN_21991	PXT1	6
KRT6C	1052.575	4.149243	0	ILMN_1845	KRT6C	
CYP3A7	319.5592	1.311278	0	ILMN_15765	CYP3A7	7
EPDR1	3902.971	16.60527	0	ILMN_25170	EPDR1	7
EFCAB6	173.4907	0.781179	0	ILMN_5427	EFCAB6	22
OR51I1	744.8152	3.403759	0	ILMN_10084	OR51I1	11
FAP	211.4364	1.014369	0	ILMN_14995	FAP	2

TACSTD2	2225.635	11.03903	0	ILMN_4004	TACSTD2	1
ZNF311	172.124	0.875472	0.01	ILMN_13954	ZNF311	6
TNR	329.84	1.704301	0.01	ILMN_5903	TNR	1
GLT8D2	1715.034	9.033575	0.01	ILMN_27948	GLT8D2	12
CHST3	1166.716	6.455035	0.01	ILMN_7808	CHST3	10
EYA2	58.84451	0.334531	0.01	ILMN_13037	EYA2	20
HLA-DPB1	133.9014	0.768936	0.01	ILMN_4641	HLA-DPB1	6
C6orf105	335.993	1.956242	0.01	ILMN_10287	C6ORF105	6
LALBA	376.3631	2.203944	0.01	ILMN_10013	LALBA	12
VNN1	363.7191	2.171805	0.01	ILMN_14011	VNN1	6
RBP1	4844.168	29.97048	0.01	ILMN_11289	RBP1	3
CPNE8	813.3182	5.146533	0.01	ILMN_20089	CPNE8	12
LIFR	1420.495	9.234753	0.01	ILMN_5930	LIFR	5
ENAM	65.36152	0.43402	0.01	ILMN_21621	ENAM	4
PCDHA10	142.1183	0.955198	0.01	ILMN_29129	PCDHA10	5
DACT1	663.4476	4.578847	0.01	ILMN_2806	DACT1	14
OR2V2	324.4865	2.305798	0.01	ILMN_5760	OR2V2	5
SLC16A7	112.9646	0.804065	0.01	ILMN_21160	SLC16A7	12
STARD3NL	4422.597	31.70123	0.01	ILMN_16121	STARD3NL	7
GPRASP2	526.8751	3.845174	0.01	ILMN_8313	GPRASP2	X
TPRG1	1378.732	10.19514	0.01	ILMN_16824	TPRG1	3
IFNA10	1.736827	0.013238	0.01	ILMN_4745	IFNA10	9
DUSP15	136.076	1.045346	0.01	ILMN_15949	DUSP15	20
XKR5	103.9051	0.807993	0.01	ILMN_13379	XKR5	8
RBM35B	120.4596	0.960575	0.01	ILMN_3310	RBM35B	16
LOC650137	63.83563	0.511046	0.01	ILMN_167125	LOC650137	
ZNF182	606.3301	4.859676	0.01	ILMN_19238	ZNF182	X
ZNF167	143.3514	1.160364	0.01	ILMN_26551	ZNF167	3
SIRPB1	792.8237	6.42727	0.01	ILMN_12074	SIRPB1	20
DKK3	23.4354	0.194575	0.01	ILMN_4127	DKK3	11
CALCRL	124.2016	1.036227	0.01	ILMN_18176	CALCRL	2
XIRP2	275.4056	2.32572	0.01	ILMN_19224	XIRP2	2
DAB2	678.4193	5.890546	0.01	ILMN_162394	DAB2	5
KCNC2	260.7492	2.318166	0.01	ILMN_8170	KCNC2	12
LOC392196	314.7006	2.804825	0.01	ILMN_180854	LOC392196	8
HS6ST3	21.92587	0.198902	0.01	ILMN_137777	HS6ST3	13
CYP2C19	1828.328	16.7109	0.01	ILMN_21019	CYP2C19	10
LY96	1049.208	9.801606	0.01	ILMN_25195	LY96	8
OR5D16	101.7615	0.957793	0.01	ILMN_168082	OR5D16	11
KIR3DL1	770.9736	7.264228	0.01	ILMN_13167	KIR3DL1	19
LOC440313	323.4843	3.056843	0.01	ILMN_20538	LOC440313	15
IL17F	252.6964	2.429281	0.01	ILMN_2831	IL17F	6
BDNF	512.423	4.930249	0.01	ILMN_9285	BDNF	11
KLRC2	449.7177	4.338603	0.01	ILMN_165739	KLRC2	12
EGF	6.88075	0.068156	0.01	ILMN_611	EGF	4
MYO18B	162.6431	1.621058	0.01	ILMN_8137	MYO18B	22
LMCD1	346.0201	3.497247	0.01	ILMN_20151	LMCD1	3

VPS33A	8.867121	0.089885	0.01	ILMN_12833	VPS33A	12
TRIML2	1168.276	12.07086	0.01	ILMN_24301	TRIML2	4
FAR2	1071.542	11.28076	0.01	ILMN_175721	FAR2	12
HTR7P	31.0893	0.331305	0.01	ILMN_8591	HTR7P	12
MMP13	829.2567	8.885175	0.01	ILMN_18653	MMP13	11
OR4M2	10.22033	0.114557	0.01	ILMN_183196	OR4M2	15
LOC642316	346.8317	3.891601	0.01	ILMN_139322	LOC642316	6
EDNRA	1028.226	11.62133	0.01	ILMN_8606	EDNRA	4
KRT222P	915.8029	10.41156	0.01	ILMN_28583	KRT222P	17
TFPI	1552.063	17.71201	0.01	ILMN_1429	TFPI	2
GSG1	82.92382	0.965393	0.01	ILMN_2782	GSG1	12
ARSJ	542.3002	6.363364	0.01	ILMN_24631	ARSJ	4
KRTAP17-1	280.4065	3.327257	0.01	ILMN_19903	KRTAP17-1	17
LOC728643	212.6564	2.527154	0.01	ILMN_183126	LOC728643	10
HLA-G	33.95865	0.405146	0.01	ILMN_1631	HLA-G	6
ADRA1B	834.6147	10.08181	0.01	ILMN_10629	ADRA1B	5
DEFB104B	14.3907	0.174017	0.01	ILMN_174773	DEFB104B	8
COL4A5	1429.732	17.32847	0.01	ILMN_13514	COL4A5	X
KRT5	312.2382	3.846105	0.01	ILMN_7654	KRT5	12
GATA5	598.5041	7.37698	0.01	ILMN_25192	GATA5	20
BHLHB9	1009.62	12.53531	0.01	ILMN_3098	BHLHB9	X
CHRM2	253.9489	3.1908	0.01	ILMN_9979	CHRM2	7
KRTAP3-2	536.6614	6.88075	0.01	ILMN_23670	KRTAP3-2	17
OR2T3	302.433	3.969443	0.01	ILMN_12536	OR2T3	1
SLC36A4	3404.626	45.33236	0.01	ILMN_13325	SLC36A4	11
KRT1	1296.615	17.31953	0.01	ILMN_11426	KRT1	12
PENK	18.26666	0.250943	0.01	ILMN_9859	PENK	8
RAG2	495.0397	6.854177	0.01	ILMN_888	RAG2	11
OR52N4	463.9017	6.43881	0.01	ILMN_9852	OR52N4	11
TFAP2B	553.73	8.014692	0.01	ILMN_13391	TFAP2B	6
HIST2H2BF	520.3618	7.535169	0.01	ILMN_138755	HIST2H2BF	1
CPA4	3648.163	52.85949	0.01	ILMN_21403	CPA4	7
ARHGEF6	1878.52	27.2187	0.01	ILMN_26689	ARHGEF6	X
FHL1	773.9222	11.22957	0.01	ILMN_7975	FHL1	X
SERPINA1	173.1567	2.567097	0.01	ILMN_1034	SERPINA1	14
SPRR4	479.1026	7.175176	0.01	ILMN_8890	SPRR4	1
OR2L1P	122.8908	1.84555	0.02	ILMN_12923	OR2L1P	1
OR51M1	69.44671	1.045403	0.02	ILMN_22582	OR51M1	11
CYP2B7P1	427.8904	6.522484	0.02	ILMN_4317	CYP2B7P1	19
KIR2DL3	468.4825	7.235121	0.02	ILMN_27681	KIR2DL3	19
PSG7	157.0645	2.433393	0.02	ILMN_28896	PSG7	19
FUT11	11.4673	0.179595	0.02	ILMN_25595	FUT11	10
SERPINB7	222.6763	3.504172	0.02	ILMN_8141	SERPINB7	18
CCRL1	18.4669	0.29262	0.02	ILMN_7293	CCRL1	3
SNAI2	809.7078	13.1998	0.02	ILMN_165913	SNAI2	8
IGLL3	17.64905	0.289032	0.02	ILMN_167544	IGLL3	22
SLC35F1	297.6691	4.918061	0.02	ILMN_2907	SLC35F1	6

APOBEC3H	1267.115	20.99158	0.02	ILMN_18618	APOBEC3H	22
KIR3DL3	839.9667	14.00237	0.02	ILMN_18412	KIR3DL3	19
GDF3	198.6153	3.332535	0.02	ILMN_22815	GDF3	12
ATP12A	102.4971	1.759022	0.02	ILMN_138153	ATP12A	13
KCNIP4	139.1333	2.446766	0.02	ILMN_13199	KCNIP4	4
RAB38	3036.033	53.66138	0.02	ILMN_22951	RAB38	11
VIM	1428.126	25.34926	0.02	ILMN_676	VIM	10
WFDC10A	547.701	9.77655	0.02	ILMN_9388	WFDC10A	20
DIRAS3	777.5012	13.98006	0.02	ILMN_13884	DIRAS3	1
OR5AU1	192.7189	3.471448	0.02	ILMN_19367	OR5AU1	14
SLC8A3	181.1044	3.280762	0.02	ILMN_24218	SLC8A3	14
KRT74	314.3813	5.713742	0.02	ILMN_1002	KRT74	12
C10orf90	278.2989	5.060614	0.02	ILMN_21345	C10ORF90	10
SSPN	553.5358	10.11497	0.02	ILMN_20454	SSPN	12
FGF2	126.8136	2.345189	0.02	ILMN_167999	FGF2	4
KIAA0319	249.4818	4.645969	0.02	ILMN_19660	KIAA0319	6
KCTD14	2510.319	46.9498	0.02	ILMN_2390	KCTD14	11
SLC12A5	477.6405	9.127589	0.02	ILMN_165817	SLC12A5	20
MAP1B	468.912	9.049417	0.02	ILMN_28251	MAP1B	5
TIAM2	422.7282	8.272428	0.02	ILMN_9891	TIAM2	6
GPR135	41.88134	0.822914	0.02	ILMN_9642	GPR135	14
SLC43A3	726.425	14.33879	0.02	ILMN_26686	SLC43A3	11
ATP8A2	154.0124	3.079171	0.02	ILMN_11255	ATP8A2	13
M160	8270.408	165.6355	0.02	ILMN_137163	M160	12
CPLX3	105.3334	2.118767	0.02	ILMN_19503	CPLX3	15
HBG2	1532.738	31.1372	0.02	ILMN_28292	HBG2	11
MYH11	234.3203	4.780378	0.02	ILMN_176190	MYH11	16
KCNMB1	262.4201	5.363291	0.02	ILMN_8822	KCNMB1	5
MC4R	41.04934	0.845564	0.02	ILMN_8243	MC4R	18
NLRP4	74.18	1.535476	0.02	ILMN_13312	NLRP4	19
OR3A4	35.95375	0.747311	0.02	ILMN_2334	OR3A4	17
SCN4A	253.441	5.305214	0.02	ILMN_5200	SCN4A	17
COL6A3	901.1021	19.07614	0.02	ILMN_178962	COL6A3	2
ZNF655	284.4455	6.061713	0.02	ILMN_3736	ZNF655	7
SLC13A3	290.5073	6.22354	0.02	ILMN_13280	SLC13A3	20
PTPRM	1314.901	28.77104	0.02	ILMN_19957	PTPRM	18
LOC402176	131.8165	2.897397	0.02	ILMN_3279	LOC402176	4
GRM7	45.27626	0.996203	0.02	ILMN_19112	GRM7	3
NR4A3	26.68562	0.593006	0.02	ILMN_804	NR4A3	9
C20orf77	552.2433	12.39902	0.02	ILMN_29248	C20ORF77	20
OR51B6	427.0648	9.627498	0.02	ILMN_17335	OR51B6	11
C9orf135	56.75076	1.287386	0.02	ILMN_14238	C9ORF135	9
GHRH	93.67206	2.125892	0.02	ILMN_20345	GHRH	20
MYOZ3	104.1756	2.370686	0.02	ILMN_21305	MYOZ3	5
HIVEP3	76.54904	1.751111	0.02	ILMN_19427	HIVEP3	1
MGC33846	146.2262	3.345973	0.02	ILMN_8275	MGC33846	11
C1orf110	380.6707	8.827109	0.02	ILMN_14513	C1ORF110	1

EDAR	151.9346	3.528654	0.02	ILMN_29351	EDAR	2
COLEC12	55.6139	1.292032	0.02	ILMN_24713	COLEC12	18
TNFSF4	1237.473	29.01362	0.02	ILMN_8432	TNFSF4	1
LOC731102	587.5538	14.15484	0.02	ILMN_19715	LOC731102	
CLCA2	970.0165	23.64096	0.02	ILMN_25090	CLCA2	1
LOC344405	565.939	13.80613	0.02	ILMN_173509	LOC344405	
TCTEX1D4	135.4119	3.320619	0.02	ILMN_168255	TCTEX1D4	1
CHRM1	157.3207	3.866781	0.02	ILMN_22402	CHRM1	11
LYL1	430.1178	10.65809	0.02	ILMN_18317	LYL1	
MMRN2	92.25278	2.309356	0.03	ILMN_2294	MMRN2	10
BANF2	139.9501	3.531882	0.03	ILMN_5162	BANF2	20
LCP1	1493.104	37.72462	0.03	ILMN_28547	LCP1	13
SERPINA5	2242.517	56.66032	0.03	ILMN_4712	SERPINA5	14
KIR2DL1	50.08091	1.270384	0.03	ILMN_7929	KIR2DL1	19|NT_113949.1
C19orf23	1015.852	25.96296	0.03	ILMN_11822	C19ORF23	19
HTR3C	335.8026	8.669592	0.03	ILMN_15908	HTR3C	3
F8	23.25314	0.604085	0.03	ILMN_30362	F8	X
PPEF1	98.38948	2.557831	0.03	ILMN_10993	PPEF1	X
PTAFR	71.78413	1.884213	0.03	ILMN_2949	PTAFR	1
EYA1	221.6808	5.826944	0.03	ILMN_17045	EYA1	8
FCRL6	259.0705	6.823588	0.03	ILMN_2355	FCRL6	1
ARVP6125	66.03602	1.741208	0.03	ILMN_18379	ARVP6125	3
GPR87	2320.501	61.64067	0.03	ILMN_23242	GPR87	3
TMEM200A	3764.057	100.2041	0.03	ILMN_19059	TMEM200A	6
FAM181A	28.84883	0.786388	0.03	ILMN_4172	FAM181A	14
KRTAP4-12	149.1648	4.118058	0.03	ILMN_2624	KRTAP4-12	17
KIR2DS5	1301.135	35.94343	0.03	ILMN_20102	KIR2DS5	19
CALD1	1094.373	30.23987	0.03	ILMN_29896	CALD1	7
CSN3	569.7286	15.77036	0.03	ILMN_13349	CSN3	4
C13orf31	247.6576	6.876957	0.03	ILMN_29054	C13ORF31	13
WDR16	45.05123	1.256776	0.03	ILMN_170497	WDR16	17
HBE1	3584.89	100.0248	0.03	ILMN_27655	HBE1	11
OPTC	25.74469	0.719538	0.03	ILMN_17113	OPTC	1
PCDHAC2	92.81035	2.636793	0.03	ILMN_9742	PCDHAC2	
LSP1	6.384946	0.181754	0.03	ILMN_25096	LSP1	11
SCUBE2	122.2039	3.485564	0.03	ILMN_14767	SCUBE2	11
FSTL1	1267.336	36.43143	0.03	ILMN_15510	FSTL1	3
FBXW12	5.94704	0.171146	0.03	ILMN_10121	FBXW12	3
OR51B2	149.5999	4.309624	0.03	ILMN_20840	OR51B2	
NPY	33.36719	0.963526	0.03	ILMN_11990	NPY	7
SMAD1	47.96068	1.385319	0.03	ILMN_7867	SMAD1	4
MMP7	159.8326	4.660227	0.03	ILMN_9188	MMP7	11
PKIA	769.0372	22.45921	0.03	ILMN_19703	PKIA	8
LOC441426	624.3619	18.54822	0.03	ILMN_17781	LOC441426	9
KRTAP10-9	272.8228	8.174793	0.03	ILMN_20172	KRTAP10-9	21
OR1K1	63.80814	1.925147	0.03	ILMN_19449	OR1K1	9
LONRF2	211.9899	6.450698	0.03	ILMN_19670	LONRF2	2

ARGFX	364.5954	11.13005	0.03	ILMN_29168	ARGFX	3
PSG2	161.8996	4.978446	0.03	ILMN_1426	PSG2	19
OR2T35	113.0059	3.493293	0.03	ILMN_182519	OR2T35	1
C8orf46	194.0585	6.119962	0.03	ILMN_7220	C8ORF46	8
ABCC2	2476.727	78.16281	0.03	ILMN_9691	ABCC2	10
THAP8	37.68907	1.199141	0.03	ILMN_25578	THAP8	19
TACR3	325.93	10.44541	0.03	ILMN_19640	TACR3	4
SPATA8	1129.391	36.65689	0.03	ILMN_22251	SPATA8	15
FAM83A	202.5072	6.590465	0.03	ILMN_14524	FAM83A	8
CHN2	467.62	15.24974	0.03	ILMN_16218	CHN2	7
TMEM31	373.9353	12.2012	0.03	ILMN_18732	TMEM31	X
SEC31B	164.6523	5.444165	0.03	ILMN_24649	SEC31B	10
ERMN	108.1193	3.579366	0.03	ILMN_13253	ERMN	2
KIAA1432	65.11066	2.160222	0.03	ILMN_26728	KIAA1432	9
POU6F1	4.831552	0.160763	0.03	ILMN_19216	POU6F1	12
IL12B	96.45706	3.2433	0.03	ILMN_4230	IL12B	5
SSX1	1835.695	61.85378	0.03	ILMN_169082	SSX1	X
NLGN1	300.7347	10.25147	0.03	ILMN_15512	NLGN1	3
ZNF780B	115.3903	3.958827	0.03	ILMN_24789	ZNF780B	19
SGCE	5155.09	177.0181	0.03	ILMN_13165	SGCE	7
NLGN3	241.821	8.375807	0.03	ILMN_10949	NLGN3	X
UCHL1	295.4392	10.26587	0.03	ILMN_9422	UCHL1	4
DNM3	286.1032	9.957741	0.03	ILMN_21225	DNM3	1
LOC441601	229.7259	8.017404	0.03	ILMN_181899	LOC441601	11
GRP	87.0884	3.044133	0.03	ILMN_23511	GRP	18
GPX5	196.072	6.872825	0.04	ILMN_8859	GPX5	6
SLC22A13	209.8773	7.367176	0.04	ILMN_24138	SLC22A13	3
RNASE13	125.8615	4.421	0.04	ILMN_27385	RNASE13	14
KRT23	875.8023	30.78719	0.04	ILMN_5602	KRT23	17
RPH3A	467.6284	16.46498	0.04	ILMN_13972	RPH3A	12
BEST2	38.81474	1.375589	0.04	ILMN_24139	BEST2	19
ANXA10	461.3886	16.3698	0.04	ILMN_20600	ANXA10	4
HBG1	696.5999	24.72452	0.04	ILMN_7520	HBG1	11
FBXO24	16.88124	0.600862	0.04	ILMN_3577	FBXO24	7
GRM3	600.4792	21.38947	0.04	ILMN_6748	GRM3	7
RAB34	4987.154	177.8697	0.04	ILMN_5179	RAB34	17
VSX1	36.75486	1.311166	0.04	ILMN_7096	VSX1	20
ST3GAL6	1679.532	59.95438	0.04	ILMN_2870	ST3GAL6	3
VSTM3	13.8781	0.49562	0.04	ILMN_1614	VSTM3	3
ADRB2	1735.107	62.12659	0.04	ILMN_2022	ADRB2	5
LPO	515.1758	18.50421	0.04	ILMN_28648	LPO	17
KRT85	52.48917	1.887841	0.04	ILMN_16826	KRT85	12
C8orf47	2215.464	79.96239	0.04	ILMN_183112	C8ORF47	8
TAS2R45	39.218	1.420131	0.04	ILMN_1709	TAS2R45	
HAL	1497.27	54.33877	0.04	ILMN_177429	HAL	12
TNS4	744.4307	27.04729	0.04	ILMN_27455	TNS4	
CD8A	69.34698	2.523954	0.04	ILMN_26107	CD8A	2

CPVL	877.6848	32.05089	0.04	ILMN_18740	CPVL	7
OR52R1	267.3031	9.762013	0.04	ILMN_9961	OR52R1	11
HDAC9	131.1113	4.794431	0.04	ILMN_26338	HDAC9	7
MCTP1	341.8062	12.51563	0.04	ILMN_3078	MCTP1	5
MCC	823.5238	30.27719	0.04	ILMN_17732	MCC	5
MAP2	293.8846	10.84915	0.04	ILMN_38764	MAP2	2
OR2B6	23.25677	0.859805	0.04	ILMN_10633	OR2B6	6
MYL4	44.05774	1.635328	0.04	ILMN_23542	MYL4	17
WFIKKN2	393.9293	14.68092	0.04	ILMN_18996	WFIKKN2	17
ANXA6	688.4156	25.68162	0.04	ILMN_3328	ANXA6	5
SLIT3	419.5089	15.72053	0.04	ILMN_18656	SLIT3	5
IGF1	5.527086	0.208174	0.04	ILMN_179483	IGF1	12
GFI1B	54.93371	2.070767	0.04	ILMN_21955	GFI1B	9
CREB5	209.337	7.906746	0.04	ILMN_19827	CREB5	7
OR52K2	472.996	17.94704	0.04	ILMN_4730	OR52K2	11
OR1S2	1081.234	41.12115	0.04	ILMN_180669	OR1S2	11
ISLR	50.88384	1.943318	0.04	ILMN_22659	ISLR	15
GALC	346.0268	13.32616	0.04	ILMN_138917	GALC	14
C10orf62	9.321294	0.359226	0.04	ILMN_12537	C10ORF62	10
OR2F1	24.03127	0.928904	0.04	ILMN_12803	OR2F1	7
TEX9	517.9807	20.0889	0.04	ILMN_6690	TEX9	15
TNNT3	42.79691	1.682004	0.04	ILMN_2581	TNNT3	11
NUDT12	500.3781	19.6732	0.04	ILMN_4699	NUDT12	5
OR10Z1	15.41452	0.613948	0.04	ILMN_27126	OR10Z1	1
ZNF608	994.0723	39.60991	0.04	ILMN_19900	ZNF608	5
CASQ2	51.58027	2.058517	0.04	ILMN_24210	CASQ2	1
OLR1	13.61185	0.543515	0.04	ILMN_17381	OLR1	12
KRT6B	3800.935	152.2798	0.04	ILMN_7664	KRT6B	12
C2orf27	456.8534	18.39111	0.04	ILMN_15235	C2ORF27	2
SALL2	45.1665	1.824782	0.04	ILMN_13166	SALL2	14
SIRPD	6.831645	0.276486	0.04	ILMN_19916	SIRPD	20
GPR65	28.88092	1.173752	0.04	ILMN_19139	GPR65	14
SERPINB10	33.87678	1.384695	0.04	ILMN_166006	SERPINB10	18
SPINK9	118.6874	4.855252	0.04	ILMN_170738	SPINK9	5
C1orf190	451.3964	18.49749	0.04	ILMN_8478	C1ORF190	1
ABCA6	104.3135	4.29426	0.04	ILMN_12413	ABCA6	17
ZNF418	288.2645	11.86946	0.04	ILMN_6197	ZNF418	19
FSD1L	419.0082	17.51365	0.04	ILMN_138096	FSD1L	
CHST1	14.01928	0.592261	0.04	ILMN_19267	CHST1	11
GPR82	318.6538	13.46576	0.04	ILMN_13231	GPR82	X
GPR81	88.64977	3.747866	0.04	ILMN_177777	GPR81	12
NKG7	1.496664	0.063278	0.04	ILMN_15943	NKG7	19
PLP1	12.63788	0.535481	0.04	ILMN_20597	PLP1	X
SST	569.5677	24.16199	0.04	ILMN_17603	SST	3
C17orf92	297.8216	12.67255	0.04	ILMN_28118	C17ORF92	17
SEPP1	199.9129	8.508516	0.04	ILMN_27164	SEPP1	5
SYN3	49.3443	2.122483	0.04	ILMN_165059	SYN3	22

STARD6	105.0205	4.542694	0.04	ILMN_20727	STARD6	18
OR5B2	62.29501	2.695144	0.04	ILMN_6573	OR5B2	11
OR51A7	85.07132	3.685304	0.04	ILMN_9033	OR51A7	11
LOR	574.7404	24.95939	0.04	ILMN_6861	LOR	1
SUNC1	296.0692	12.90024	0.04	ILMN_5330	SUNC1	7
ADAMTS16	30.35247	1.325779	0.04	ILMN_12104	ADAMTS16	5
MPPED2	200.6147	8.764702	0.04	ILMN_9856	MPPED2	11
CD36	300.1577	13.1145	0.04	ILMN_22014	CD36	7
FLJ40235	22.89035	1.006043	0.04	ILMN_29282	FLJ40235	19
MCAM	885.0778	39.20009	0.04	ILMN_27822	MCAM	11
CPEB1	67.5994	2.994544	0.04	ILMN_19747	CPEB1	15
CRISP3	206.3815	9.152687	0.04	ILMN_24733	CRISP3	6
CDX4	41.50955	1.841754	0.04	ILMN_22976	CDX4	X
FHOD3	4375.737	195.0818	0.04	ILMN_8268	FHOD3	18
DCT	80.11333	3.604244	0.04	ILMN_1796	DCT	13
DPPA2	450.7676	20.39042	0.05	ILMN_13691	DPPA2	3
WDR69	212.227	9.689503	0.05	ILMN_11451	WDR69	2
TLR9	68.06149	3.107447	0.05	ILMN_5498	TLR9	3
OR2T11	514.8676	23.51286	0.05	ILMN_13347	OR2T11	1
ATP2A1	149.0716	6.855304	0.05	ILMN_45810	ATP2A1	16
SPATA3	78.27145	3.600788	0.05	ILMN_22459	SPATA3	2
VGLL1	11.51623	0.531856	0.05	ILMN_13104	VGLL1	X
C14orf148	468.7158	21.67737	0.05	ILMN_13083	C14ORF148	14
SLC27A6	873.2966	40.50956	0.05	ILMN_10102	SLC27A6	5
NME1-NMI	20.44271	0.952594	0.05	ILMN_25788	NME1-NME2	17
TDGF1	4.684564	0.218377	0.05	ILMN_167634	TDGF1	3
KIF5C	1292.662	60.52357	0.05	ILMN_167649	KIF5C	2
KLHL3	41.19988	1.941513	0.05	ILMN_10714	KLHL3	5
LINGO4	537.6829	25.4884	0.05	ILMN_2002	LINGO4	1
SLC17A4	239.9818	11.4019	0.05	ILMN_7320	SLC17A4	6
MRAS	397.2466	18.95438	0.05	ILMN_19997	MRAS	3
PRSS27	390.1284	18.64271	0.05	ILMN_14247	PRSS27	16
FSIP2	56.22316	2.68771	0.05	ILMN_9469	FSIP2	2
FLJ35880	12.86355	0.616323	0.05	ILMN_3282	FLJ35880	3
OR5P2	208.4046	10.00174	0.05	ILMN_8855	OR5P2	11
CHST4	398.249	19.11425	0.05	ILMN_5660	CHST4	16
ADRA1A	66.37759	3.189822	0.05	ILMN_17568	ADRA1A	8
TNFRSF6B	1702.11	82.2317	0.05	ILMN_14212	TNFRSF6B	20
C20orf107	27.66755	1.34352	0.05	ILMN_1548	C20ORF107	20
OLFML3	838.5174	40.98874	0.05	ILMN_20333	OLFML3	1
SUSD2	9565.198	468.7158	0.05	ILMN_13115	SUSD2	22
GPR141	53.2266	2.619507	0.05	ILMN_20517	GPR141	7
LRFN2	668.5951	32.90929	0.05	ILMN_19564	LRFN2	6
GRAP	271.6818	13.42987	0.05	ILMN_5687	GRAP	17
IL1F5	438.8332	21.72453	0.05	ILMN_9939	IL1F5	2
N4BP2L1	252.0351	12.50833	0.05	ILMN_182459	N4BP2L1	13
OR5AY1	235.5221	11.70657	0.05	ILMN_20201	OR5AY1	1

TENC1	26.55836	1.328727	0.05	ILMN_9756	TENC1	12
SPINK7	149.8453	7.570376	0.05	ILMN_23556	SPINK7	5
TNFSF8	85.42312	4.315808	0.05	ILMN_658	TNFSF8	9
OR4K13	133.5526	6.753226	0.05	ILMN_2769	OR4K13	14
HIST1H2AA	76.16356	3.852922	0.05	ILMN_15137	HIST1H2AA	6
MYL2	64.29987	3.259404	0.05	ILMN_26696	MYL2	12
FGF19	602.9159	30.65872	0.05	ILMN_18897	FGF19	11
C6orf59	115.063	5.884462	0.05	ILMN_14101	C6ORF59	
CRYGD	21.9345	1.125838	0.05	ILMN_30150	CRYGD	2
KLHL32	84.29441	4.327681	0.05	ILMN_5846	KLHL32	
FMO6P	1.326031	0.068169	0.05	ILMN_3216	FMO6P	1
TBX5	44.18557	2.275638	0.05	ILMN_19141	TBX5	12
PCDHGB3	100.0759	5.16702	0.05	ILMN_7670	PCDHGB3	5
PLEKHA8	25.26671	1.306277	0.05	ILMN_12342	PLEKHA8	7
ARHGAP9	21.90195	1.135765	0.05	ILMN_22879	ARHGAP9	12
RAET1L	162.5496	8.452435	0.05	ILMN_162405	RAET1L	6
CCDC4	107.1883	5.581242	0.05	ILMN_1131	CCDC4	4
CACNA2D1	539.3322	28.09228	0.05	ILMN_14677	CACNA2D1	7
C10orf71	50.58533	2.636488	0.05	ILMN_15124	C10ORF71	10
MS4A12	70.83264	3.696033	0.05	ILMN_25117	MS4A12	11
CCL3L3	699.729	36.55357	0.05	ILMN_22857	CCL3L3	17
ANXA8L2	294.2123	15.45399	0.05	ILMN_2382	ANXA8L2	10
SYCP2L	613.9988	32.2688	0.05	ILMN_15370	SYCP2L	6
GPR21	107.9141	5.705681	0.05	ILMN_21871	GPR21	9
C12orf59	491.5259	26.0561	0.05	ILMN_13131	C12ORF59	12
LOC401233	825.7032	43.80123	0.05	ILMN_17894	LOC401233	6
MTNR1A	115.3051	6.116799	0.05	ILMN_9086	MTNR1A	4
ENPEP	458.9367	24.37319	0.05	ILMN_22750	ENPEP	4
OR4C13	272.7116	14.52645	0.05	ILMN_16650	OR4C13	11
C10orf26	904.3422	48.34442	0.05	ILMN_28562	C10ORF26	10
HLA-DQA1	49.60374	2.654403	0.05	ILMN_41173	HLA-DQA1	
EFS	14.23731	0.762614	0.05	ILMN_2968	EFS	14
OR14C36	315.8477	16.96087	0.05	ILMN_12674	OR14C36	1
NR2E1	365.4027	19.77126	0.05	ILMN_183120	NR2E1	6
FGF17	865.0561	46.82994	0.05	ILMN_9431	FGF17	8
HS3ST1	8865.453	480.4636	0.05	ILMN_18852	HS3ST1	4
PTPRH	267.0774	14.53561	0.05	ILMN_8628	PTPRH	19
TMSL8	5047.461	276.1436	0.05	ILMN_1117	TMSL8	X
MOXD1	181.5295	9.939241	0.05	ILMN_13883	MOXD1	6
CLEC4A	257.6152	14.24207	0.06	ILMN_8463	CLEC4A	12
NAT8	230.3858	12.85262	0.06	ILMN_28294	NAT8	2
PTGES	1038.021	58.242	0.06	ILMN_2778	PTGES	9
KCNS1	243.3115	13.69237	0.06	ILMN_14950	KCNS1	20
TRPC2	132.0572	7.506061	0.06	ILMN_14328	TRPC2	11
CYYR1	121.5783	6.917647	0.06	ILMN_5385	CYYR1	21
LMO1	17.97807	1.025986	0.06	ILMN_29972	LMO1	11
FBXL18	177.406	10.13389	0.06	ILMN_21876	FBXL18	

TGFB1	110.8133	6.339321	0.06	ILMN_29072	TGFB1	19
C10orf107	294.3596	16.84422	0.06	ILMN_28726	C10ORF107	10
CYP11B1	82.40302	4.73017	0.06	ILMN_25216	CYP11B1	8
FLJ32011	469.5235	27.19536	0.06	ILMN_2337	FLJ32011	1
TRHR	17.41915	1.01969	0.06	ILMN_174868	TRHR	8
AKAP12	1910.522	111.9041	0.06	ILMN_9320	AKAP12	6
FN1	12.78305	0.749711	0.06	ILMN_28846	FN1	2
SLC22A15	1001.989	58.80164	0.06	ILMN_26230	SLC22A15	1
VTN	58.1657	3.425587	0.06	ILMN_138128	VTN	17
MAP7D2	72.43414	4.276576	0.06	ILMN_6575	MAP7D2	X
CYP1A2	762.0991	45.08008	0.06	ILMN_19528	CYP1A2	15
REG3A	20.75305	1.228796	0.06	ILMN_21552	REG3A	2
KCNN2	7.67901	0.455478	0.06	ILMN_24100	KCNN2	5
OR10W1	16.66735	0.991297	0.06	ILMN_7227	OR10W1	11
DNASE1L3	117.2928	6.99615	0.06	ILMN_27679	DNASE1L3	3
LASS3	33.84087	2.019483	0.06	ILMN_9117	LASS3	15
GPR142	63.70926	3.805988	0.06	ILMN_20405	GPR142	17
HLA-DPA1	727.4617	43.48221	0.06	ILMN_13606	HLA-DPA1	6
TIAM1	384.2122	22.97075	0.06	ILMN_14448	TIAM1	21
TLR6	283.6547	16.9868	0.06	ILMN_15638	TLR6	4
C1QB	153.4395	9.193596	0.06	ILMN_138070	C1QB	1
KRTAP21-1	63.03168	3.802805	0.06	ILMN_8933	KRTAP21-1	21
SPATA19	50.95267	3.099308	0.06	ILMN_25885	SPATA19	11
PABPC1L2B	228.3635	13.90099	0.06	ILMN_181623	PABPC1L2B	X
ADAM32	83.01923	5.064464	0.06	ILMN_138164	ADAM32	8
CMAH	75.31087	4.601193	0.06	ILMN_13868	CMAH	6
RGS7	106.0836	6.490412	0.06	ILMN_7995	RGS7	
NTSR1	3584.121	219.4253	0.06	ILMN_13518	NTSR1	20
COX4I2	9.788609	0.60012	0.06	ILMN_2510	COX4I2	20
HHIPL1	27.43193	1.686233	0.06	ILMN_2441	HHIPL1	14
SEMA7A	261.1932	16.06721	0.06	ILMN_17564	SEMA7A	15
FASLG	39.88037	2.453672	0.06	ILMN_6949	FASLG	1
OR52B2	11.25685	0.693113	0.06	ILMN_20874	OR52B2	11
RLBP1L2	11.07061	0.684074	0.06	ILMN_3513	RLBP1L2	6
KLF8	678.7552	42.01764	0.06	ILMN_137639	KLF8	X
WFDC2	35.0844	2.179051	0.06	ILMN_9194	WFDC2	20
MGC40069	312.1228	19.40511	0.06	ILMN_30193	MGC40069	
TFAP2D	347.2097	21.59292	0.06	ILMN_9513	TFAP2D	6
PTPN14	884.7823	55.08277	0.06	ILMN_27079	PTPN14	1
SIGLEC10	561.0311	35.11571	0.06	ILMN_26163	SIGLEC10	19
OR51B4	1563.897	97.92303	0.06	ILMN_20729	OR51B4	11
SMO	112.0836	7.031411	0.06	ILMN_17921	SMO	7
ST6GALNAC	692.5899	43.53292	0.06	ILMN_30129	ST6GALNAC3	1
KIR3DL2	2666.587	167.9188	0.06	ILMN_29548	KIR3DL2	19
PDE11A	25.68575	1.620585	0.06	ILMN_1649	PDE11A	2
DOCK10	104.4947	6.600232	0.06	ILMN_15188	DOCK10	2
GYPE	250.3345	15.89617	0.06	ILMN_21264	GYPE	4

OR4K2	494.7169	32.01045	0.06	ILMN_165316	OR4K2	14
GSTA1	200.4661	12.98332	0.06	ILMN_30031	GSTA1	6
S100A3	753.6683	49.38156	0.07	ILMN_19000	S100A3	1
ZEB1	30.491	2.001451	0.07	ILMN_20482	ZEB1	10
LPAR1	2631.02	172.9179	0.07	ILMN_27567	LPAR1	9
SLC45A2	32.15189	2.130194	0.07	ILMN_16585	SLC45A2	5
LIMS3	89.49243	5.951618	0.07	ILMN_26870	LIMS3	2
OR4F21	619.5869	41.34108	0.07	ILMN_7961	OR4F21	8
NCAM2	38.53199	2.593184	0.07	ILMN_16738	NCAM2	21
PNPLA1	20.6402	1.391563	0.07	ILMN_20508	PNPLA1	6
C4orf18	646.4966	43.77322	0.07	ILMN_657	C4ORF18	4
SLCO2A1	35.21999	2.38494	0.07	ILMN_14229	SLCO2A1	3
PLCD4	341.6129	23.22283	0.07	ILMN_10729	PLCD4	2
SPANXA1	493.4296	33.67498	0.07	ILMN_9452	SPANXA1	X
LOC14995(	151.7247	10.38988	0.07	ILMN_26419	LOC149950	20
GZF1	502.4485	34.41224	0.07	ILMN_16614	GZF1	20
NLRP6	43.88678	3.01913	0.07	ILMN_26211	NLRP6	11
STK17A	2432.138	167.4541	0.07	ILMN_20263	STK17A	7
RAB40AL	672.898	46.37673	0.07	ILMN_24932	RAB40AL	X
SPTA1	176.7513	12.23791	0.07	ILMN_17651	SPTA1	1
CLEC12A	65.02663	4.522689	0.07	ILMN_24114	CLEC12A	12
KRT38	135.2825	9.411931	0.07	ILMN_2163	KRT38	17
FSIP1	553.8834	38.56038	0.07	ILMN_18959	FSIP1	15
ERV3	2249.495	156.626	0.07	ILMN_16354	ERV3	7
KCTD16	489.1974	34.15457	0.07	ILMN_21074	KCTD16	5
YY2	25.76829	1.800977	0.07	ILMN_27793	YY2	X
ZNF804B	928.4943	65.01265	0.07	ILMN_29627	ZNF804B	7
LOC65287(	61.51751	4.319426	0.07	ILMN_39015	LOC652870	
OR2T4	108.5139	7.628377	0.07	ILMN_24803	OR2T4	1
LAT2	575.6964	40.48395	0.07	ILMN_137018	LAT2	7
MS4A2	38.71852	2.725212	0.07	ILMN_18276	MS4A2	11
OR2A20P	485.2178	34.30021	0.07	ILMN_13267	OR2A20P	7
OR4F15	230.0657	16.34372	0.07	ILMN_24678	OR4F15	15
C19orf15	357.0809	25.44736	0.07	ILMN_22229	C19ORF15	19
PCDHA1	15.32434	1.094681	0.07	ILMN_15553	PCDHA1	5
OR7D4	11.02959	0.789956	0.07	ILMN_30101	OR7D4	19
LDOC1	84.13183	6.041683	0.07	ILMN_8953	LDOC1	X
C6	38.29685	2.753958	0.07	ILMN_26267	C6	5
CSF1R	458.1041	33.02472	0.07	ILMN_24980	CSF1R	5
EYA4	46.86086	3.387611	0.07	ILMN_24099	EYA4	6
ROS1	34.61374	2.503549	0.07	ILMN_8842	ROS1	6
GPSM3	127.9549	9.294691	0.07	ILMN_28125	GPSM3	6
LOC44034{	58.80164	4.274008	0.07	ILMN_12371	LOC440348	16
CSDC2	19.99838	1.454492	0.07	ILMN_23719	CSDC2	22
C10orf54	100.5716	7.331597	0.07	ILMN_174163	C10ORF54	10
CSTL1	36.96706	2.698685	0.07	ILMN_11818	CSTL1	20
MACROD2	162.6984	11.90693	0.07	ILMN_24564	MACROD2	20

FBF1	111.8745	8.211107	0.07	ILMN_179053	FBF1	17
SPATA12	688.7694	50.56573	0.07	ILMN_4789	SPATA12	3
ADCYAP1	305.3849	22.42911	0.07	ILMN_9995	ADCYAP1	18
FGFR2	126.177	9.269701	0.07	ILMN_20933	FGFR2	10
PIP5K2A	789.1995	58.02686	0.07	ILMN_6957	PIP5K2A	10
SIDT1	263.1947	19.42817	0.07	ILMN_24635	SIDT1	3
GLYAT	117.7298	8.729971	0.07	ILMN_3816	GLYAT	11
CP	34.62665	2.569239	0.07	ILMN_24449	CP	3
KRT17	1465.225	108.8569	0.07	ILMN_5566	KRT17	17
SERPINB9	1887.453	140.4892	0.07	ILMN_162722	SERPINB9	6
SPTBN5	549.1028	41.16832	0.07	ILMN_25344	SPTBN5	15
REP15	135.0389	10.13924	0.08	ILMN_26617	REP15	12
TCEB3B	47.99477	3.621564	0.08	ILMN_9597	TCEB3B	18
MYPN	139.836	10.60254	0.08	ILMN_27889	MYPN	10
GBP2	156.5418	11.88198	0.08	ILMN_13936	GBP2	1
OR4D10	190.405	14.49567	0.08	ILMN_183643	OR4D10	11
ARPM1	74.37653	5.67373	0.08	ILMN_8778	ARPM1	3
C10orf132	124.6494	9.518487	0.08	ILMN_4547	C10ORF132	10
ERAF	213.0014	16.27798	0.08	ILMN_10251	ERAF	16
FGF8	80.02519	6.123458	0.08	ILMN_19770	FGF8	10
ZNF391	441.7207	33.82647	0.08	ILMN_180888	ZNF391	6
SMPD1	102.695	7.888147	0.08	ILMN_10742	SMPD1	11
CYP4F22	315.9717	24.29504	0.08	ILMN_20313	CYP4F22	
CYP2E1	784.9677	60.36149	0.08	ILMN_27893	CYP2E1	10
HSPB8	364.7696	28.08331	0.08	ILMN_22076	HSPB8	12
C1S	232.3495	17.89011	0.08	ILMN_29321	C1S	12
GIMAP4	257.4694	19.84684	0.08	ILMN_26906	GIMAP4	7
INSM2	234.2607	18.05847	0.08	ILMN_3487	INSM2	14
DUSP5P	94.12557	7.283956	0.08	ILMN_39019	DUSP5P	1
CACNA1G	12.6901	0.986796	0.08	ILMN_11316	CACNA1G	17
IKZF1	69.42223	5.402069	0.08	ILMN_22185	IKZF1	7
OR6Y1	491.3625	38.29685	0.08	ILMN_29864	OR6Y1	1
ARL4D	4803.322	376.0399	0.08	ILMN_138313	ARL4D	17
COL9A2	1427.829	111.8745	0.08	ILMN_24828	COL9A2	1
LOC554235	44.00366	3.46412	0.08	ILMN_19242	LOC554235	19
ATXN1	2251.002	177.3624	0.08	ILMN_173593	ATXN1	6
IL18RAP	105.946	8.360571	0.08	ILMN_16084	IL18RAP	2
OR11H1	63.34686	5.000969	0.08	ILMN_178720	OR11H1	22
NKX2-2	29.7095	2.349592	0.08	ILMN_23320	NKX2-2	20
LOC440570	124.0575	9.814816	0.08	ILMN_19954	LOC440570	1
HMGA2	295.6564	23.50589	0.08	ILMN_11524	HMGA2	12
KCNJ14	126.7769	10.08474	0.08	ILMN_19428	KCNJ14	19
DKK1	6565.865	522.496	0.08	ILMN_22862	DKK1	10
IFI16	39.15724	3.120962	0.08	ILMN_2725	IFI16	1
HCLS1	117.371	9.362884	0.08	ILMN_10504	HCLS1	3
FLJ43763	233.8633	18.65955	0.08	ILMN_42480	FLJ43763	6
TH	50.52192	4.051291	0.08	ILMN_138010	TH	11

IL1F8	171.7629	13.77492	0.08	ILMN_4990	IL1F8	2
PRRX1	56.17031	4.513182	0.08	ILMN_9745	PRRX1	1
PRICKLE1	8550.005	687.1453	0.08	ILMN_15149	PRICKLE1	12
MRGPRD	2.529943	0.203408	0.08	ILMN_12978	MRGPRD	11
FLJ40113	396.4717	31.89981	0.08	ILMN_167867	FLJ40113	
KLF14	104.8137	8.460383	0.08	ILMN_1816	KLF14	7
CD48	113.435	9.162422	0.08	ILMN_12920	CD48	1
SYT1	2571.161	208.0149	0.08	ILMN_14093	SYT1	12
THBS4	200.9634	16.30048	0.08	ILMN_6151	THBS4	5
NME5	3991.914	324.7454	0.08	ILMN_13594	NME5	5
UNC13D	298.5761	24.39426	0.08	ILMN_25226	UNC13D	17
OR13F1	176.1141	14.44786	0.08	ILMN_9154	OR13F1	9
BCAN	19.3573	1.588412	0.08	ILMN_13064	BCAN	1
OR52B6	910.9514	74.76289	0.08	ILMN_3276	OR52B6	11
PTHLH	183.5878	15.06808	0.08	ILMN_4025	PTHLH	12
OR52A4	129.8317	10.66804	0.08	ILMN_13239	OR52A4	11
KCNAB1	106.7783	8.836827	0.08	ILMN_29046	KCNAB1	3
PTGER2	931.1142	77.07457	0.08	ILMN_6622	PTGER2	14
FOXP2	18.12732	1.505235	0.08	ILMN_424	FOXP2	7
MAPK4	288.5786	24.05363	0.08	ILMN_38629	MAPK4	18
EBF2	462.7606	38.71852	0.08	ILMN_177715	EBF2	8
TRIM48	101.8146	8.554153	0.08	ILMN_16488	TRIM48	11
OR6C6	21.98565	1.848047	0.08	ILMN_10806	OR6C6	12
HLA-DQB1	70.14678	5.922805	0.08	ILMN_29988	HLA-DQB1	6
KLHL35	217.1735	18.34601	0.08	ILMN_26322	KLHL35	11
SLC22A12	31.85727	2.69452	0.08	ILMN_8969	SLC22A12	11
DBX2	30.8291	2.610412	0.08	ILMN_9066	DBX2	12
CLEC1A	212.505	18.00824	0.08	ILMN_29762	CLEC1A	12
C4orf31	329.5344	27.94701	0.08	ILMN_7838	C4ORF31	4
FAM55A	617.4988	52.55174	0.09	ILMN_20615	FAM55A	11
CSF3	20.53539	1.751291	0.09	ILMN_20477	CSF3	17
GRIK3	22.58642	1.94351	0.09	ILMN_26224	GRIK3	1
KLF12	145.4749	12.58083	0.09	ILMN_26377	KLF12	13
C1orf215	1630.736	141.1077	0.09	ILMN_23368	C1ORF215	1
AP3B2	227.0467	19.66819	0.09	ILMN_8410	AP3B2	15
KCNC3	583.63	50.58533	0.09	ILMN_9225	KCNC3	19
PNMA3	51.79892	4.502741	0.09	ILMN_16752	PNMA3	X
STK33	436.891	37.98975	0.09	ILMN_6990	STK33	11
NAIP	75.94329	6.622828	0.09	ILMN_174418	NAIP	5
OLFM1	585.3767	51.09022	0.09	ILMN_1435	OLFM1	9
KCND1	53.32381	4.654612	0.09	ILMN_4763	KCND1	X
LOC374491	786.5261	68.70766	0.09	ILMN_137465	LOC374491	13
ZSCAN4	57.73266	5.064295	0.09	ILMN_21186	ZSCAN4	19
CLIC2	382.7893	33.59614	0.09	ILMN_16456	CLIC2	X
PYHIN1	159.6393	14.01933	0.09	ILMN_729	PYHIN1	1
DPPA4	196.0383	17.21808	0.09	ILMN_12242	DPPA4	3
FLJ44635	81.15359	7.136241	0.09	ILMN_27866	FLJ44635	X

OR10A7	46.87914	4.126487	0.09	ILMN_3277	OR10A7	12
OR6C3	530.5732	46.7906	0.09	ILMN_4043	OR6C3	12
1-Mar	108.17	9.567387	0.09	ILMN_30212	1-Mar	4
C10orf30	175.4359	15.66633	0.09	ILMN_21078	C10ORF30	10
PTPRZ1	197.2236	17.64202	0.09	ILMN_3168	PTPRZ1	7
OR2AG2	54.27122	4.858544	0.09	ILMN_12673	OR2AG2	11
HOMER2	279.2808	25.04314	0.09	ILMN_2937	HOMER2	15
SPATA4	1837.454	165.2121	0.09	ILMN_22568	SPATA4	4
YJEFN3	242.0309	21.77047	0.09	ILMN_3913	YJEFN3	19
MSN	3203.674	288.5786	0.09	ILMN_10863	MSN	X
C9orf96	225.8715	20.4215	0.09	ILMN_26374	C9ORF96	9
SV2A	147.7352	13.35705	0.09	ILMN_14178	SV2A	1
OR8D2	193.6046	17.50833	0.09	ILMN_10194	OR8D2	11
PITPNM1	385.0062	34.8231	0.09	ILMN_18155	PITPNM1	11
SMURF2	154.7262	14.01928	0.09	ILMN_5217	SMURF2	17
CAPS2	677.1155	61.54478	0.09	ILMN_2288	CAPS2	12
ST8SIA1	3.34917	0.305764	0.09	ILMN_181880	ST8SIA1	12
LETM2	489.0198	44.80922	0.09	ILMN_4457	LETM2	8
ZBTB38	235.1831	21.55773	0.09	ILMN_169611	ZBTB38	
OR4F3	1078.407	98.86201	0.09	ILMN_15015	OR4F3	5
RLN1	173.0857	15.90921	0.09	ILMN_28164	RLN1	9
FCGR1A	124.2763	11.50018	0.09	ILMN_2377	FCGR1A	1
CKM	190.2437	17.66892	0.09	ILMN_29008	CKM	19
TMEM39A	821.7483	76.39596	0.09	ILMN_5837	TMEM39A	3
MMP1	1662.035	154.5339	0.09	ILMN_26214	MMP1	11
BZRPL1	55.04656	5.119438	0.09	ILMN_30282	BZRPL1	6
BEX4	67.16034	6.247946	0.09	ILMN_171171	BEX4	X
CLDN10	17.35998	1.617369	0.09	ILMN_1784	CLDN10	13
MTMR7	107.4985	10.02892	0.09	ILMN_23906	MTMR7	8
PLEK	44.74876	4.195179	0.09	ILMN_5756	PLEK	2
RPLP0P2	134.4664	12.60877	0.09	ILMN_11391	RPLP0P2	11
NXPH2	172.7112	16.22473	0.09	ILMN_11806	NXPH2	2
ECM1	41.03705	3.857135	0.09	ILMN_6103	ECM1	1
KCNA3	1110.409	104.4233	0.09	ILMN_138735	KCNA3	1
CD8B	124.6093	11.72547	0.09	ILMN_137981	CD8B	2
OR52K1	810.8876	76.32079	0.09	ILMN_4622	OR52K1	11
OR4F4	637.3654	60.05074	0.09	ILMN_28355	OR4F4	15
C13orf28	34.78599	3.279213	0.09	ILMN_27632	C13ORF28	13
C17orf91	2555.032	240.9496	0.09	ILMN_17772	C17ORF91	17
SYT9	61.64067	5.813304	0.09	ILMN_22548	SYT9	11
INSC	136.8193	12.92367	0.09	ILMN_27260	INSC	11
TMEM136	1352.22	128.3749	0.09	ILMN_16309	TMEM136	11
CCDC33	46.63885	4.440491	0.1	ILMN_7534	CCDC33	15
MGC27121	763.3498	72.79701	0.1	ILMN_29646	MGC27121	5
FSD1CL	1105.825	105.6166	0.1	ILMN_136950	FSD1CL	9
COLQ	120.3726	11.52536	0.1	ILMN_14798	COLQ	
C21orf49	114.8831	11.12029	0.1	ILMN_1248	C21ORF49	21

KRT16	1209.771	117.4225	0.1	ILMN_23524	KRT16	17
OR6C65	100.734	9.78062	0.1	ILMN_8566	OR6C65	12
SRPK3	186.6659	18.14084	0.1	ILMN_4402	SRPK3	X
GPR19	93.09293	9.054008	0.1	ILMN_20675	GPR19	12
CCDC73	82.83814	8.065422	0.1	ILMN_137085	CCDC73	11
UBL4B	528.9174	51.55862	0.1	ILMN_15157	UBL4B	1
WWTR1	2107.482	205.6951	0.1	ILMN_24248	WWTR1	3
KRTAP8-1	14.64034	1.437599	0.1	ILMN_6079	KRTAP8-1	21
MLLT11	5611.708	552.0785	0.1	ILMN_21397	MLLT11	1
UHRF1BP1	4048.802	399.5074	0.1	ILMN_24682	UHRF1BP1L	12
TAGLN3	61.06792	6.036184	0.1	ILMN_7302	TAGLN3	3
S100A2	1232.374	122.5804	0.1	ILMN_4973	S100A2	1
NPBWR2	169.707	16.88784	0.1	ILMN_7400	NPBWR2	20
ACCN4	23.87202	2.377438	0.1	ILMN_10550	ACCN4	2
MFNG	447.0307	44.54054	0.1	ILMN_25964	MFNG	22
WDR40B	20.64294	2.077666	0.1	ILMN_4734	WDR40B	X
APLNR	73.84357	7.485024	0.1	ILMN_139058	APLNR	11
PLA2G4D	224.8036	22.82235	0.1	ILMN_4570	PLA2G4D	15
PLAC8L1	924.2464	94.09278	0.1	ILMN_5526	PLAC8L1	5
MASP1	28.96254	2.951027	0.1	ILMN_9215	MASP1	3
ARHGAP29	528.6337	53.99101	0.1	ILMN_29935	ARHGAP29	1
TBC1D9	3070.035	314.3813	0.1	ILMN_25527	TBC1D9	4
TULP1	58.67392	6.016198	0.1	ILMN_20486	TULP1	6
TEDDM1	233.1102	23.95785	0.1	ILMN_139205	TEDDM1	1
CRYGS	182.2697	18.75518	0.1	ILMN_11890	CRYGS	3
MAOB	723.8688	74.63008	0.1	ILMN_9884	MAOB	X
LOC348262	1020.001	105.3334	0.1	ILMN_643	LOC348262	17|NT_113944.1
TMEM30B	15.63263	1.614942	0.1	ILMN_3369	TMEM30B	14
MFSD7	13.05929	1.351626	0.1	ILMN_10818	MFSD7	
EHD2	551.8898	57.41237	0.1	ILMN_14594	EHD2	19
COL11A2	16.90356	1.761522	0.1	ILMN_19749	COL11A2	6
PANX3	162.3061	16.99335	0.1	ILMN_5014	PANX3	11
PKD1L3	82.87585	8.710011	0.11	ILMN_1895	PKD1L3	
DCST2	273.9058	28.79193	0.11	ILMN_19963	DCST2	1
MAFB	179.9651	18.92344	0.11	ILMN_3663	MAFB	20
KIAA0773	86.94006	9.170612	0.11	ILMN_20985	KIAA0773	7
C9orf167	598.2415	63.22697	0.11	ILMN_6900	C9ORF167	9
PDGFB	448.2733	47.47948	0.11	ILMN_30109	PDGFB	22
LOC92017	399.5074	42.40837	0.11	ILMN_138578	LOC92017	16
ZNF302	1426.023	151.4785	0.11	ILMN_26556	ZNF302	19
MAB21L2	130.6472	13.92926	0.11	ILMN_4152	MAB21L2	4
HBD	137.1079	14.65398	0.11	ILMN_9543	HBD	11
OR6B1	168.1928	18.01805	0.11	ILMN_27881	OR6B1	7
OR2G6	50.90374	5.467768	0.11	ILMN_10457	OR2G6	1
ZNF826	81.79356	8.792533	0.11	ILMN_41742	ZNF826	19
FANCF	1217.026	131.1722	0.11	ILMN_2711	FANCF	11
LHFPL2	2223.147	240.3264	0.11	ILMN_26106	LHFPL2	5

GSTM3	109.4335	11.83151	0.11	ILMN_2804	GSTM3	1
PGLYRP3	81.34659	8.831076	0.11	ILMN_3811	PGLYRP3	1
CCDC85A	205.9242	22.39512	0.11	ILMN_171289	CCDC85A	2
GALNTL2	64.97927	7.069797	0.11	ILMN_26539	GALNTL2	3
APOA4	17.11585	1.862409	0.11	ILMN_13361	APOA4	11
SLC26A4	453.0615	49.32947	0.11	ILMN_23896	SLC26A4	7
SEZ6L	695.3132	75.76089	0.11	ILMN_27742	SEZ6L	22
OR4M1	14.24926	1.5588	0.11	ILMN_179466	OR4M1	14
MAGI1	162.7849	17.87863	0.11	ILMN_14103	MAGI1	3
DPCR1	165.5567	18.20026	0.11	ILMN_19914	DPCR1	6
MTNR1B	24.20478	2.660939	0.11	ILMN_27810	MTNR1B	11
CD1C	62.52827	6.88894	0.11	ILMN_25152	CD1C	1
SH3GL3	3324.239	366.8846	0.11	ILMN_17051	SH3GL3	15
C10orf68	1413.024	156.3554	0.11	ILMN_21881	C10ORF68	10
CD79B	286.7777	31.77598	0.11	ILMN_37874	CD79B	17
OR2M2	8.300617	0.920543	0.11	ILMN_23374	OR2M2	1
DLK1	59.95914	6.650681	0.11	ILMN_13065	DLK1	14
OR5M9	88.91391	9.864005	0.11	ILMN_2095	OR5M9	11
COL2A1	88.44526	9.812661	0.11	ILMN_8700	COL2A1	12
ABCD4	492.7944	54.76084	0.11	ILMN_177951	ABCD4	14
C9orf97	1027.523	114.3191	0.11	ILMN_27224	C9ORF97	9
CCDC81	146.8823	16.38899	0.11	ILMN_7066	CCDC81	11
KRT86	675.0219	75.33832	0.11	ILMN_2465	KRT86	12
HS3ST3B1	112.799	12.61943	0.11	ILMN_21193	HS3ST3B1	17
FGF10	70.65556	7.908638	0.11	ILMN_19131	FGF10	5
NEFM	5.060614	0.567009	0.11	ILMN_1804	NEFM	8
LOC65029[illegible]	368.457	41.32722	0.11	ILMN_175055	LOC650293	
KIAA1409	77.21966	8.666483	0.11	ILMN_42698	KIAA1409	14
DPPA5	71.29955	8.00634	0.11	ILMN_3979	DPPA5	6
FMO2	272.1658	30.59659	0.11	ILMN_4858	FMO2	1
FABP3	187.1471	21.06141	0.11	ILMN_19284	FABP3	1
ODF4	89.58646	10.08721	0.11	ILMN_4357	ODF4	17
ABCA4	645.7463	72.85706	0.11	ILMN_28625	ABCA4	1
PADI1	5.488928	0.620682	0.11	ILMN_1296	PADI1	1
IGFL3	460.7924	52.21541	0.11	ILMN_17801	IGFL3	19
TMEM16A	2894.126	328.4337	0.11	ILMN_25243	TMEM16A	11
SFTPB	79.52881	9.04731	0.11	ILMN_6947	SFTPB	2
P2RX7	444.8974	50.64915	0.11	ILMN_26570	P2RX7	12
PDGFD	13.07625	1.491385	0.11	ILMN_138561	PDGFD	11
KRT84	42.00079	4.80029	0.11	ILMN_138585	KRT84	12
MALAT1	558.1865	63.80814	0.11	ILMN_16418	MALAT1	11
OR5I1	197.7267	22.62212	0.11	ILMN_24630	OR5I1	11
OR4F5	528.2695	60.49332	0.11	ILMN_178117	OR4F5	1
OR2L2	156.7388	17.98424	0.11	ILMN_138871	OR2L2	1
SPRYD5	389.7856	44.74876	0.11	ILMN_14013	SPRYD5	11
FBXW10	245.0752	28.18209	0.11	ILMN_18868	FBXW10	17
FCRL3	102.4551	11.79774	0.12	ILMN_7307	FCRL3	1

IGSF10	145.4802	16.77877	0.12	ILMN_15444	IGSF10	3
LOC339240	11.82884	1.368051	0.12	ILMN_5302	LOC339240	17
RUNDC2A	187.8393	21.78119	0.12	ILMN_705	RUNDC2A	16
PAX5	61.54478	7.13902	0.12	ILMN_899	PAX5	9
C12orf26	895.5394	104.0044	0.12	ILMN_732	C12ORF26	12
OR4C11	155.0074	18.09389	0.12	ILMN_30340	OR4C11	11
HFE2	56.42329	6.591552	0.12	ILMN_25077	HFE2	1
GLT25D2	23.11565	2.701246	0.12	ILMN_29386	GLT25D2	1
OR10H1	64.58644	7.554374	0.12	ILMN_19170	OR10H1	19
ZBTB46	564.8785	66.07188	0.12	ILMN_12382	ZBTB46	20
SSX2	102.5162	12.00832	0.12	ILMN_163873	SSX2	X
LOC388199	4.403302	0.516746	0.12	ILMN_18862	LOC388199	16
RESP18	20.41396	2.400296	0.12	ILMN_27080	RESP18	
SCUBE3	171.3403	20.15855	0.12	ILMN_844	SCUBE3	6
OR10Q1	157.5634	18.53963	0.12	ILMN_4400	OR10Q1	11
GFRAL	76.75224	9.070415	0.12	ILMN_8701	GFRAL	6
CDH8	66.97987	7.926903	0.12	ILMN_10366	CDH8	16
RGS4	117.5914	13.93365	0.12	ILMN_15378	RGS4	1
SERPINE1	156.87	18.61446	0.12	ILMN_6244	SERPINE1	7
PCDHA12	220.7037	26.30689	0.12	ILMN_19729	PCDHA12	5
OR13A1	84.64824	10.09688	0.12	ILMN_16980	OR13A1	10
ZNF771	256.2465	30.60404	0.12	ILMN_4259	ZNF771	16
GRIN3A	120.2779	14.37556	0.12	ILMN_15477	GRIN3A	9
LEF1	70.62707	8.445633	0.12	ILMN_30265	LEF1	4
FLJ35776	117.4814	14.07429	0.12	ILMN_137726	FLJ35776	18
ZBTB8	369.7076	44.3682	0.12	ILMN_165052	ZBTB8	1
ZBTB20	1773.833	213.3	0.12	ILMN_27339	ZBTB20	3
DYSF	186.0779	22.4777	0.12	ILMN_25101	DYSF	2
PLA2G4C	600.0824	72.63516	0.12	ILMN_29328	PLA2G4C	19
C2orf61	64.82134	7.863967	0.12	ILMN_505	C2ORF61	2
OR2B11	281.241	34.18343	0.12	ILMN_21348	OR2B11	1
MRGPRF	77.86314	9.473304	0.12	ILMN_517	MRGPRF	11
C17orf77	203.8861	24.84993	0.12	ILMN_19244	C17ORF77	17
FAM176B	2224.511	271.4727	0.12	ILMN_3804	FAM176B	1
GAP43	588.9638	72.0666	0.12	ILMN_28511	GAP43	3
ERG	73.4492	8.99474	0.12	ILMN_5934	ERG	21
TG	96.57072	11.85246	0.12	ILMN_15960	TG	8
DMBX1	101.4317	12.45736	0.12	ILMN_13566	DMBX1	1
REM1	102.9677	12.65959	0.12	ILMN_5270	REM1	20
CABLES2	1512.35	186.28	0.12	ILMN_28000	CABLES2	20
HSP90AB4P	12.5382	1.545457	0.12	ILMN_138912	HSP90AB4P	15
BACE1	547.877	67.54914	0.12	ILMN_12354	BACE1	11
DEFB106B	38.40108	4.734814	0.12	ILMN_165994	DEFB106B	8
DYDC2	415.9458	51.3705	0.12	ILMN_21406	DYDC2	10
PHYHIPL	110.874	13.71822	0.12	ILMN_22045	PHYHIPL	10
NBEA	605.911	75.0218	0.12	ILMN_171545	NBEA	13
TNNC2	1051.505	130.2027	0.12	ILMN_25741	TNNC2	20

HEXIM1	64.90768	8.057402	0.12	ILMN_20820	HEXIM1	17
IL29	126.3811	15.70708	0.12	ILMN_12594	IL29	19
NOL4	15.16145	1.889991	0.12	ILMN_16134	NOL4	18
CNTF	243.8537	30.44615	0.12	ILMN_5425	CNTF	11
SLC6A14	77.33706	9.672173	0.13	ILMN_27148	SLC6A14	X
TMEM16B	91.62877	11.46258	0.13	ILMN_13011	TMEM16B	12
LOC441282	16.32821	2.044037	0.13	ILMN_163029	LOC441282	7
PAG1	606.4855	76.05444	0.13	ILMN_174074	PAG1	8
WNT16	153.0822	19.22465	0.13	ILMN_12695	WNT16	7
IL11	250.3614	31.48583	0.13	ILMN_23651	IL11	19
TMEM45A	2673.666	336.3907	0.13	ILMN_30168	TMEM45A	3
MRO	84.88142	10.6864	0.13	ILMN_20206	MRO	18
IL11RA	1190.4	150.3001	0.13	ILMN_3536	IL11RA	9
C10orf10	74.59578	9.419544	0.13	ILMN_8623	C10ORF10	10
C20orf75	123.728	15.62641	0.13	ILMN_21430	C20ORF75	20
KCND3	32.33976	4.08542	0.13	ILMN_29332	KCND3	1
C6orf138	188.5963	23.86889	0.13	ILMN_17433	C6ORF138	6
CLEC2B	37.0767	4.69457	0.13	ILMN_6545	CLEC2B	12
ADRB3	42.4538	5.38427	0.13	ILMN_1876	ADRB3	8
FCRLA	15.35946	1.952856	0.13	ILMN_2598	FCRLA	1
PLA2G2E	12.72515	1.617963	0.13	ILMN_16206	PLA2G2E	1
TRIM58	43.36845	5.514394	0.13	ILMN_3232	TRIM58	1
C20orf106	529.2239	67.2944	0.13	ILMN_8448	C20ORF106	20
FCAMR	118.7256	15.10319	0.13	ILMN_180539	FCAMR	
APOBEC3F	1009.228	128.4013	0.13	ILMN_18531	APOBEC3F	22
CST5	43.59688	5.550448	0.13	ILMN_10970	CST5	20
ATP11A	148.5393	18.91227	0.13	ILMN_13005	ATP11A	13
CD300LG	59.40127	7.565604	0.13	ILMN_20387	CD300LG	17
SERPINB2	20.0889	2.56175	0.13	ILMN_14466	SERPINB2	18
ARHGAP25	9.947389	1.268864	0.13	ILMN_14823	ARHGAP25	2
GJA1	491.1279	62.69331	0.13	ILMN_5665	GJA1	6
CAPN11	174.7195	22.30983	0.13	ILMN_2790	CAPN11	6
TRIM46	55.92145	7.143486	0.13	ILMN_18492	TRIM46	1
UGT3A1	129.067	16.4898	0.13	ILMN_2396	UGT3A1	5
VMD2L3	17.73174	2.266872	0.13	ILMN_7183	VMD2L3	12
MAL	211.8653	27.14228	0.13	ILMN_3411	MAL	2
C19orf39	97.58952	12.52731	0.13	ILMN_10203	C19ORF39	19
C1RL	408.3844	52.43829	0.13	ILMN_13968	C1RL	12
OR8G1	21.58573	2.771738	0.13	ILMN_15827	OR8G1	11
OR5T2	80.28081	10.30903	0.13	ILMN_169850	OR5T2	11
NKIRAS1	1400.465	180.4558	0.13	ILMN_28229	NKIRAS1	3
ZNF560	64.09987	8.280179	0.13	ILMN_29708	ZNF560	19
OR1N2	105.7275	13.66623	0.13	ILMN_20770	OR1N2	9
SLC35B4	878.5901	113.7116	0.13	ILMN_19120	SLC35B4	7
C3orf44	93.92325	12.16971	0.13	ILMN_138660	C3ORF44	3
LOXL4	4682.043	607.384	0.13	ILMN_9214	LOXL4	10
SLC24A2	523.0357	67.90998	0.13	ILMN_2590	SLC24A2	9

DBP	1114.255	144.8035	0.13	ILMN_1724	DBP	19
LOC284912	188.7925	24.57116	0.13	ILMN_28431	LOC284912	22
NTSR2	9.447908	1.234075	0.13	ILMN_6480	NTSR2	2
FOXI2	38.3159	5.009554	0.13	ILMN_5893	FOXI2	10
MMRN1	510.3016	66.73386	0.13	ILMN_3526	MMRN1	4
GPM6A	20.51239	2.684542	0.13	ILMN_19818	GPM6A	4
ALDH1A3	4010.051	526.1594	0.13	ILMN_25111	ALDH1A3	15
TRIM9	849.8455	111.7627	0.13	ILMN_11039	TRIM9	14
CLEC2D	88.67917	11.67884	0.13	ILMN_25661	CLEC2D	12
PPP1R9A	1779.941	234.4395	0.13	ILMN_36947	PPP1R9A	7
ABCA12	136.5121	17.99988	0.13	ILMN_7547	ABCA12	2
ELOVL2	73.37148	9.675968	0.13	ILMN_27477	ELOVL2	6
UBD	39.06705	5.171221	0.13	ILMN_20133	UBD	6
RRAGD	914.8077	121.3248	0.13	ILMN_5663	RRAGD	6
SLC6A16	192.5041	25.53177	0.13	ILMN_10688	SLC6A16	19
ANK2	586.9165	77.91884	0.13	ILMN_12447	ANK2	4
HLA-DRB1	198.3667	26.34333	0.13	ILMN_20550	HLA-DRB1	6
ARG1	94.15685	12.54057	0.13	ILMN_19494	ARG1	6
SLC6A11	455.8502	60.86195	0.13	ILMN_24011	SLC6A11	3
C9orf169	1299.725	173.7128	0.13	ILMN_13120	C9ORF169	
ITPR1	2302.984	308.5461	0.13	ILMN_12245	ITPR1	3
HTR7	66.93854	8.969147	0.13	ILMN_8849	HTR7	10
PRAF2	231.5147	31.03933	0.13	ILMN_29780	PRAF2	X
FAM18B2	58.30004	7.819204	0.13	ILMN_10298	FAM18B2	17
CSPG4	590.9113	79.30994	0.13	ILMN_1596	CSPG4	15
CST8	16.4898	2.222269	0.13	ILMN_7399	CST8	20
GUCA2B	9.362884	1.26217	0.13	ILMN_3169	GUCA2B	1
LOC728358	321.0394	43.45056	0.14	ILMN_176067	LOC728358	8
TAS2R60	20.20406	2.744766	0.14	ILMN_21776	TAS2R60	7
HEMGN	51.24594	6.965651	0.14	ILMN_10958	HEMGN	9
FBXO15	1202.237	163.4985	0.14	ILMN_18560	FBXO15	18
FLJ12331	198.9689	27.0909	0.14	ILMN_29944	FLJ12331	16
EMR4	115.8037	15.77597	0.14	ILMN_182626	EMR4	
LGI2	8.56742	1.167187	0.14	ILMN_24987	LGI2	4
POU4F1	44.82262	6.10756	0.14	ILMN_8123	POU4F1	13
GPRC6A	115.741	15.79495	0.14	ILMN_28390	GPRC6A	6
SLC38A6	403.9699	55.36704	0.14	ILMN_7226	SLC38A6	14
OR4X2	88.53217	12.13489	0.14	ILMN_18857	OR4X2	11
SOX30	66.49867	9.115011	0.14	ILMN_12390	SOX30	5
DZIP3	4.759926	0.656585	0.14	ILMN_19622	DZIP3	3
SCN3B	104.7936	14.4713	0.14	ILMN_13375	SCN3B	11
LOC441178	42.51314	5.873197	0.14	ILMN_25862	LOC441178	6
AMDHD1	2142.196	296.9915	0.14	ILMN_26806	AMDHD1	12
KLK5	207.1059	28.75979	0.14	ILMN_30002	KLK5	19
PDE7B	415.4562	58.41114	0.14	ILMN_975	PDE7B	6
GP2	17.85957	2.511127	0.14	ILMN_8624	GP2	16
PRAMEF7	411.1364	57.97893	0.14	ILMN_172566	PRAMEF7	1

FAM90A1	299.8424	42.32537	0.14	ILMN_16557	FAM90A1	12
MMP24	331.0173	46.75851	0.14	ILMN_16467	MMP24	20
MYT1	1439.207	203.6872	0.14	ILMN_3667	MYT1	20
DCDC2	197.1633	27.98776	0.14	ILMN_139202	DCDC2	6
SMCR8	379.67	53.91426	0.14	ILMN_30054	SMCR8	17
MMP25	32.7709	4.653679	0.14	ILMN_10476	MMP25	16
CXorf58	137.4137	19.52213	0.14	ILMN_6231	CXORF58	X
PRR18	11.68116	1.663439	0.14	ILMN_4896	PRR18	6
LOC375323	17.75201	2.533068	0.14	ILMN_29175	LOC375323	3
LIX1L	439.5437	62.72497	0.14	ILMN_3572	LIX1L	1
SYT4	143.9267	20.57739	0.14	ILMN_21875	SYT4	18
STOML3	364.4854	52.23506	0.14	ILMN_27842	STOML3	13
ZNF117	273.8017	39.31586	0.14	ILMN_11565	ZNF117	7
NEUROD4	173.3002	24.91986	0.14	ILMN_12627	NEUROD4	12
DEFB125	262.9583	37.85173	0.14	ILMN_28411	DEFB125	20
CACNA1F	11.68615	1.683259	0.14	ILMN_29802	CACNA1F	X
LIN7A	81.63275	11.78951	0.14	ILMN_24070	LIN7A	12
TSC22D3	105.7598	15.28602	0.14	ILMN_9893	TSC22D3	X
CDH12	232.0032	33.56913	0.14	ILMN_23099	CDH12	5
BEST3	268.6004	38.9208	0.14	ILMN_7183	BEST3	12
OR51F2	124.6851	18.11756	0.15	ILMN_173668	OR51F2	11
TUBA8	363.2381	52.80371	0.15	ILMN_11580	TUBA8	22
CTCFL	504.975	73.47086	0.15	ILMN_18321	CTCFL	20
STH	203.6872	29.66187	0.15	ILMN_1785	STH	17
CORO2B	320.7257	47.01363	0.15	ILMN_22097	CORO2B	15
CRISP2	21.39659	3.137826	0.15	ILMN_29809	CRISP2	6
CPN2	179.0351	26.27717	0.15	ILMN_42585	CPN2	
TCL6	60.33151	8.859769	0.15	ILMN_138253	TCL6	14
AMY2B	332.1207	48.82681	0.15	ILMN_5982	AMY2B	1
IL8	5200.165	765.5746	0.15	ILMN_179575	IL8	4
SERPINA11	67.19621	9.911169	0.15	ILMN_42900	SERPINA11	14
GLRA3	3.350633	0.494324	0.15	ILMN_165159	GLRA3	4
PKHD1	108.3315	15.98349	0.15	ILMN_9778	PKHD1	6
UGT1A7	42.15029	6.222208	0.15	ILMN_2145	UGT1A7	2
NOMO2	430.7907	63.67776	0.15	ILMN_7034	NOMO2	16
LOC729399	195.8443	28.95451	0.15	ILMN_15798	LOC729399	2
RHOBTB3	208.496	30.84694	0.15	ILMN_10998	RHOBTB3	5
SIGLEC1	24.73729	3.662609	0.15	ILMN_137671	SIGLEC1	20
ABCA5	282.22	41.81948	0.15	ILMN_8430	ABCA5	17
GREB1	119.4054	17.71987	0.15	ILMN_19956	GREB1	2
FXYD6	414.4052	61.4985	0.15	ILMN_26508	FXYD6	11
psiTPTE22	150.4481	22.33834	0.15	ILMN_12454	PSITPTE22	22
OR8S1	32.28822	4.796613	0.15	ILMN_7050	OR8S1	12
C2orf78	542.0447	80.54018	0.15	ILMN_174703	C2ORF78	2
ALPK2	371.4514	55.19764	0.15	ILMN_30352	ALPK2	18
PRDM7	2104.452	313.0699	0.15	ILMN_5534	PRDM7	16
ASGR2	55.61996	8.284132	0.15	ILMN_18166	ASGR2	17

PKIB	1335.686	199.3503	0.15	ILMN_27917	PKIB	6
EFHC1	451.9737	67.55805	0.15	ILMN_14235	EFHC1	
RNF2	608.6708	91.00764	0.15	ILMN_7319	RNF2	1
ITIH5L	72.9882	10.91591	0.15	ILMN_23026	ITIH5L	X
ARSG	902.4713	135.1017	0.15	ILMN_769	ARSG	17
SLC1A3	163.5812	24.49816	0.15	ILMN_17250	SLC1A3	5
TEX28	68.004	10.26071	0.15	ILMN_14309	TEX28	X
DRD1	58.50344	8.859972	0.15	ILMN_139409	DRD1	5
SCARA5	247.7243	37.5419	0.15	ILMN_15503	SCARA5	8
OR5L1	114.1814	17.32933	0.15	ILMN_178453	OR5L1	11
PGK2	19.68003	2.993138	0.15	ILMN_173148	PGK2	6
MYCT1	79.80662	12.21511	0.15	ILMN_12226	MYCT1	6
CRYGA	37.23074	5.701821	0.15	ILMN_26629	CRYGA	2
SLC17A3	9.662539	1.480318	0.15	ILMN_29077	SLC17A3	6
WDR88	13.37486	2.052263	0.15	ILMN_8223	WDR88	19
RP1L1	472.5672	72.60048	0.15	ILMN_5781	RP1L1	
ITGBL1	26.5774	4.083361	0.15	ILMN_22983	ITGBL1	13
ZNF285A	67.70483	10.41744	0.15	ILMN_2983	ZNF285A	19
OR51V1	131.688	20.33814	0.15	ILMN_26860	OR51V1	11
BANK1	1125.241	173.8498	0.15	ILMN_25922	BANK1	4
CCDC11	442.8258	68.47589	0.15	ILMN_14245	CCDC11	18
C16orf62	279.7671	43.27054	0.15	ILMN_13238	C16ORF62	16
SLC17A5	497.0794	76.93101	0.15	ILMN_2681	SLC17A5	6
LYST	501.0565	77.55678	0.15	ILMN_12138	LYST	1
C4orf26	197.3724	30.57403	0.15	ILMN_4729	C4ORF26	4
SLC47A2	22.80967	3.544129	0.16	ILMN_18818	SLC47A2	17
INA	131.056	20.37388	0.16	ILMN_9766	INA	10
WFDC5	104.5647	16.31068	0.16	ILMN_3350	WFDC5	20
HERC2P4	79.0668	12.33812	0.16	ILMN_137553	HERC2P4	16
BARHL2	34.39053	5.37132	0.16	ILMN_575	BARHL2	1
C3orf42	694.6616	108.5235	0.16	ILMN_137275	C3ORF42	3
LOC158381	64.22119	10.03387	0.16	ILMN_3295	LOC158381	9
ESR2	57.86019	9.091072	0.16	ILMN_167906	ESR2	14
DEFB118	1150.594	180.8566	0.16	ILMN_23261	DEFB118	20
MN1	2237.926	351.8745	0.16	ILMN_177685	MN1	22
LILRA6	791.8935	124.6494	0.16	ILMN_27203	LILRA6	19
KIAA1529	12.5918	1.985324	0.16	ILMN_8535	KIAA1529	9
C9orf68	78.18463	12.33129	0.16	ILMN_27658	C9ORF68	9
DNAJC12	163.7065	25.82598	0.16	ILMN_18576	DNAJC12	10
KIAA0853	80.24834	12.68768	0.16	ILMN_8304	KIAA0853	13
LOC285033	189.3404	29.99185	0.16	ILMN_1148	LOC285033	2
OLFML1	89.41628	14.17618	0.16	ILMN_1368	OLFML1	11
RNF113B	97.3929	15.45981	0.16	ILMN_20956	RNF113B	13
SLC6A15	7.289727	1.159266	0.16	ILMN_13282	SLC6A15	12
SHOX2	208.4134	33.25545	0.16	ILMN_137441	SHOX2	3
ARTN	190.6408	30.45788	0.16	ILMN_7178	ARTN	1
ETV3L	457.8717	73.17371	0.16	ILMN_28469	ETV3L	1

DTX1	106.8108	17.1726	0.16	ILMN_23758	DTX1	12
CRLF2	32.35902	5.213697	0.16	ILMN_18609	CRLF2	Y
ZNF512B	579.2337	93.45293	0.16	ILMN_1216	ZNF512B	20
ST6GALNA(	2326.699	375.5479	0.16	ILMN_12045	ST6GALNAC5	1
DUSP8	693.9426	112.1309	0.16	ILMN_21272	DUSP8	11
WDR66	84.84411	13.71234	0.16	ILMN_4287	WDR66	12
SYNJ2	896.1596	144.8787	0.16	ILMN_8732	SYNJ2	6
GGTLC1	70.3344	11.37633	0.16	ILMN_17297	GGTLC1	20
DNAH8	119.078	19.29358	0.16	ILMN_18322	DNAH8	6
PHTF1	967.6266	156.7915	0.16	ILMN_25225	PHTF1	1
PPP2R2C	1100.559	178.4124	0.16	ILMN_15268	PPP2R2C	4
CD86	44.36286	7.20137	0.16	ILMN_23756	CD86	3
FLJ45445	161.0024	26.17711	0.16	ILMN_163390	FLJ45445	
C11orf47	882.7679	143.6291	0.16	ILMN_171169	C11ORF47	11
OPA3	147.3733	24.02489	0.16	ILMN_11296	OPA3	19
MEI1	111.3287	18.21334	0.16	ILMN_137795	MEI1	22
CCDC48	32.82602	5.375134	0.16	ILMN_7566	CCDC48	3
HTR3D	35.3793	5.795397	0.16	ILMN_16646	HTR3D	3
PLCL2	697.226	114.2627	0.16	ILMN_22323	PLCL2	3
ABLIM3	774.2371	127.13	0.16	ILMN_12630	ABLIM3	5
SYNPO2	29.32509	4.81656	0.16	ILMN_23491	SYNPO2	
MRC1L1	11.64339	1.912848	0.16	ILMN_16071	MRC1L1	10
RUNDC3A	108.9772	17.90352	0.16	ILMN_9016	RUNDC3A	17
SEMG1	294.4612	48.62065	0.17	ILMN_171883	SEMG1	20
KIN	855.566	141.4957	0.17	ILMN_17930	KIN	10
ABCG4	1772.003	293.383	0.17	ILMN_10212	ABCG4	11
OR7D2	136.2368	22.63815	0.17	ILMN_29138	OR7D2	19
MS4A14	13.46525	2.244956	0.17	ILMN_182050	MS4A14	11
APBB2	1102.673	184.0344	0.17	ILMN_3902	APBB2	4
DNER	2683.408	449.7177	0.17	ILMN_19114	DNER	2
LOC440558	33.23244	5.577596	0.17	ILMN_20315	LOC440558	10
ATP6V1G2	581.7925	97.83656	0.17	ILMN_18630	ATP6V1G2	6
TMPRSS7	107.9787	18.22665	0.17	ILMN_36290	TMPRSS7	
SPACA5B	132.4753	22.42201	0.17	ILMN_178276	SPACA5B	X
PCDHGA11	37.69355	6.390915	0.17	ILMN_23633	PCDHGA11	5
SLC13A4	36.93582	6.266406	0.17	ILMN_17218	SLC13A4	7
FAT4	17.10615	2.907767	0.17	ILMN_29686	FAT4	4
PORCN	654.5854	111.3324	0.17	ILMN_2528	PORCN	X
GDPD4	1.355599	0.230727	0.17	ILMN_25693	GDPD4	11
NAGK	280.2113	47.69869	0.17	ILMN_4544	NAGK	2
CLEC4E	56.3903	9.605275	0.17	ILMN_136933	CLEC4E	12
KPRP	54.16677	9.241308	0.17	ILMN_27262	KPRP	1
FLJ36208	160.0301	27.32321	0.17	ILMN_8894	FLJ36208	16
TXLNB	144.3224	24.64389	0.17	ILMN_7381	TXLNB	6
CAPN3	305.1064	52.34783	0.17	ILMN_8100	CAPN3	15
TMPRSS6	67.79316	11.63292	0.17	ILMN_28194	TMPRSS6	22
SMA4	332.5093	57.16722	0.17	ILMN_25253	SMA4	

DKFZP586I	343.4344	59.09506	0.17	ILMN_16437	DKFZP586I142	7
DEFB123	31.8555	5.481963	0.17	ILMN_12159	DEFB123	20
C8ORFK32	49.81469	8.588411	0.17	ILMN_7805	C8ORFK32	8
GCNT2	226.9785	39.25635	0.17	ILMN_137985	GCNT2	6
IKIP	211.3291	36.59219	0.17	ILMN_10142	IKIP	12
IGFBP5	894.1653	154.8299	0.17	ILMN_168089	IGFBP5	2
MTTP	208.7675	36.37137	0.17	ILMN_14208	MTTP	4
TEX14	1445.259	253.1808	0.18	ILMN_7591	TEX14	17
DISC1	35.07346	6.145823	0.18	ILMN_27562	DISC1	1
LOC348174	47.2612	8.291327	0.18	ILMN_21133	LOC348174	16
TMEFF1	693.139	121.7058	0.18	ILMN_15840	TMEFF1	9
WFDC3	636.2419	111.9682	0.18	ILMN_22333	WFDC3	20
ANTXR2	1088.706	191.6126	0.18	ILMN_22521	ANTXR2	4
SLC25A20	440.4094	77.56445	0.18	ILMN_29792	SLC25A20	3
PLTP	14.22799	2.505962	0.18	ILMN_12725	PLTP	20
EGFLAM	109.3208	19.26145	0.18	ILMN_21200	EGFLAM	5
C6orf107	96.74839	17.05842	0.18	ILMN_25896	C6ORF107	6
OR8B12	51.12444	9.016852	0.18	ILMN_177412	OR8B12	11
C1orf84	252.3088	44.61025	0.18	ILMN_10202	C1ORF84	1
PRSS1	44.93852	7.966386	0.18	ILMN_26924	PRSS1	7
SYT13	3741.117	665.7645	0.18	ILMN_28065	SYT13	11
TXK	205.5359	36.58661	0.18	ILMN_19719	TXK	4
MUC16	237.2259	42.23665	0.18	ILMN_1949	MUC16	19
ZNF804A	156.7915	27.93598	0.18	ILMN_5026	ZNF804A	2
C6orf65	3364.354	599.6211	0.18	ILMN_27593	C6ORF65	6
EPHA10	141.1865	25.22816	0.18	ILMN_28337	EPHA10	1
EID3	12.15032	2.17934	0.18	ILMN_22737	EID3	12
LIX1	100.3379	17.99941	0.18	ILMN_22811	LIX1	5
FAM3C	2586.981	464.6619	0.18	ILMN_180739	FAM3C	7
RAP2B	379.2189	68.17303	0.18	ILMN_178464	RAP2B	3
OTUD7A	147.1561	26.46433	0.18	ILMN_136923	OTUD7A	15
FLJ41603	2624.685	472.5672	0.18	ILMN_139156	FLJ41603	5
STX11	240.4876	43.36845	0.18	ILMN_3779	STX11	6
FMO4	2174.316	392.5671	0.18	ILMN_1432	FMO4	1
PCDHA9	107.1845	19.37838	0.18	ILMN_170102	PCDHA9	5
RPS6KA4	596.2065	107.9717	0.18	ILMN_4830	RPS6KA4	11
KIAA0513	1416.885	257.2366	0.18	ILMN_21473	KIAA0513	16
NTF3	168.5363	30.61821	0.18	ILMN_18878	NTF3	12
SYNPO2L	242.2682	44.0454	0.18	ILMN_12096	SYNPO2L	10
TMOD2	1394.693	253.7612	0.18	ILMN_12247	TMOD2	15
ZFP3	731.3585	133.0876	0.18	ILMN_137321	ZFP3	17
SAA2	170.9245	31.10886	0.18	ILMN_15509	SAA2	11
SALL4	282.5894	51.46652	0.18	ILMN_18424	SALL4	20
FRMD5	165.2121	30.10155	0.18	ILMN_10697	FRMD5	15
DCLK2	124.1635	22.6454	0.18	ILMN_9577	DCLK2	4
LRRIQ1	31.86472	5.817424	0.18	ILMN_22477	LRRIQ1	12
BLOC1S2	194.2726	35.70534	0.18	ILMN_8691	BLOC1S2	10

OR10H5	100.4778	18.47421	0.18	ILMN_178195	OR10H5	19
DTNB	1166.538	215.132	0.18	ILMN_9450	DTNB	2
ZNF81	418.8516	77.30413	0.18	ILMN_23663	ZNF81	X
OR5T3	4.598081	0.849024	0.18	ILMN_7260	OR5T3	11
FLJ36070	1129.619	208.5849	0.18	ILMN_10833	FLJ36070	19
VEPH1	42.92174	7.962053	0.19	ILMN_11342	VEPH1	3
CCDC7	377.772	70.07895	0.19	ILMN_12205	CCDC7	10
IFIT1L	35.11571	6.543021	0.19	ILMN_5646	IFIT1L	10
SDK2	210.8206	39.3062	0.19	ILMN_26428	SDK2	17
HDAC5	363.083	67.69595	0.19	ILMN_5823	HDAC5	17
AAK1	552.354	103.0511	0.19	ILMN_11466	AAK1	2
KRT6A	2469.599	461.0444	0.19	ILMN_20473	KRT6A	12
AQP8	80.8798	15.11799	0.19	ILMN_11785	AQP8	16
PATE	96.0298	17.97807	0.19	ILMN_24957	PATE	11
FLJ11783	17.9152	3.354134	0.19	ILMN_28082	FLJ11783	11
MFSD11	77.42935	14.50965	0.19	ILMN_7695	MFSD11	17
CAPZA3	70.98451	13.30926	0.19	ILMN_17356	CAPZA3	12
LEMD1	950.9002	178.5877	0.19	ILMN_28526	LEMD1	1
MSTN	371.9628	69.91028	0.19	ILMN_1997	MSTN	2
HLX	70.01087	13.17099	0.19	ILMN_25109	HLX	1
TPM2	5949.18	1119.59	0.19	ILMN_20117	TPM2	9
ZNF829	59.94413	11.31578	0.19	ILMN_1779	ZNF829	19
C2orf52	527.7634	99.67001	0.19	ILMN_170800	C2ORF52	2
GZMK	101.0516	19.12429	0.19	ILMN_10868	GZMK	5
C2orf13	304.4223	57.70199	0.19	ILMN_22786	C2ORF13	2
HSPA1L	880.6465	167.3834	0.19	ILMN_22496	HSPA1L	6
ZNF619	380.5551	72.33643	0.19	ILMN_6902	ZNF619	3
HLA-DRB3	1646.48	313.3264	0.19	ILMN_2942	HLA-DRB3	6
G6PC2	24.3875	4.650632	0.19	ILMN_1509	G6PC2	2
GPR98	203.2735	38.8158	0.19	ILMN_15520	GPR98	5
CNGA3	4.121869	0.787547	0.19	ILMN_13724	CNGA3	2
NAPSA	180.1321	34.42292	0.19	ILMN_12115	NAPSA	19
ZNF713	364.2278	69.63504	0.19	ILMN_885	ZNF713	7
PRLHR	31.53698	6.034863	0.19	ILMN_27818	PRLHR	10
C4orf44	91.07963	17.4334	0.19	ILMN_12335	C4ORF44	4
GPR116	80.36484	15.40891	0.19	ILMN_3315	GPR116	6
C1orf94	89.77184	17.25278	0.19	ILMN_9460	C1ORF94	1
OPRM1	29.93738	5.768335	0.19	ILMN_25847	OPRM1	6
CRX	57.16722	11.01877	0.19	ILMN_139173	CRX	19
LOC642980	87.22787	16.82691	0.19	ILMN_31148	LOC642980	X
TNFSF9	2607.064	503.1407	0.19	ILMN_18030	TNFSF9	19
PRICKLE2	27.51196	5.310256	0.19	ILMN_27101	PRICKLE2	3
SLC5A11	75.98874	14.67222	0.19	ILMN_4902	SLC5A11	16
FAM73A	47.94945	9.259144	0.19	ILMN_4385	FAM73A	1
GNB1L	529.5709	102.3642	0.19	ILMN_21429	GNB1L	22
PACRG	65.47948	12.65706	0.19	ILMN_11772	PACRG	6
SPRR3	166.5304	32.25483	0.19	ILMN_10478	SPRR3	1

C20orf117	794.9259	154.018	0.19	ILMN_3738	C20ORF117	20
HLA-DMB	2078.81	403.0024	0.19	ILMN_2252	HLA-DMB	6
PHYHIP	162.947	31.60404	0.19	ILMN_18437	PHYHIP	8
JPH2	59.75098	11.60936	0.19	ILMN_15587	JPH2	20
LHFP	13.27288	2.581734	0.19	ILMN_8393	LHFP	13
C14orf149	1217.727	237.2259	0.19	ILMN_27351	C14ORF149	14
ATP6V0D2	42.7228	8.32581	0.19	ILMN_19631	ATP6V0D2	8
LOC402057	76.5085	14.97173	0.2	ILMN_181541	LOC402057	22
COL15A1	450.9997	88.28977	0.2	ILMN_15732	COL15A1	9
COL5A3	119.5309	23.4395	0.2	ILMN_16905	COL5A3	19
NDP	353.1895	69.27597	0.2	ILMN_22946	NDP	X
DAAM2	71.3262	13.99331	0.2	ILMN_3540	DAAM2	6
ZCCHC16	105.785	20.76261	0.2	ILMN_17099	ZCCHC16	X
CCL23	305.6114	60.07691	0.2	ILMN_11921	CCL23	17
UBE2B	783.8293	154.7262	0.2	ILMN_19013	UBE2B	5
PCDHGB2	408.7187	80.95267	0.2	ILMN_3682	PCDHGB2	5
GNAI1	1365.842	270.6487	0.2	ILMN_20694	GNAI1	7
RIC8A	986.8546	195.6542	0.2	ILMN_27128	RIC8A	11
MGC29506	169.2806	33.58558	0.2	ILMN_8636	MGC29506	5
PFDN4	2922.801	579.9053	0.2	ILMN_23043	PFDN4	20
OR6A2	35.44197	7.046324	0.2	ILMN_18668	OR6A2	11
SRGN	34.42292	6.845617	0.2	ILMN_2142	SRGN	10
SLFN11	6.812069	1.355599	0.2	ILMN_2517	SLFN11	17
LOC653499	20.70677	4.13673	0.2	ILMN_183137	LOC653499	19
CDC42EP3	5440.463	1089.33	0.2	ILMN_1066	CDC42EP3	2
CTSO	5862.015	1177.177	0.2	ILMN_22132	CTSO	4
ERVWE1	255.7376	51.39768	0.2	ILMN_7784	ERVWE1	7
ZNF765	2231.247	448.4612	0.2	ILMN_178304	ZNF765	19
LOC402573	19.66819	3.955523	0.2	ILMN_9570	LOC402573	7
LOC340156	351.3502	70.87098	0.2	ILMN_3034	LOC340156	6
PTPRT	16.79122	3.397295	0.2	ILMN_29097	PTPRT	20
LOC389607	437.7307	88.60604	0.2	ILMN_17675	LOC389607	8
AGAP2	16.84836	3.413842	0.2	ILMN_11212	AGAP2	12
NR1H4	69.37086	14.10859	0.2	ILMN_25067	NR1H4	12
LYPD5	231.5189	47.10016	0.2	ILMN_12681	LYPD5	19
ITGB8	1300.648	264.6266	0.2	ILMN_3761	ITGB8	7
LOC123688	52.35318	10.65279	0.2	ILMN_20427	LOC123688	15
ZNF423	734.6415	149.7116	0.2	ILMN_164805	ZNF423	16
OR2M5	172.9537	35.2558	0.2	ILMN_23603	OR2M5	1
OR51Q1	92.98584	18.97615	0.2	ILMN_22703	OR51Q1	11
RG9MTD2	458.5005	93.70969	0.2	ILMN_25906	RG9MTD2	4
EMR2	1064.431	217.6266	0.2	ILMN_12015	EMR2	19
APBA2	16.97815	3.47586	0.2	ILMN_4001	APBA2	15
ZBTB40	746.5787	152.9223	0.2	ILMN_6671	ZBTB40	1
CA14	200.7717	41.14272	0.2	ILMN_2282	CA14	1
CHGA	38.00504	7.803576	0.21	ILMN_23390	CHGA	14
KIAA1618	1470.894	302.3521	0.21	ILMN_176692	KIAA1618	17

MDH1B	3821.484	786.0806	0.21	ILMN_13126	MDH1B	2
CNTLN	239.2042	49.40854	0.21	ILMN_25565	CNTLN	9
CKMT1A	328.5542	68.06149	0.21	ILMN_6052	CKMT1A	15
C11orf76	180.3786	37.40866	0.21	ILMN_14041	C11ORF76	11
DUX3	347.6077	72.20004	0.21	ILMN_4458	DUX3	
MPP5	429.4317	89.30682	0.21	ILMN_21733	MPP5	14
OR4D6	44.14692	9.196841	0.21	ILMN_26195	OR4D6	11
FAM69A	184.9458	38.57367	0.21	ILMN_28358	FAM69A	1
IL1RL1	37.11057	7.741325	0.21	ILMN_30234	IL1RL1	2
ITLN2	106.5455	22.22688	0.21	ILMN_828	ITLN2	1
CUL3	560.5604	116.968	0.21	ILMN_7911	CUL3	2
TRDMT1	1342.639	280.1951	0.21	ILMN_13717	TRDMT1	10
ASB15	7.05024	1.472572	0.21	ILMN_8926	ASB15	7
AP1GBP1	891.7519	186.2828	0.21	ILMN_181091	AP1GBP1	17
CLEC4G	101.8483	21.28022	0.21	ILMN_1558	CLEC4G	19
ADIPOQ	23.78462	4.97115	0.21	ILMN_5561	ADIPOQ	3
RNF141	300.8313	62.94351	0.21	ILMN_25007	RNF141	11
ZNF192	1389.516	291.0265	0.21	ILMN_172908	ZNF192	6
HECTD2	457.676	95.91261	0.21	ILMN_459	HECTD2	10
RTN1	209.0615	43.9242	0.21	ILMN_3435	RTN1	14
SEMA4A	261.3982	54.95597	0.21	ILMN_22211	SEMA4A	1
THSD4	726.0206	152.8307	0.21	ILMN_25598	THSD4	15
MUSTN1	330.7903	69.67582	0.21	ILMN_7570	MUSTN1	3
ZNF365	984.3831	207.9411	0.21	ILMN_12691	ZNF365	10
HLA-DOB	2034.789	430.1178	0.21	ILMN_7656	HLA-DOB	6
MXRA7	1548.878	327.6177	0.21	ILMN_8451	MXRA7	17
EEF1E1	855.7182	181.0115	0.21	ILMN_176018	EEF1E1	6
ZNF70	147.8457	31.28133	0.21	ILMN_23213	ZNF70	22
SLC4A3	134.3745	28.46289	0.21	ILMN_6553	SLC4A3	2
CLEC10A	19.95907	4.235034	0.21	ILMN_9747	CLEC10A	17
WDR27	627.0206	133.1366	0.21	ILMN_19074	WDR27	6
LRRC33	58.7702	12.54521	0.21	ILMN_19500	LRRC33	3
ACRV1	133.7318	28.55291	0.21	ILMN_439	ACRV1	11
REEP2	270.555	57.92453	0.21	ILMN_17526	REEP2	5
ZKSCAN5	890.7849	190.8206	0.21	ILMN_27210	ZKSCAN5	7
OR10H2	41.4149	8.871885	0.21	ILMN_19047	OR10H2	19
STAT4	3181.887	681.6308	0.21	ILMN_8937	STAT4	2
CPT1B	148.5851	31.85704	0.21	ILMN_13033	CPT1B	
OR2T8	10.85145	2.328022	0.21	ILMN_10332	OR2T8	1
PLAU	3590.225	771.1321	0.21	ILMN_24167	PLAU	10
HSN2	1472.791	316.4084	0.21	ILMN_4924	HSN2	12
ZDHHC4	7.20241	1.548593	0.22	ILMN_1443	ZDHHC4	7
HTR3A	32.71486	7.067439	0.22	ILMN_23767	HTR3A	11
CALB2	2610.215	564.1585	0.22	ILMN_11415	CALB2	16
CCL3	20.60628	4.454668	0.22	ILMN_1999	CCL3	17
ZNFX1	258.8218	56.02287	0.22	ILMN_1519	ZNFX1	20
RNF175	5.63329	1.222497	0.22	ILMN_29145	RNF175	4

GRID2	117.0155	25.40187	0.22	ILMN_8975	GRID2	4
LOC493869	4837.194	1050.103	0.22	ILMN_7364	LOC493869	5
FRMD6	114.5067	24.87511	0.22	ILMN_25287	FRMD6	14
PCDHAC1	105.9045	23.00924	0.22	ILMN_30169	PCDHAC1	5
ASCL1	24.56224	5.344972	0.22	ILMN_23892	ASCL1	12
TSPYL4	711.1844	155.365	0.22	ILMN_7730	TSPYL4	6
TSPAN5	1154.766	252.3529	0.22	ILMN_8032	TSPAN5	4
SCEL	108.1979	23.68595	0.22	ILMN_15617	SCEL	13
CD1A	484.0705	106.0528	0.22	ILMN_21799	CD1A	1
PCAF	314.1534	68.84483	0.22	ILMN_22596	PCAF	3
ZNF433	153.9524	33.76624	0.22	ILMN_25465	ZNF433	19
TCP10	25.31574	5.565063	0.22	ILMN_2101	TCP10	6
SUSD4	65.59943	14.44167	0.22	ILMN_30253	SUSD4	
CD22	93.84391	20.6662	0.22	ILMN_11761	CD22	19
C18orf34	23.60715	5.217855	0.22	ILMN_26292	C18ORF34	18
PEG10	132.8531	29.36771	0.22	ILMN_165569	PEG10	7
EML1	391.9141	86.78704	0.22	ILMN_8790	EML1	14
ARPP-21	8.506271	1.884509	0.22	ILMN_172653	ARPP-21	3
LCE5A	119.8453	26.55387	0.22	ILMN_7604	LCE5A	1
CALCB	390.3961	86.72695	0.22	ILMN_12713	CALCB	11
HAVCR2	330.3277	73.3958	0.22	ILMN_30017	HAVCR2	5
TNFSF13B	17.0773	3.796769	0.22	ILMN_171368	TNFSF13B	13
SLC30A8	8.871885	1.977045	0.22	ILMN_10814	SLC30A8	8
LRRC23	1009.222	225.3949	0.22	ILMN_22825	LRRC23	12
PPP1R12B	626.3693	140.1114	0.22	ILMN_10582	PPP1R12B	1
CRYBG3	1430.201	320.4149	0.22	ILMN_43895	CRYBG3	3
CYP3A4	44.91535	10.06472	0.22	ILMN_17723	CYP3A4	7
ZNF782	345.244	77.37104	0.22	ILMN_28817	ZNF782	9
NLRP11	515.5491	115.549	0.22	ILMN_10643	NLRP11	19
PPP1R3F	107.363	24.12318	0.22	ILMN_9487	PPP1R3F	X
OR2A14	30.07979	6.763254	0.22	ILMN_22138	OR2A14	7
TAF7L	318.2731	71.57573	0.22	ILMN_13817	TAF7L	X
SULT6B1	11.72214	2.639924	0.23	ILMN_13338	SULT6B1	2
MEF2A	391.8051	88.57584	0.23	ILMN_17271	MEF2A	15
ANKS1B	58.68845	13.26999	0.23	ILMN_28640	ANKS1B	12
ADAMTSL5	222.5853	50.47192	0.23	ILMN_29163	ADAMTSL5	19
CCL16	40.78976	9.251883	0.23	ILMN_8153	CCL16	17
ZNF79	1074.123	243.715	0.23	ILMN_23438	ZNF79	9
FAM154B	674.1046	153.1509	0.23	ILMN_4387	FAM154B	15
DAPK1	79.66809	18.1035	0.23	ILMN_138506	DAPK1	9
ZNF585B	45.60589	10.38026	0.23	ILMN_180453	ZNF585B	19
ZNF22	5551.534	1265.773	0.23	ILMN_165495	ZNF22	10
P2RX6P	11.47521	2.616963	0.23	ILMN_28245	P2RX6P	22
PAX4	4.611505	1.051902	0.23	ILMN_2610	PAX4	7
PRKAA2	816.4131	186.2276	0.23	ILMN_13994	PRKAA2	1
CACNB1	92.88236	21.19447	0.23	ILMN_7465	CACNB1	17
KBTBD3	2288.102	522.2272	0.23	ILMN_24909	KBTBD3	11

MID2	117.5848	26.84571	0.23	ILMN_29777	MID2	X
GYG2	240.8815	55.08442	0.23	ILMN_24869	GYG2	X
RABL5	127.3391	29.17455	0.23	ILMN_24249	RABL5	7
ZCRB1	412.3974	94.51746	0.23	ILMN_9760	ZCRB1	12
ESPNL	1139.483	261.9091	0.23	ILMN_15609	ESPNL	2
PARVG	45.57231	10.48558	0.23	ILMN_21849	PARVG	22
ZNF704	128.6646	29.61091	0.23	ILMN_26345	ZNF704	8
CACNG6	46.46473	10.69588	0.23	ILMN_17399	CACNG6	19
CD59	545.6092	125.6868	0.23	ILMN_1905	CD59	11
MPP1	1573.512	362.797	0.23	ILMN_19796	MPP1	X
CAMK2D	965.8749	222.8436	0.23	ILMN_9877	CAMK2D	4
C10orf88	710.2578	163.9271	0.23	ILMN_20789	C10ORF88	
DUB3	861.7542	199.0714	0.23	ILMN_177752	DUB3	8
SSX4B	108.3134	25.03317	0.23	ILMN_9301	SSX4B	X
NOX5	142.3278	32.90244	0.23	ILMN_13613	NOX5	15
EMP3	4611.165	1068.548	0.23	ILMN_7403	EMP3	19
FLJ16165	58.35132	13.5352	0.23	ILMN_3500	FLJ16165	19
MSMB	16.7211	3.881361	0.23	ILMN_19771	MSMB	10
CES7	54.82631	12.74938	0.23	ILMN_19473	CES7	16
DAO	220.901	51.44716	0.23	ILMN_18156	DAO	12
SSFA2	3074.981	716.1557	0.23	ILMN_4525	SSFA2	2
GALNT14	351.0624	81.79356	0.23	ILMN_29510	GALNT14	2
ZSCAN12	573.3263	133.5848	0.23	ILMN_2619	ZSCAN12	6
ACMSD	72.27975	16.85974	0.23	ILMN_21475	ACMSD	2
ITCH	6853.601	1599.189	0.23	ILMN_17278	ITCH	20
GABBR1	522.7708	122.0083	0.23	ILMN_27683	GABBR1	6
GPLD1	23.53004	5.495847	0.23	ILMN_20927	GPLD1	6
TLR5	2190.503	511.6596	0.23	ILMN_18399	TLR5	1
SLFNL1	15.13085	3.535818	0.23	ILMN_12690	SLFNL1	1
F9	91.82812	21.4659	0.23	ILMN_7077	F9	X
GJC2	141.7814	33.17693	0.23	ILMN_6786	GJC2	1
SCG3	166.7852	39.06705	0.23	ILMN_174345	SCG3	15
CARD8	266.5088	62.44448	0.23	ILMN_13144	CARD8	19
AQP9	16.06721	3.766066	0.23	ILMN_15164	AQP9	15
PI4K2A	1615.95	379.5546	0.23	ILMN_27571	PI4K2A	10
CPO	28.89529	6.790789	0.24	ILMN_12653	CPO	2
DPY19L3	89.19283	20.99853	0.24	ILMN_17111	DPY19L3	19
SOX18	419.3207	98.9866	0.24	ILMN_7425	SOX18	20
TAS2R39	11.58679	2.735324	0.24	ILMN_24652	TAS2R39	7
ZNF484	499.8023	118.3079	0.24	ILMN_2513	ZNF484	9
EBF1	62.91298	14.8975	0.24	ILMN_27934	EBF1	5
SNORD116	16.80508	3.983702	0.24	ILMN_174498	SNORD116-2	15
IL19	34.24549	8.13553	0.24	ILMN_1521	IL19	1
CCDC149	318.8372	75.96925	0.24	ILMN_24542	CCDC149	4
C15orf26	378.5381	90.37892	0.24	ILMN_8667	C15ORF26	15
ELSPBP1	204.4461	48.85691	0.24	ILMN_137880	ELSPBP1	
OR8K3	72.30578	17.28429	0.24	ILMN_6933	OR8K3	11

FAM21A	312.427	74.79826	0.24	ILMN_169691	FAM21A	10
PSORS1C2	278.1833	66.70839	0.24	ILMN_11396	PSORS1C2	6
C9orf21	426.1951	102.4265	0.24	ILMN_16537	C9ORF21	9
AKAP6	143.4823	34.61374	0.24	ILMN_26966	AKAP6	14
TBC1D20	466.5332	112.5601	0.24	ILMN_19840	TBC1D20	20
CCL2	130.041	31.44709	0.24	ILMN_25185	CCL2	17
N4BP3	227.2035	54.9785	0.24	ILMN_16173	N4BP3	5
CLDN17	26.49364	6.412374	0.24	ILMN_19889	CLDN17	21
C3orf32	47.49962	11.54146	0.24	ILMN_10644	C3ORF32	3
GPR52	60.67927	14.77348	0.24	ILMN_176264	GPR52	1
CSTF1	70.89153	17.30083	0.24	ILMN_28406	CSTF1	20
FANK1	254.7421	62.19266	0.24	ILMN_18336	FANK1	10
GRK4	67.40414	16.47353	0.24	ILMN_9882	GRK4	4
AFF2	50.36034	12.32048	0.24	ILMN_137007	AFF2	X
OR2A7	402.668	98.59755	0.24	ILMN_5880	OR2A7	7
OPTN	11097.1	2723.044	0.25	ILMN_23644	OPTN	10
C2orf56	1074.458	263.7606	0.25	ILMN_15591	C2ORF56	2
PSORS1C1	2178.187	535.3464	0.25	ILMN_11981	PSORS1C1	6
C3orf63	51.09453	12.5667	0.25	ILMN_25103	C3ORF63	3
LRRC48	265.6027	65.37109	0.25	ILMN_8796	LRRC48	17
KIRREL2	104.1619	25.65035	0.25	ILMN_10465	KIRREL2	19
PTP4A1	833.4359	205.2458	0.25	ILMN_165831	PTP4A1	6
FBXL13	291.1956	71.78413	0.25	ILMN_2176	FBXL13	7
MEPE	4.67918	1.155249	0.25	ILMN_19864	MEPE	4
FLJ21687	37.40866	9.23735	0.25	ILMN_26889	FLJ21687	X
SLITRK6	96.07603	23.75726	0.25	ILMN_12042	SLITRK6	13
CXCR5	28.51519	7.066062	0.25	ILMN_19896	CXCR5	11
IFT74	1381.117	342.5928	0.25	ILMN_20468	IFT74	9
AGTR1	50.87064	12.62025	0.25	ILMN_17269	AGTR1	3
CSTA	58.99857	14.66026	0.25	ILMN_10501	CSTA	3
C15orf33	102.787	25.5458	0.25	ILMN_5719	C15ORF33	15
ARHGAP22	179.5852	44.64705	0.25	ILMN_15801	ARHGAP22	10
SLC41A2	3719.432	925.1235	0.25	ILMN_14620	SLC41A2	12
LOC340529	57.59	14.32848	0.25	ILMN_809	LOC340529	X
PPP1R1C	32.65539	8.125217	0.25	ILMN_36531	PPP1R1C	2
GSR	1188.266	295.7378	0.25	ILMN_14467	GSR	8
C7orf52	910.8334	227.0467	0.25	ILMN_183724	C7ORF52	7
LMO3	17.56203	4.383659	0.25	ILMN_28403	LMO3	12
KLK13	2084.426	520.5025	0.25	ILMN_14934	KLK13	19
CD160	112.3769	28.06804	0.25	ILMN_15995	CD160	1
LOC552891	7.026533	1.75545	0.25	ILMN_29106	LOC552891	9
ZNF609	1244.367	311.2444	0.25	ILMN_28956	ZNF609	15
KIF5A	1111.978	278.1833	0.25	ILMN_8502	KIF5A	12
BSN	1991.871	499.1614	0.25	ILMN_22754	BSN	3
PDE4DIP	710.4961	178.2952	0.25	ILMN_14160	PDE4DIP	1
AGPAT4	5190.452	1304.14	0.25	ILMN_14756	AGPAT4	6
GPBAR1	119.8814	30.1389	0.25	ILMN_7981	GPBAR1	2

PANX2	2634.898	662.9407	0.25	ILMN_4796	PANX2	22
WTIP	108.161	27.23721	0.25	ILMN_171645	WTIP	19
EGR4	13.40201	3.377379	0.25	ILMN_25450	EGR4	2
PLEKHG1	825.2372	208.1315	0.25	ILMN_176922	PLEKHG1	6
PRO0628	100.9853	25.47354	0.25	ILMN_9963	PRO0628	20
NR4A1	127.481	32.16511	0.25	ILMN_26702	NR4A1	12
B4GALNT1	327.8291	82.72047	0.25	ILMN_137775	B4GALNT1	12
PRSS35	4.133403	1.043104	0.25	ILMN_18255	PRSS35	6
ADCYAP1R	3.912244	0.988254	0.25	ILMN_672	ADCYAP1R1	7
OR1D4	37.21667	9.404236	0.25	ILMN_1050	OR1D4	
NTAN1	1846.311	466.8104	0.25	ILMN_28116	NTAN1	16
MR1	1452.321	367.814	0.25	ILMN_10108	MR1	1
RFESD	658.3137	166.864	0.25	ILMN_174243	RFESD	5
SPATA6	1157.296	293.4945	0.25	ILMN_20491	SPATA6	1
PHLDA2	352.2497	89.36786	0.25	ILMN_11855	PHLDA2	11
DUSP22	1614.849	410.5874	0.25	ILMN_15436	DUSP22	6
ANKRD44	185.7127	47.22472	0.25	ILMN_17623	ANKRD44	2
SMU1	213.7144	54.36888	0.25	ILMN_5002	SMU1	9
KRT80	1479.053	376.4954	0.25	ILMN_3629	KRT80	12
BICD1	315.2115	80.31453	0.25	ILMN_13964	BICD1	12
FAM174A	196.5323	50.13405	0.26	ILMN_1952	FAM174A	5
SERPINC1	202.6321	51.75877	0.26	ILMN_18442	SERPINC1	1
CMYA3	2.38494	0.610318	0.26	ILMN_162230	CMYA3	
SYN1	181.467	46.44414	0.26	ILMN_22497	SYN1	X
TUBB4	1403.914	359.6614	0.26	ILMN_23388	TUBB4	19
SLC9A7	182.7655	46.93176	0.26	ILMN_16442	SLC9A7	X
ERCC6	216.8951	55.73832	0.26	ILMN_20934	ERCC6	10
KRTAP3-1	206.9987	53.20362	0.26	ILMN_23562	KRTAP3-1	
AKR1B10	627.7211	161.4375	0.26	ILMN_6435	AKR1B10	7
CLDN19	65.0827	16.7489	0.26	ILMN_6731	CLDN19	1
HTR2A	16.9868	4.379795	0.26	ILMN_18234	HTR2A	13
LRRN2	659.3841	170.1909	0.26	ILMN_21516	LRRN2	1
FAM129A	218.3172	56.3719	0.26	ILMN_6530	FAM129A	1
PIR	634.2599	163.9781	0.26	ILMN_13999	PIR	X
TUBGCP3	1120.15	289.5985	0.26	ILMN_17210	TUBGCP3	13
FLJ45964	76.96004	19.89758	0.26	ILMN_3733	FLJ45964	2
VAPA	1084.176	280.725	0.26	ILMN_22710	VAPA	18
C1orf21	1297.205	336.1038	0.26	ILMN_26434	C1ORF21	1
LOC143188	70.88796	18.37093	0.26	ILMN_22064	LOC143188	10
CLCA4	8.445633	2.194938	0.26	ILMN_17695	CLCA4	1
SGEF	378.0001	98.24422	0.26	ILMN_2696	SGEF	3
FLJ21986	2061.869	535.9639	0.26	ILMN_22149	FLJ21986	7
RAC2	763.0359	198.6153	0.26	ILMN_26813	RAC2	22
THBS1	1726.148	449.3835	0.26	ILMN_4882	THBS1	15
ACTN2	251.9322	65.65027	0.26	ILMN_798	ACTN2	1
PRKAR2B	334.7214	87.37419	0.26	ILMN_6329	PRKAR2B	7
ST3GAL3	477.4888	124.696	0.26	ILMN_13266	ST3GAL3	1

CYP39A1	48.17558	12.60345	0.26	ILMN_164980	CYP39A1	6
CDKN1A	1539.232	402.7405	0.26	ILMN_5895	CDKN1A	6
CDH4	25.85858	6.767826	0.26	ILMN_19514	CDH4	20
PSD	157.1401	41.18595	0.26	ILMN_22468	PSD	10
SLC5A3	694.3295	182.0687	0.26	ILMN_11699	SLC5A3	21
THEM4	1711.969	449.003	0.26	ILMN_28714	THEM4	1
C6orf204	547.6588	143.9238	0.26	ILMN_180771	C6ORF204	6
ZMYM6	2092.315	550.9272	0.26	ILMN_1275	ZMYM6	1
ZCWPW2	227.3574	59.87467	0.26	ILMN_177758	ZCWPW2	3
KRTAP22-1	4.568677	1.205213	0.26	ILMN_24494	KRTAP22-1	21
KIF3A	319.2524	84.29441	0.26	ILMN_138303	KIF3A	5
YIPF1	588.7916	155.7346	0.26	ILMN_19321	YIPF1	1
ANKRD29	2162.916	572.6404	0.26	ILMN_177949	ANKRD29	18
HOXD13	308.3037	81.67829	0.26	ILMN_27639	HOXD13	2
GABRD	664.6799	176.1141	0.26	ILMN_22124	GABRD	1
TRIM62	53.91426	14.29284	0.27	ILMN_4460	TRIM62	1
CADM4	1504.37	398.8583	0.27	ILMN_19958	CADM4	19
PROL1	41.59319	11.02959	0.27	ILMN_12501	PROL1	4
MAGEC2	497.6483	132.2844	0.27	ILMN_14671	MAGEC2	X
OR52W1	59.16819	15.73225	0.27	ILMN_18614	OR52W1	11
OXTR	398.0041	105.946	0.27	ILMN_7313	OXTR	3
GLUL	164.8655	43.91639	0.27	ILMN_25881	GLUL	1
HLA-DOA	306.8107	81.74017	0.27	ILMN_27857	HLA-DOA	6
HS3ST4	3.425587	0.913405	0.27	ILMN_21304	HS3ST4	16
RNF32	94.75815	25.27429	0.27	ILMN_17994	RNF32	7
KCTD7	92.55332	24.70487	0.27	ILMN_2045	KCTD7	7
HNRNPA3	1002.566	267.7828	0.27	ILMN_5256	HNRNPA3	2
LMO7	1484.88	396.613	0.27	ILMN_16493	LMO7	13
PAQR6	426.583	114.1583	0.27	ILMN_18415	PAQR6	1
LOC390110	51.95071	13.91135	0.27	ILMN_2539	LOC390110	11
TIRAP	59.70665	15.99159	0.27	ILMN_138111	TIRAP	11
LOC441268	1305.173	349.8321	0.27	ILMN_18016	LOC441268	7
KRT81	303.7827	81.507	0.27	ILMN_635	KRT81	12
C9orf25	172.062	46.20458	0.27	ILMN_22325	C9ORF25	9
SOX21	77.12568	20.72784	0.27	ILMN_11392	SOX21	13
CCL1	6.613192	1.780367	0.27	ILMN_8339	CCL1	17
MTHFSD	1622.409	436.891	0.27	ILMN_23689	MTHFSD	16
PKLR	30.1793	8.127477	0.27	ILMN_137806	PKLR	1
ABHD13	1208.537	325.5207	0.27	ILMN_182273	ABHD13	13
SMAP1	362.6481	97.71638	0.27	ILMN_1404	SMAP1	6
OPN1SW	21.53262	5.809435	0.27	ILMN_7107	OPN1SW	7
KIAA0514	905.646	244.5865	0.27	ILMN_4697	KIAA0514	10
LELP1	13.91135	3.758005	0.27	ILMN_14694	LELP1	1
TAS2R4	11.76053	3.178663	0.27	ILMN_14113	TAS2R4	7
CAPG	307.6214	83.24951	0.27	ILMN_2712	CAPG	2
RNF10	993.7407	270.0099	0.27	ILMN_14490	RNF10	12
TAF1B	479.6312	130.432	0.27	ILMN_13234	TAF1B	2

NHSL2	50.47466	13.73982	0.27	ILMN_24309	NHSL2	X
FOXP4	1201.045	327.3282	0.27	ILMN_176687	FOXP4	6
AMHR2	397.7557	108.4258	0.27	ILMN_30245	AMHR2	12
PARP8	1774.143	483.9186	0.27	ILMN_26673	PARP8	5
COL9A3	158.8409	43.34837	0.27	ILMN_29219	COL9A3	20
CTAGE5	557.978	152.3821	0.27	ILMN_28819	CTAGE5	14
TPH1	49.93894	13.65151	0.27	ILMN_5420	TPH1	11
C8ORFK36	35.02393	9.575261	0.27	ILMN_177508	C8ORFK36	8
CD200	67.44413	18.44641	0.27	ILMN_137798	CD200	3
OR10AD1	44.32087	12.12961	0.27	ILMN_18463	OR10AD1	12
STAC2	53.96705	14.78628	0.27	ILMN_28417	STAC2	17
SLMO1	204.5648	56.04868	0.27	ILMN_13331	SLMO1	18
IAPP	56.68245	15.58519	0.27	ILMN_30144	IAPP	12
ADHFE1	197.7241	54.43134	0.28	ILMN_169488	ADHFE1	
ABCG2	344.8657	94.94296	0.28	ILMN_27505	ABCG2	4
CCDC69	28.63776	7.890753	0.28	ILMN_12667	CCDC69	5
PDK1	247.1809	68.14708	0.28	ILMN_18922	PDK1	2
PROK1	26.32231	7.259387	0.28	ILMN_10836	PROK1	1
SPOPL	2304.106	636.1888	0.28	ILMN_21364	SPOPL	2
RABGGTB	884.5291	244.273	0.28	ILMN_25242	RABGGTB	1
IL1RAP	1155.548	319.1234	0.28	ILMN_21742	IL1RAP	3
RPL37	214.0099	59.16819	0.28	ILMN_18811	RPL37	5
LCE2A	8.370605	2.31447	0.28	ILMN_4011	LCE2A	1
MGC4677	4344.13	1202.507	0.28	ILMN_137135	MGC4677	2
WIPI1	2144.781	594.1481	0.28	ILMN_19646	WIPI1	
NPHS1	540.0337	149.6472	0.28	ILMN_4764	NPHS1	19
IGSF11	32.76133	9.094457	0.28	ILMN_1669	IGSF11	3
MSRB3	1088.17	302.2047	0.28	ILMN_24072	MSRB3	12
MAPKBP1	170.1994	47.27995	0.28	ILMN_14955	MAPKBP1	15
STYK1	1032.524	287.3413	0.28	ILMN_182051	STYK1	12
KRT7	240.7776	67.04951	0.28	ILMN_12486	KRT7	12
KLHL18	116.0935	32.35258	0.28	ILMN_27249	KLHL18	3
C10orf6	10001.2	2790.301	0.28	ILMN_24540	C10ORF6	10
CLUL1	46.32041	12.92342	0.28	ILMN_4054	CLUL1	18
GSTA4	735.0571	205.12	0.28	ILMN_18558	GSTA4	6
DISP1	744.5679	207.8133	0.28	ILMN_17841	DISP1	1
PTPRC	40.09282	11.19373	0.28	ILMN_27168	PTPRC	1
TXNDC4	608.4944	170.0063	0.28	ILMN_176683	TXNDC4	9
ZFYVE1	128.7821	36.00172	0.28	ILMN_6420	ZFYVE1	14
PKD2	560.727	156.87	0.28	ILMN_167901	PKD2	4
NPC1L1	175.5125	49.10764	0.28	ILMN_8618	NPC1L1	7
C3	163.978	45.93238	0.28	ILMN_33011	C3	19
ATF3	719.308	201.658	0.28	ILMN_6468	ATF3	1
ST8SIA5	503.6277	141.2015	0.28	ILMN_2031	ST8SIA5	18
POMT2	1997.389	561.9299	0.28	ILMN_21092	POMT2	14
CAPN13	219.2493	61.80871	0.28	ILMN_25604	CAPN13	2
LYSMD1	269.0374	75.88422	0.28	ILMN_5871	LYSMD1	1

AMN1	3015.465	850.9547	0.28	ILMN_29296	AMN1	12
PRKCQ	1864.635	526.8947	0.28	ILMN_22905	PRKCQ	10
JAM3	124.5882	35.2062	0.28	ILMN_25295	JAM3	11
FUT5	94.321	26.66038	0.28	ILMN_23715	FUT5	19
GIP	10.91591	3.088431	0.28	ILMN_29326	GIP	17
CCDC102B	48.57388	13.7436	0.28	ILMN_27349	CCDC102B	18
DCBLD2	9153.363	2591.482	0.28	ILMN_175741	DCBLD2	3
ADAM21	48.45254	13.72218	0.28	ILMN_29533	ADAM21	14
GRK5	600.3399	170.2515	0.28	ILMN_177586	GRK5	10
FGD1	1343.384	381.0739	0.28	ILMN_20342	FGD1	X
NAGA	2937.721	833.4359	0.28	ILMN_21273	NAGA	22
DUSP6	1148.967	326.2411	0.28	ILMN_5440	DUSP6	12
PRRG4	68.89868	19.5643	0.28	ILMN_26652	PRRG4	11
SFXN3	586.8646	166.7852	0.28	ILMN_3166	SFXN3	10
ZNF665	20.54984	5.842791	0.28	ILMN_22424	ZNF665	19
LRAT	87.11626	24.79227	0.28	ILMN_29431	LRAT	4
C20orf100	19472.22	5545.132	0.28	ILMN_17741	C20ORF100	20
AP1S3	24.13342	6.872706	0.28	ILMN_15204	AP1S3	2
DCDC5	596.5453	170.0542	0.29	ILMN_36614	DCDC5	11
ATG4A	1636.894	468.5518	0.29	ILMN_27268	ATG4A	X
IRF4	45.82649	13.12115	0.29	ILMN_12414	IRF4	6
FBXO42	344.9917	98.80527	0.29	ILMN_17967	FBXO42	1
RARG	11.67248	3.34917	0.29	ILMN_12771	RARG	12
RYR3	27.80376	7.981238	0.29	ILMN_22832	RYR3	15
ZNF611	240.3264	68.99734	0.29	ILMN_4272	ZNF611	19
ZNF641	651.6046	187.4239	0.29	ILMN_7317	ZNF641	12
RPL13L	199.1335	57.30141	0.29	ILMN_14409	RPL13L	12
ARL4C	142.458	41.04934	0.29	ILMN_15416	ARL4C	2
SLC22A17	309.1031	89.07014	0.29	ILMN_10076	SLC22A17	14
HIST1H3C	69.3461	20.00874	0.29	ILMN_23964	HIST1H3C	6
PFKFB1	49.42797	14.27834	0.29	ILMN_23518	PFKFB1	X
CLLU1OS	39.86795	11.5239	0.29	ILMN_29565	CLLU1OS	12
GSTTP2	2.391181	0.692464	0.29	ILMN_179489	GSTTP2	22
IKBKAP	818.6238	237.1394	0.29	ILMN_30229	IKBKAP	9
SH2B3	849.498	246.1126	0.29	ILMN_5130	SH2B3	12
NLRP7	23.7905	6.897019	0.29	ILMN_2155	NLRP7	19
ABL2	654.5251	189.9222	0.29	ILMN_8230	ABL2	1
DCLRE1A	19.07454	5.540722	0.29	ILMN_1346	DCLRE1A	10
LOXL1	318.1438	92.43183	0.29	ILMN_7655	LOXL1	15
NSD1	443.8468	128.9871	0.29	ILMN_2264	NSD1	5
TEAD1	104.8104	30.46522	0.29	ILMN_33862	TEAD1	11
DEFB119	37.65492	10.94755	0.29	ILMN_1862	DEFB119	20
OR2T6	1.57249	0.457231	0.29	ILMN_26902	OR2T6	1
SCN1B	61.04524	17.76177	0.29	ILMN_6044	SCN1B	19
ZNF264	1095.583	319.2524	0.29	ILMN_7722	ZNF264	19
TMEM158	6275.091	1829.406	0.29	ILMN_13668	TMEM158	3
PHF20	277.0581	80.78345	0.29	ILMN_20893	PHF20	20

CCL24	95.41048	27.82706	0.29	ILMN_17285	CCL24	7
NOX1	37.24879	10.86692	0.29	ILMN_2578	NOX1	X
NTF5	144.5644	42.17519	0.29	ILMN_20683	NTF5	19
DUSP5	6252.548	1824.763	0.29	ILMN_12928	DUSP5	10
CLCF1	468.2392	136.6808	0.29	ILMN_10458	CLCF1	11
GNLY	14.45887	4.223272	0.29	ILMN_17459	GNLY	2
CD3E	31.89981	9.321294	0.29	ILMN_24240	CD3E	11
PIK3R3	2.739221	0.800666	0.29	ILMN_21213	PIK3R3	1
KCTD21	1236.47	361.5011	0.29	ILMN_9060	KCTD21	11
MAP3K3	75.6625	22.13955	0.29	ILMN_170574	MAP3K3	17
SCHIP1	3465.578	1015.852	0.29	ILMN_29529	SCHIP1	3
LRRC37A3	131.3729	38.53199	0.29	ILMN_17947	LRRC37A3	17
ICA1L	12.96104	3.804453	0.29	ILMN_20842	ICA1L	2
GALNAC4S-	5684.215	1670.567	0.29	ILMN_8597	GALNAC4S-6ST	10
C8orf31	13.06512	3.84056	0.29	ILMN_15334	C8ORF31	8
CARTPT	146.548	43.08228	0.29	ILMN_13109	CARTPT	5
SEC14L2	290.2023	85.35262	0.29	ILMN_9607	SEC14L2	22
NUDT9P1	238.9074	70.27749	0.29	ILMN_11515	NUDT9P1	10
JAZF1	2623.736	771.8865	0.29	ILMN_17367	JAZF1	7
LANCL3	1559.774	459.1659	0.29	ILMN_27739	LANCL3	X
TBRG1	606.6799	178.6551	0.29	ILMN_4946	TBRG1	11
TRMT5	430.3457	126.7695	0.29	ILMN_162498	TRMT5	14
SLC4A4	102.4265	30.1769	0.29	ILMN_176000	SLC4A4	4
C20orf195	439.9292	129.7327	0.29	ILMN_7222	C20ORF195	20
PLEKHA7	1116.084	329.1515	0.29	ILMN_17656	PLEKHA7	11
ERCC4	713.476	210.8206	0.3	ILMN_17387	ERCC4	16
DKFZp686D	133.3689	39.43869	0.3	ILMN_21631	DKFZP686D09	5
C16orf46	42.83987	12.68355	0.3	ILMN_22996	C16ORF46	16
UBASH3A	24.50075	7.259394	0.3	ILMN_27169	UBASH3A	21
GFPT2	1687.88	500.3781	0.3	ILMN_7613	GFPT2	5
CAPN2	1861.906	552.3679	0.3	ILMN_24443	CAPN2	1
OR10J1	40.17414	11.92188	0.3	ILMN_170664	OR10J1	1
ELAVL4	7.931752	2.353896	0.3	ILMN_17338	ELAVL4	1
SPG20	146.9828	43.62801	0.3	ILMN_4767	SPG20	13
RAB41	33.0879	9.825379	0.3	ILMN_2027	RAB41	X
SLC4A5	106.3628	31.61758	0.3	ILMN_9219	SLC4A5	2
SLC26A9	24.27944	7.23084	0.3	ILMN_3106	SLC26A9	1
CERKL	41.57168	12.39368	0.3	ILMN_8264	CERKL	2
REPS2	268.455	80.04169	0.3	ILMN_175412	REPS2	X
NR3C2	201.5068	60.09818	0.3	ILMN_166092	NR3C2	4
MYCL1	30.23607	9.023103	0.3	ILMN_19031	MYCL1	1
KCNH6	74.22955	22.15295	0.3	ILMN_13980	KCNH6	17
STXBP1	525.4263	156.8429	0.3	ILMN_10266	STXBP1	9
LOC285359	215.3456	64.29987	0.3	ILMN_45268	LOC285359	3
GPR162	1100.299	328.6581	0.3	ILMN_4999	GPR162	12
OGFRL1	1334.636	398.6656	0.3	ILMN_162668	OGFRL1	6
MTF1	613.7891	183.5169	0.3	ILMN_24958	MTF1	1

TUBB3	11166.24	3339.956	0.3	ILMN_16399	TUBB3	16
RBM43	214.6996	64.275	0.3	ILMN_871	RBM43	2
NXPH1	60.28288	18.06916	0.3	ILMN_19430	NXPH1	7
TUBB2A	780.8574	234.1165	0.3	ILMN_8135	TUBB2A	6
RAD9B	399.9979	119.9804	0.3	ILMN_22564	RAD9B	12
DARC	22.60136	6.780001	0.3	ILMN_25660	DARC	1
HAND1	836.369	250.9352	0.3	ILMN_29799	HAND1	5
CR2	365.0457	109.5438	0.3	ILMN_26378	CR2	1
FSTL3	438.2646	131.6209	0.3	ILMN_13221	FSTL3	19
FLJ90650	295.7378	88.83228	0.3	ILMN_5784	FLJ90650	5
CALML6	22.0406	6.620543	0.3	ILMN_29310	CALML6	1
MRAP	24.33958	7.313967	0.3	ILMN_23154	MRAP	21
STRC	239.078	71.9166	0.3	ILMN_180253	STRC	15
CBLN3	1022.2	307.8237	0.3	ILMN_45604	CBLN3	14
OR5P3	6.371327	1.919179	0.3	ILMN_23678	OR5P3	11
CDC42SE1	503.5885	151.6943	0.3	ILMN_2548	CDC42SE1	1
CTTNBP2	35.01751	10.55483	0.3	ILMN_23721	CTTNBP2	7
KIAA2018	119.6223	36.07699	0.3	ILMN_29746	KIAA2018	3
CXorf39	3606.5	1088.232	0.3	ILMN_18853	CXORF39	X
PCGF1	613.2676	185.0735	0.3	ILMN_5720	PCGF1	2
8-Mar	1038.83	313.55	0.3	ILMN_6980	8-Mar	10
PCDHGA9	57.75311	17.43709	0.3	ILMN_22537	PCDHGA9	5
LOC440944	145.8011	44.02599	0.3	ILMN_19496	LOC440944	
GPR139	8.028133	2.424252	0.3	ILMN_6038	GPR139	16
KCNK7	91.61137	27.66899	0.3	ILMN_26394	KCNK7	11
LOC400965	93.03408	28.16004	0.3	ILMN_14891	LOC400965	2
ADRA2A	80.19067	24.29955	0.3	ILMN_23205	ADRA2A	10
TM7SF3	1608.864	487.5307	0.3	ILMN_7797	TM7SF3	12
GATC	1776.265	538.6461	0.3	ILMN_16367	GATC	12
C5	641.4117	194.7353	0.3	ILMN_28154	C5	9
SENP7	473.8407	144.1183	0.3	ILMN_167055	SENP7	3
KIAA1919	289.5115	88.06847	0.3	ILMN_19440	KIAA1919	6
C6orf1	67.80663	20.64808	0.3	ILMN_11972	C6ORF1	6
LOC342897	42.2836	12.93073	0.31	ILMN_1768	LOC342897	19
C18orf45	110.3609	33.75409	0.31	ILMN_7824	C18ORF45	18
CLIC5	100.4366	30.71965	0.31	ILMN_137813	CLIC5	6
HCRTR2	3.045532	0.931696	0.31	ILMN_4206	HCRTR2	6
TTLL7	1042.36	319.1518	0.31	ILMN_8678	TTLL7	1
OR8A1	20.04987	6.151597	0.31	ILMN_4625	OR8A1	11
KCTD1	247.9387	76.1011	0.31	ILMN_19231	KCTD1	18
MLL3	478.9237	147.1	0.31	ILMN_14020	MLL3	7
PECI	1760.141	540.8658	0.31	ILMN_7427	PECI	6
COL13A1	295.1025	90.81323	0.31	ILMN_25843	COL13A1	10
BFSP1	425.6246	131.056	0.31	ILMN_27828	BFSP1	20
C17orf48	2839.66	874.63	0.31	ILMN_6506	C17ORF48	17
UTS2	58.16283	17.92193	0.31	ILMN_28044	UTS2	1
BTN1A1	300.0721	92.62143	0.31	ILMN_8270	BTN1A1	6

ALG13	1052.924	325.3168	0.31	ILMN_3125	ALG13	X
LRRC46	1.199141	0.370854	0.31	ILMN_15794	LRRC46	17
SELT	501.1183	155.1007	0.31	ILMN_174944	SELT	3
ACSBG2	206.9349	64.07219	0.31	ILMN_138348	ACSBG2	19
SOX12	175.7544	54.48348	0.31	ILMN_21880	SOX12	20
FLYWCH1	360.1797	111.6678	0.31	ILMN_21699	FLYWCH1	16
RUNDC3B	2420.512	750.5863	0.31	ILMN_28536	RUNDC3B	7
OR4C12	53.78195	16.69234	0.31	ILMN_175385	OR4C12	11
NTN4	4161.622	1293.079	0.31	ILMN_29679	NTN4	12
KIAA0467	475.7281	147.8457	0.31	ILMN_25115	KIAA0467	1
OFD1	2784.456	865.9567	0.31	ILMN_10088	OFD1	X
OR10K2	87.14108	27.11033	0.31	ILMN_23488	OR10K2	1
ACOT12	10.81385	3.364473	0.31	ILMN_12512	ACOT12	5
FLJ22795	888.7421	276.5967	0.31	ILMN_1721	FLJ22795	15
WWP1	795.7709	247.805	0.31	ILMN_164871	WWP1	8
KLF15	323.1306	100.6635	0.31	ILMN_23686	KLF15	3
APC2	173.4302	54.03734	0.31	ILMN_4619	APC2	19
PLEC1	389.7996	121.59	0.31	ILMN_24283	PLEC1	8
XPA	111.9296	34.95862	0.31	ILMN_28155	XPA	9
CCL3L1	24.64389	7.696952	0.31	ILMN_6158	CCL3L1	17
ZC3H10	1235.922	386.5722	0.31	ILMN_14237	ZC3H10	12
DNAJC21	474.2354	148.3994	0.31	ILMN_15289	DNAJC21	5
PTK2B	228.9246	71.67164	0.31	ILMN_24969	PTK2B	8
TFDP2	11.22394	3.514736	0.31	ILMN_183128	TFDP2	3
PPP4R4	573.3337	179.5468	0.31	ILMN_169533	PPP4R4	14
OR2A25	15.1076	4.732586	0.31	ILMN_10926	OR2A25	7
ABHD8	1836.1	575.282	0.31	ILMN_23791	ABHD8	19
C15orf5	503.7695	157.9116	0.31	ILMN_15432	C15ORF5	
EVI2B	16.18067	5.076514	0.31	ILMN_171578	EVI2B	17
TRPM2	149.5477	46.93122	0.31	ILMN_27177	TRPM2	21
RAB18	1124.243	352.8846	0.31	ILMN_13827	RAB18	10
ARMC7	843.6371	264.8951	0.31	ILMN_25900	ARMC7	17
DDR1	545.02	171.1506	0.31	ILMN_10004	DDR1	6
ZMAT5	535.3602	168.2651	0.31	ILMN_13941	ZMAT5	22
CCDC148	418.5232	131.5441	0.31	ILMN_1366	CCDC148	2
AP3S2	737.7824	231.9394	0.31	ILMN_25905	AP3S2	15
LCT	163.8398	51.51281	0.31	ILMN_165828	LCT	2
M6PRBP1	1980.494	623.3774	0.31	ILMN_10971	M6PRBP1	19
TMEM155	44.66234	14.06102	0.31	ILMN_6710	TMEM155	4
LRRC6	152.2798	47.99477	0.32	ILMN_13455	LRRC6	8
PER1	44.0454	13.8876	0.32	ILMN_16811	PER1	17
ZNF554	1660.342	524.0451	0.32	ILMN_10156	ZNF554	
PPM2C	4071.528	1287.171	0.32	ILMN_163087	PPM2C	8
ETNK2	461.6527	146.1004	0.32	ILMN_4993	ETNK2	1
SH3BP2	417.9958	132.4753	0.32	ILMN_1151	SH3BP2	4
SMG6	67.32726	21.34183	0.32	ILMN_20830	SMG6	17
SUFU	23.94402	7.590467	0.32	ILMN_23297	SUFU	10

BIRC3	936.7646	297.4335	0.32	ILMN_4117	BIRC3	11
NR4A2	1411.47	448.1785	0.32	ILMN_28405	NR4A2	2
FLJ40243	43.8315	13.92629	0.32	ILMN_29980	FLJ40243	5
OR2L8	83.89141	26.67277	0.32	ILMN_12197	OR2L8	1
HADHB	1039.26	330.7903	0.32	ILMN_14786	HADHB	2
PKD1L1	159.7362	50.84435	0.32	ILMN_169848	PKD1L1	7
SLN	82.10794	26.14519	0.32	ILMN_22283	SLN	11
RPAP2	2932.461	934.0353	0.32	ILMN_23904	RPAP2	1
PPP2R5B	2215.977	706.6072	0.32	ILMN_7752	PPP2R5B	11
AFF1	245.1512	78.33949	0.32	ILMN_170504	AFF1	4
MAPK11	230.1455	73.56204	0.32	ILMN_23755	MAPK11	22
FMO3	57.22444	18.29155	0.32	ILMN_18262	FMO3	1
C10orf114	205.4736	65.69332	0.32	ILMN_26554	C10ORF114	10
ENPP1	210.1979	67.22791	0.32	ILMN_12347	ENPP1	6
BACH2	457.6947	146.6591	0.32	ILMN_23977	BACH2	6
KIAA1166	8.306143	2.663823	0.32	ILMN_19438	KIAA1166	X
MBD6	299.198	95.97881	0.32	ILMN_26050	MBD6	12
L3MBTL3	2563.535	822.5226	0.32	ILMN_24970	L3MBTL3	6
QSER1	603.7545	193.797	0.32	ILMN_167598	QSER1	11
KIAA1279	650.9964	209.4503	0.32	ILMN_170659	KIAA1279	10
PTTG2	453.3138	145.8956	0.32	ILMN_25893	PTTG2	4
FILIP1L	117.8601	37.96875	0.32	ILMN_18421	FILIP1L	3
SNX21	509.4135	164.229	0.32	ILMN_9972	SNX21	20
UNC119	249.2472	80.3643	0.32	ILMN_20431	UNC119	17
GDF10	4.087409	1.319259	0.32	ILMN_2613	GDF10	10
DLX4	192.509	62.14059	0.32	ILMN_3647	DLX4	17
CCBP2	416.0488	134.5421	0.32	ILMN_11666	CCBP2	3
FALZ	633.1429	204.7878	0.32	ILMN_27450	FALZ	17
RGL4	45.38566	14.69734	0.32	ILMN_28738	RGL4	22
FRG2B	82.39996	26.68581	0.32	ILMN_39248	FRG2B	10
GDI1	1591.121	515.8011	0.32	ILMN_13492	GDI1	X
KIAA1467	309.8957	100.4778	0.32	ILMN_26339	KIAA1467	12
SRGAP2	304.5083	98.85293	0.32	ILMN_7259	SRGAP2	1
TMOD3	1012.693	328.8122	0.32	ILMN_28026	TMOD3	15
ZNF648	319.7914	103.8716	0.32	ILMN_12997	ZNF648	1
APBB1IP	26.31229	8.548553	0.32	ILMN_19412	APBB1IP	10
OSBPL3	1848.959	600.9543	0.33	ILMN_17380	OSBPL3	7
MICA	347.9045	113.078	0.33	ILMN_10987	MICA	6
IL6R	73.31957	23.83751	0.33	ILMN_22419	IL6R	1
NBPF7	24.70487	8.035199	0.33	ILMN_171393	NBPF7	1
STARD4	309.2964	100.6955	0.33	ILMN_27466	STARD4	5
ZNF366	49.7245	16.20185	0.33	ILMN_9709	ZNF366	5
MIER2	395.1192	128.8406	0.33	ILMN_19437	MIER2	19
RNF214	836.998	272.9389	0.33	ILMN_29644	RNF214	11
ZNF197	999.1938	326.0283	0.33	ILMN_1508	ZNF197	3
PDYN	95.58485	31.19798	0.33	ILMN_10826	PDYN	20
ZNF286A	1865.552	608.9205	0.33	ILMN_16483	ZNF286A	17

OR2A1	175.586	57.35855	0.33	ILMN_166257	OR2A1	7
MGC35440	11.56556	3.780108	0.33	ILMN_3839	MGC35440	19
RASSF5	449.317	146.8817	0.33	ILMN_690	RASSF5	1
FEZ1	52.50446	17.16865	0.33	ILMN_419	FEZ1	11
ADH5	175.1444	57.2794	0.33	ILMN_2956	ADH5	4
TSPAN4	384.4724	125.7861	0.33	ILMN_9326	TSPAN4	11
RFXAP	151.3046	49.52946	0.33	ILMN_176088	RFXAP	13
SERAC1	909.67	297.8216	0.33	ILMN_7584	SERAC1	6
CAPN1	387.8633	127.0707	0.33	ILMN_10477	CAPN1	11
SYNJ1	1330.379	435.9238	0.33	ILMN_8265	SYNJ1	21
NQO2	1131.943	371.1605	0.33	ILMN_26249	NQO2	
DOCK3	510.1911	167.5049	0.33	ILMN_19532	DOCK3	3
ZMAT1	662.0095	217.4579	0.33	ILMN_9378	ZMAT1	X
PLXNB3	265.1874	87.11626	0.33	ILMN_12467	PLXNB3	X
S100A7L2	6.522082	2.142658	0.33	ILMN_167547	S100A7L2	1
IQUB	445.0177	146.2262	0.33	ILMN_28716	IQUB	7
S100Z	13.35705	4.389611	0.33	ILMN_16143	S100Z	5
LOC283392	21.88267	7.197014	0.33	ILMN_23408	LOC283392	12
DNAH12L	224.549	73.87873	0.33	ILMN_43250	DNAH12L	3
MTA1	327.2812	107.8714	0.33	ILMN_7788	MTA1	14
ROR2	1450.243	478.1733	0.33	ILMN_22834	ROR2	9
LZTS2	559.9425	184.6856	0.33	ILMN_12748	LZTS2	10
APOL1	75.28822	24.87243	0.33	ILMN_4231	APOL1	22
DENND5B	606.2612	200.3034	0.33	ILMN_24291	DENND5B	12
ZPBP2	90.51827	29.92163	0.33	ILMN_138844	ZPBP2	17
ZNRF2	969.8044	321.0394	0.33	ILMN_964	ZNRF2	7
FAM123A	10.01034	3.315854	0.33	ILMN_26739	FAM123A	13
C14orf37	43.05121	14.26536	0.33	ILMN_3614	C14ORF37	14
CELSR3	3547.145	1176.103	0.33	ILMN_12021	CELSR3	3
PDGFC	1261.048	419.0082	0.33	ILMN_13763	PDGFC	4
CAP2	1794.226	596.7059	0.33	ILMN_27367	CAP2	6
NFYA	217.3147	72.29575	0.33	ILMN_10390	NFYA	6
FLJ27465	288.4795	96.07603	0.33	ILMN_137188	FLJ27465	15
CDKN2B	2584.211	860.7133	0.33	ILMN_183997	CDKN2B	9
EGR3	186.4292	62.09597	0.33	ILMN_15851	EGR3	8
NBEAL1	311.9442	103.9051	0.33	ILMN_7683	NBEAL1	2
IER5L	2391.142	796.7241	0.33	ILMN_16273	IER5L	9
TTL	510.4509	170.0934	0.33	ILMN_14027	TTL	2
PTGFRN	758.1554	252.6964	0.33	ILMN_175316	PTGFRN	1
ANKRD22	17.2109	51.71918	3.01	ILMN_7804	ANKRD22	10
TRIM50	14.38383	43.22463	3.01	ILMN_6662	TRIM50	7
POLR2L	431.7923	1298.788	3.01	ILMN_13427	POLR2L	11
ZNF419	283.3271	853.0358	3.01	ILMN_2849	ZNF419	19
TPM1	447.9689	1349.291	3.01	ILMN_8136	TPM1	15
KRTAP10-2	1.241375	3.743516	3.02	ILMN_139042	KRTAP10-2	21
SUPT3H	276.347	834.1354	3.02	ILMN_4557	SUPT3H	6
KIF25	29.24875	88.30277	3.02	ILMN_25343	KIF25	6

LRRC29	117.3484	354.373	3.02	ILMN_11967	LRRC29	16
MOGAT3	14.00944	42.31306	3.02	ILMN_29070	MOGAT3	7
ATOH1	6.795612	20.52743	3.02	ILMN_19379	ATOH1	4
DAK	65.38979	197.7267	3.02	ILMN_3575	DAK	11
NFATC4	19.61579	59.31536	3.02	ILMN_23517	NFATC4	14
TROAP	350.6808	1060.641	3.02	ILMN_13141	TROAP	12
ERC2	8.166249	24.76944	3.03	ILMN_17447	ERC2	3
SOSTDC1	25.27429	76.71832	3.04	ILMN_24005	SOSTDC1	7
IL12RB2	2.503549	7.60402	3.04	ILMN_5787	IL12RB2	1
RFX3	112.4561	341.6617	3.04	ILMN_23277	RFX3	9
CENPL	33.76624	102.5947	3.04	ILMN_21203	CENPL	1
ERBB3	618.9373	1883.156	3.04	ILMN_8157	ERBB3	12
TRPV6	120.2604	365.9528	3.04	ILMN_29216	TRPV6	7
WNT9B	4.048599	12.32492	3.04	ILMN_22095	WNT9B	17
GPAA1	828.1827	2522.733	3.05	ILMN_138828	GPAA1	8
MYO1C	367.5122	1121.263	3.05	ILMN_10709	MYO1C	17
D2HGDH	578.8497	1766.068	3.05	ILMN_18076	D2HGDH	2
SLC7A4	3.2433	9.89743	3.05	ILMN_22167	SLC7A4	22
CABC1	1928.356	5886.255	3.05	ILMN_17033	CABC1	1
APEH	919.8406	2810.008	3.05	ILMN_27694	APEH	3
LIN7B	111.2153	339.9927	3.06	ILMN_29543	LIN7B	19
DEF6	433.7241	1326.026	3.06	ILMN_27305	DEF6	6
PDGFRL	1596.872	4882.354	3.06	ILMN_8522	PDGFRL	8
LIN37	123.0491	376.3631	3.06	ILMN_11408	LIN37	19
ZNF34	35.42216	108.3683	3.06	ILMN_7366	ZNF34	8
FAM119A	118.3079	362.2935	3.06	ILMN_15705	FAM119A	2
DDX54	704.8748	2160.279	3.06	ILMN_28699	DDX54	12
QTRT1	211.7039	648.852	3.06	ILMN_10992	QTRT1	19
KLF10	339.305	1040.721	3.07	ILMN_2466	KLF10	8
PABPN1	611.2049	1875.616	3.07	ILMN_29660	PABPN1	14
DDX27	147.1517	452.3402	3.07	ILMN_20732	DDX27	20
KHK	134.9386	414.8523	3.07	ILMN_29655	KHK	2
HIST1H3F	43.12535	132.7154	3.08	ILMN_8533	HIST1H3F	6
ZC3H3	2934.767	9032.476	3.08	ILMN_19435	ZC3H3	8
BFSP2	59.82139	184.2128	3.08	ILMN_23075	BFSP2	3
RRAGC	61.01812	188.0089	3.08	ILMN_20301	RRAGC	1
IQSEC3	9.223082	28.45261	3.08	ILMN_7732	IQSEC3	12
LENG1	43.48221	134.2487	3.09	ILMN_25460	LENG1	19
UFSP1	93.14899	287.7102	3.09	ILMN_27699	UFSP1	7
ARHGEF17	299.4933	926.9198	3.09	ILMN_11064	ARHGEF17	11
FAM40B	42.11718	130.3807	3.1	ILMN_18452	FAM40B	7
TNFRSF13C	70.49934	218.4726	3.1	ILMN_22155	TNFRSF13C	22
ABHD14B	2450.755	7596.29	3.1	ILMN_24812	ABHD14B	3
UBR1	56.24582	174.5659	3.1	ILMN_137462	UBR1	15
EPHA4	71.21165	221.1953	3.11	ILMN_21869	EPHA4	2
EPHX2	269.5971	838.2358	3.11	ILMN_22751	EPHX2	8
ACVR1B	628.2556	1954.615	3.11	ILMN_14555	ACVR1B	12

F7	14.62788	45.53284	3.11	ILMN_4095	F7	13
SLC7A10	1.956242	6.092691	3.11	ILMN_6485	SLC7A10	19
ACAA1	932.5142	2907.314	3.12	ILMN_30044	ACAA1	3
NAG18	52.33554	163.19	3.12	ILMN_28653	NAG18	7
PTCD3	56.51374	176.2645	3.12	ILMN_3864	PTCD3	2
PCDH10	16.12106	50.31933	3.12	ILMN_26910	PCDH10	4
ALPPL2	21.12227	65.93996	3.12	ILMN_23145	ALPPL2	2
PVRL1	59.73098	187.0404	3.13	ILMN_12109	PVRL1	11
PNPT1	727.1581	2277.589	3.13	ILMN_22316	PNPT1	2
KIF2A	282.0296	883.424	3.13	ILMN_8115	KIF2A	5
KISS1	98.29049	308.6904	3.14	ILMN_21021	KISS1	1
POU2F1	9.23735	29.0258	3.14	ILMN_17030	POU2F1	1
FLJ45224	9.436368	29.69369	3.15	ILMN_6474	FLJ45224	9
LYPLA2P1	14.89133	46.8665	3.15	ILMN_5913	LYPLA2P1	6
OR7C2	3.224847	10.15234	3.15	ILMN_16831	OR7C2	19
SBDSP	247.5858	779.7941	3.15	ILMN_12233	SBDSP	7
GRIN2D	28.60813	90.17795	3.15	ILMN_14699	GRIN2D	19
C17orf59	186.2276	587.5538	3.16	ILMN_4989	C17ORF59	17
C2orf65	667.5372	2107.037	3.16	ILMN_28342	C2ORF65	2
C14orf131	997.2194	3152.287	3.16	ILMN_8908	C14ORF131	14
MED24	2795.741	8837.928	3.16	ILMN_168341	MED24	17
EXT1	1146.774	3632.264	3.17	ILMN_18298	EXT1	8
LOC653319	38.9208	123.3633	3.17	ILMN_167940	LOC653319	16
INADL	148.0737	469.9416	3.17	ILMN_19026	INADL	1
C11orf9	601.8062	1910.334	3.17	ILMN_22426	C11ORF9	11
C1orf125	51.55135	163.7007	3.18	ILMN_8437	C1ORF125	1
POLG	408.5887	1299.075	3.18	ILMN_13641	POLG	15
CREB3L1	53.35703	169.9189	3.18	ILMN_23950	CREB3L1	11
TCTN1	1139.254	3628.477	3.18	ILMN_9097	TCTN1	12
LEPREL2	323.95	1032.524	3.19	ILMN_2115	LEPREL2	12
CUGBP2	24.36538	77.66779	3.19	ILMN_21057	CUGBP2	10
CHRNB1	741.3866	2363.459	3.19	ILMN_12391	CHRNB1	17
ZFHX3	890.0294	2838.913	3.19	ILMN_15864	ZFHX3	16
C2orf48	31.61394	100.8515	3.19	ILMN_5452	C2ORF48	2
MMACHC	2111.453	6741.039	3.19	ILMN_17978	MMACHC	1
USP45	21.95838	70.1049	3.19	ILMN_180741	USP45	6
LOC441120	21.72835	69.37086	3.19	ILMN_164288	LOC441120	5
MTL5	166.0489	530.2662	3.19	ILMN_46873	MTL5	11
C7orf60	3.3996	10.85796	3.19	ILMN_32648	C7ORF60	7
NPPC	9.348625	29.88358	3.2	ILMN_11431	NPPC	2
KL	8.579117	27.46936	3.2	ILMN_179305	KL	13
STX18	10.30903	33.01116	3.2	ILMN_1325	STX18	4
C1QTNF8	7.036222	22.54399	3.2	ILMN_24685	C1QTNF8	16
OR8D1	18.18303	58.275	3.2	ILMN_11387	OR8D1	11
MYO6	158.6499	510.3016	3.22	ILMN_27832	MYO6	6
DHODH	88.02311	283.4465	3.22	ILMN_875	DHODH	16
POPDC2	1.892816	6.097792	3.22	ILMN_17743	POPDC2	3

COX11P	93.9571	302.8679	3.22	ILMN_3313	COX11P	6
SPSB1	673.8431	2172.716	3.22	ILMN_12158	SPSB1	1
C19orf4	11.14137	35.93039	3.22	ILMN_2040	C19ORF4	19
C9orf165	23.36817	75.36971	3.23	ILMN_13417	C9ORF165	9
PDCD4	2602.232	8404.154	3.23	ILMN_12916	PDCD4	10
HIC2	1191.751	3858.221	3.24	ILMN_22371	HIC2	22
SNF1LK2	216.7456	702.0499	3.24	ILMN_17085	SNF1LK2	11
RNF165	877.3192	2842.032	3.24	ILMN_14516	RNF165	18
NDUFA12	2497.395	8092.352	3.24	ILMN_26981	NDUFA12	12
POLR3D	180.9358	586.8646	3.24	ILMN_4403	POLR3D	8
FAM38A	1882.727	6108.899	3.24	ILMN_3694	FAM38A	16
AKR1CL2	47.35221	154.0124	3.25	ILMN_8236	AKR1CL2	10
TNFRSF1A	424.2407	1380.543	3.25	ILMN_6742	TNFRSF1A	12
RNASEH2A	141.0441	460.6418	3.27	ILMN_20484	RNASEH2A	19
OR1A2	5.721334	18.68673	3.27	ILMN_15428	OR1A2	17
FAM161B	41.65106	136.0922	3.27	ILMN_13309	FAM161B	14
SMTNL2	445.1904	1455.738	3.27	ILMN_24119	SMTNL2	17
ORAI1	263.299	863.9491	3.28	ILMN_29870	ORAI1	12
LRRC43	7.062291	23.20832	3.29	ILMN_969	LRRC43	12
FLJ39827	1222.426	4019.416	3.29	ILMN_19358	FLJ39827	X
KIAA1543	207.4186	682.0417	3.29	ILMN_171009	KIAA1543	
TSGA10	264.0217	868.9721	3.29	ILMN_16441	TSGA10	2
DDX19A	1196.594	3941.295	3.29	ILMN_10415	DDX19A	16
ANO5	57.96205	191.1267	3.3	ILMN_27431	ANO5	11
TAS2R3	5.776893	19.07454	3.3	ILMN_26786	TAS2R3	7
RASD1	0.926587	3.06611	3.31	ILMN_13561	RASD1	17
C4orf37	7.151282	23.67056	3.31	ILMN_175971	C4ORF37	4
C9orf140	1744.55	5776.573	3.31	ILMN_24887	C9ORF140	9
GNG8	5.943411	19.69007	3.31	ILMN_25463	GNG8	19
HOXC8	837.8594	2775.889	3.31	ILMN_8158	HOXC8	12
CKAP4	74.02055	245.262	3.31	ILMN_4432	CKAP4	12
FLJ21865	1275.853	4229.377	3.31	ILMN_20558	FLJ21865	17
SH3RF2	138.4976	459.2608	3.32	ILMN_18847	SH3RF2	5
ZNF485	32.44663	107.61	3.32	ILMN_17191	ZNF485	10
ZNF417	246.6297	819.3362	3.32	ILMN_29570	ZNF417	19
HIVEP1	613.5472	2039.827	3.32	ILMN_6517	HIVEP1	6
C10orf84	50.01421	166.3223	3.33	ILMN_12385	C10ORF84	10
NAALADL1	10.72416	35.66464	3.33	ILMN_15126	NAALADL1	11
PLEKHG4	798.0486	2656.091	3.33	ILMN_11658	PLEKHG4	16
HOXA13	2059.077	6853.601	3.33	ILMN_138904	HOXA13	
CPLX1	925.4587	3089.917	3.34	ILMN_30247	CPLX1	4
CTRL	39.36051	131.4925	3.34	ILMN_21415	CTRL	16
NAALADL2	25.40187	84.88142	3.34	ILMN_27251	NAALADL2	3
FAM109A	314.9662	1052.575	3.34	ILMN_26928	FAM109A	12
FKSG24	335.1874	1121.099	3.34	ILMN_6323	FKSG24	19
CCDC113	9.392274	31.43193	3.35	ILMN_28091	CCDC113	16
ANG	1361.554	4556.812	3.35	ILMN_13716	ANG	14

CCDC67	2.547567	8.541594	3.35	ILMN_29506	CCDC67	11
SLC17A1	0.511401	1.716653	3.36	ILMN_13403	SLC17A1	
ANO9	56.49909	189.6862	3.36	ILMN_17016	ANO9	11
LOC16438(	8.779597	29.54819	3.37	ILMN_2576	LOC164380	20
RAMP1	2.282148	7.682772	3.37	ILMN_16245	RAMP1	2
SRFBP1	313.746	1056.534	3.37	ILMN_15262	SRFBP1	5
NBL1	2373.534	7998.235	3.37	ILMN_20462	NBL1	1
PCTK3	95.86134	323.1292	3.37	ILMN_20580	PCTK3	1
EP300	436.7337	1472.273	3.37	ILMN_8705	EP300	22
SCAMP3	2058.831	6942.334	3.37	ILMN_166813	SCAMP3	1
MATN1	52.67599	177.776	3.37	ILMN_29983	MATN1	1
ZNF513	6.119962	20.65765	3.38	ILMN_16415	ZNF513	2
LMBRD2	32.59541	110.1534	3.38	ILMN_25092	LMBRD2	5
PHF19	184.6009	625.0964	3.39	ILMN_21027	PHF19	9
RBMX	719.431	2436.673	3.39	ILMN_11671	RBMX	X
USP30	355.2164	1203.465	3.39	ILMN_5598	USP30	12
DLK2	204.6604	694.5478	3.39	ILMN_6101	DLK2	6
SEC61A1	1987.442	6747.568	3.4	ILMN_9397	SEC61A1	3
TRMT1	213.3	724.6874	3.4	ILMN_6438	TRMT1	19
MGC5139	29.40373	99.93517	3.4	ILMN_39523	MGC5139	12
TAZ	13.03746	44.31382	3.4	ILMN_29643	TAZ	X
MX2	859.8717	2922.801	3.4	ILMN_20722	MX2	21
C5orf25	207.1541	704.8224	3.4	ILMN_16903	C5ORF25	5
EXDL1	7.920867	26.96007	3.4	ILMN_25846	EXDL1	15
GLTSCR1	487.7085	1661.608	3.41	ILMN_18273	GLTSCR1	19
LMBR1L	3.750415	12.78417	3.41	ILMN_27914	LMBR1L	12
KCNE3	30.32221	103.4052	3.41	ILMN_22298	KCNE3	11
CTF1	349.2078	1195.652	3.42	ILMN_13986	CTF1	16
ADCY6	477.673	1636.812	3.43	ILMN_18413	ADCY6	12
VPS26B	142.3946	488.1922	3.43	ILMN_138811	VPS26B	11
ACSM3	488.6347	1675.402	3.43	ILMN_10620	ACSM3	16
LRDD	73.79553	253.1278	3.43	ILMN_1008	LRDD	11
LSM11	233.9759	803.9689	3.44	ILMN_29000	LSM11	5
DQX1	54.58456	187.6567	3.44	ILMN_26510	DQX1	2
GCS1	574.4976	1979.721	3.45	ILMN_5900	GCS1	2
SUV39H2	2.118767	7.305668	3.45	ILMN_29467	SUV39H2	10
CENTG3	4.392529	15.15093	3.45	ILMN_29137	CENTG3	7
HIST1H3B	165.6355	571.6357	3.45	ILMN_30162	HIST1H3B	6
C1orf100	10.48558	36.20703	3.45	ILMN_8320	C1ORF100	1
CDC6	303.0883	1048.339	3.46	ILMN_21854	CDC6	17
BARD1	1400.065	4845.261	3.46	ILMN_1301	BARD1	2
NUMA1	815.8518	2824.52	3.46	ILMN_25058	NUMA1	11
TMEM39B	117.6821	407.6055	3.46	ILMN_1056	TMEM39B	1
GPR161	9.690607	33.56841	3.46	ILMN_22837	GPR161	1
PDDC1	646.2604	2239.39	3.47	ILMN_26571	PDDC1	11
FAM167B	3.525361	12.22052	3.47	ILMN_5462	FAM167B	1
NEIL1	30.9442	107.2728	3.47	ILMN_18060	NEIL1	15

KIAA0415	259.5233	899.9063	3.47	ILMN_29786	KIAA0415	7
PSTK	273.8299	949.9447	3.47	ILMN_27894	PSTK	10
FUT3	385.2847	1337.832	3.47	ILMN_10592	FUT3	19
SLC25A34	31.33223	108.8168	3.47	ILMN_6699	SLC25A34	1
FARSB	26.24856	91.18336	3.47	ILMN_170745	FARSB	2
BEGAIN	6.034863	20.97458	3.48	ILMN_10503	BEGAIN	14
KIRREL	5.477616	19.03793	3.48	ILMN_12756	KIRREL	1
WDR81	2.055909	7.148749	3.48	ILMN_25557	WDR81	17
NRG1	18.38357	63.93846	3.48	ILMN_29179	NRG1	8
GPR146	6.320877	22.03055	3.49	ILMN_14481	GPR146	7
TJP2	37.73399	131.5547	3.49	ILMN_3071	TJP2	9
MTERFD2	9.175845	31.99726	3.49	ILMN_4915	MTERFD2	2
CYC1	1879.261	6554.022	3.49	ILMN_13263	CYC1	8
PKD1	367.1369	1280.499	3.49	ILMN_177255	PKD1	16
DNAJC4	10.56869	36.95071	3.5	ILMN_17960	DNAJC4	11
BMPR1A	7.618145	26.64039	3.5	ILMN_170500	BMPR1A	10
RHOT2	642.2862	2247.584	3.5	ILMN_21353	RHOT2	16
ESRRAP2	16.46076	57.62595	3.5	ILMN_21596	ESRRAP2	13
ARC	61.59136	215.8315	3.5	ILMN_28586	ARC	8
ADCY7	20.47204	71.74236	3.5	ILMN_24596	ADCY7	16
CNNM1	69.47968	243.5897	3.51	ILMN_9103	CNNM1	10
VMAC	4.794238	16.81194	3.51	ILMN_138448	VMAC	19
ATXN7L2	54.66185	191.6844	3.51	ILMN_6796	ATXN7L2	1
ZDHHC12	635.9359	2232.16	3.51	ILMN_27278	ZDHHC12	9
C4orf23	69.99364	245.955	3.51	ILMN_20913	C4ORF23	4
GEM	192.4288	676.2432	3.51	ILMN_20370	GEM	8
JUP	609.487	2143.87	3.52	ILMN_2607	JUP	17
VISA	256.7426	903.6746	3.52	ILMN_22608	VISA	20
RRH	3.06611	10.80422	3.52	ILMN_6280	RRH	4
HIST1H2BK	466.4862	1644.364	3.53	ILMN_17974	HIST1H2BK	6
DCI	1091.473	3848.876	3.53	ILMN_15001	DCI	16
RDM1	54.25038	191.3797	3.53	ILMN_14236	RDM1	17
IL17RD	51.97269	183.5433	3.53	ILMN_7284	IL17RD	3
TM4SF20	41.77297	147.7352	3.54	ILMN_26019	TM4SF20	2
GCM1	29.88358	105.785	3.54	ILMN_9230	GCM1	6
BBC3	29.21671	103.6014	3.55	ILMN_6992	BBC3	19
MYRIP	498.2947	1767.062	3.55	ILMN_28637	MYRIP	3
SAMD5	1320.384	4687.331	3.55	ILMN_18130	SAMD5	6
CGB5	156.2445	555.4443	3.55	ILMN_17361	CGB5	19
GEMIN8	225.7919	802.7314	3.56	ILMN_164552	GEMIN8	X
ZCCHC3	340.2226	1209.771	3.56	ILMN_28604	ZCCHC3	20
OSR1	566.7665	2018.759	3.56	ILMN_5789	OSR1	2
CLEC3B	2.297722	8.187509	3.56	ILMN_1287	CLEC3B	3
C6orf134	52.90128	188.5041	3.56	ILMN_7508	C6ORF134	6
HRB	28.3587	101.1154	3.57	ILMN_168342	HRB	2
CGB2	51.51281	183.775	3.57	ILMN_22770	CGB2	19
ZNF540	25.15396	90.07614	3.58	ILMN_18312	ZNF540	19

RNF183	6.069172	21.73559	3.58	ILMN_20385	RNF183	9
CYB5R2	66.47866	238.3159	3.58	ILMN_19891	CYB5R2	11
SCAMP2	222.1007	796.4694	3.59	ILMN_20277	SCAMP2	15
DISP2	348.5487	1250.065	3.59	ILMN_6654	DISP2	15
TBL2	462.5835	1659.588	3.59	ILMN_136934	TBL2	7
ARHGDIG	4.454668	16.00169	3.59	ILMN_176091	ARHGDIG	16
ZHX2	229.0618	822.8458	3.59	ILMN_176506	ZHX2	8
SOLH	225.1412	808.933	3.59	ILMN_593	SOLH	16
TAP1	3187.226	11453.65	3.59	ILMN_17479	TAP1	6
OR4C45	3.22919	11.63694	3.6	ILMN_1481	OR4C45	
ERP27	1150.826	4148.418	3.6	ILMN_26083	ERP27	12
YIF1B	198.28	715.3267	3.61	ILMN_41511	YIF1B	19
ZNF528	49.98753	180.3786	3.61	ILMN_13895	ZNF528	19
FLJ33360	11.5815	41.81306	3.61	ILMN_21809	FLJ33360	5
OR7E91P	111.8239	403.9699	3.61	ILMN_14923	OR7E91P	2
LAMB1	355.476	1284.708	3.61	ILMN_182874	LAMB1	7
AAA1	42.23874	152.6881	3.61	ILMN_21956	AAA1	7
C3orf34	100.3387	363.4125	3.62	ILMN_21898	C3ORF34	3
HRC	9.196841	33.36719	3.63	ILMN_23773	HRC	19
ZNF396	48.9158	177.7003	3.63	ILMN_7285	ZNF396	18
CRTC1	69.03157	250.847	3.63	ILMN_27180	CRTC1	19
EFHD1	696.3366	2530.374	3.63	ILMN_14358	EFHD1	2
ZNF474	23.03816	83.7702	3.64	ILMN_26423	ZNF474	5
FOXO1	767.1704	2792.373	3.64	ILMN_5656	FOXO1	13
RIBC1	20.16576	73.41258	3.64	ILMN_12204	RIBC1	X
GCHFR	1655.781	6035.978	3.65	ILMN_22818	GCHFR	15
CD72	39.45685	143.8794	3.65	ILMN_20590	CD72	9
CLIP3	55.15268	201.2383	3.65	ILMN_26693	CLIP3	19
TIGD6	33.07315	120.7334	3.65	ILMN_11472	TIGD6	5
SETD1A	481.5662	1759.276	3.65	ILMN_24764	SETD1A	16
GRB7	210.1767	768.1182	3.65	ILMN_3837	GRB7	17
TCF7	19.43297	71.17588	3.66	ILMN_19540	TCF7	5
WDR93	13.97384	51.29802	3.67	ILMN_18593	WDR93	15
FLJ43860	17.26577	63.43901	3.67	ILMN_24679	FLJ43860	8
GAGE5	21.16813	77.7887	3.67	ILMN_9557	GAGE5	X
FBXW2	975.4987	3593.272	3.68	ILMN_28252	FBXW2	9
TSNARE1	3.632087	13.38447	3.69	ILMN_10886	TSNARE1	8
SCAMP4	49.13854	181.4406	3.69	ILMN_17351	SCAMP4	19
PIP5KL1	11.56064	42.7228	3.7	ILMN_4102	PIP5KL1	9
MMP3	13.86701	51.26419	3.7	ILMN_1300	MMP3	11
AVPR1A	17.79935	65.87111	3.7	ILMN_25328	AVPR1A	12
PPARA	117.229	434.3808	3.71	ILMN_7270	PPARA	22
HOXA4	230.2292	853.7422	3.71	ILMN_138508	HOXA4	7
FMNL3	135.2808	501.6602	3.71	ILMN_4212	FMNL3	12
ARID5B	1826.611	6776.086	3.71	ILMN_17477	ARID5B	10
C12orf4	201.355	747.6016	3.71	ILMN_9236	C12ORF4	12
SLC35E1	61.22244	227.3574	3.71	ILMN_23168	SLC35E1	19

COX7A1	4.163218	15.4693	3.72	ILMN_5679	COX7A1	19
ADAMTS13	2.356745	8.763124	3.72	ILMN_4294	ADAMTS13	9
KIAA1161	22.28658	82.8992	3.72	ILMN_6372	KIAA1161	9
HSPA12B	11.99729	44.66234	3.72	ILMN_10463	HSPA12B	20
KLHL17	15.9794	59.50565	3.72	ILMN_30075	KLHL17	1
P2RY11	141.6885	528.4726	3.73	ILMN_12237	P2RY11	19
CYHR1	1298.358	4842.701	3.73	ILMN_6065	CYHR1	8
SERINC2	908.3979	3390.689	3.73	ILMN_3596	SERINC2	1
ITGB7	12.8487	47.969	3.73	ILMN_26232	ITGB7	12
FAM132A	155.9536	582.2381	3.73	ILMN_44524	FAM132A	1
HSD17B6	932.2628	3483.837	3.74	ILMN_26397	HSD17B6	12
TMEM149	76.85593	287.2092	3.74	ILMN_5343	TMEM149	19
ZNF547	0.49562	1.852411	3.74	ILMN_17087	ZNF547	19
CDKL4	1.185057	4.431665	3.74	ILMN_1746	CDKL4	2
GRM8	14.97173	55.99206	3.74	ILMN_11549	GRM8	7
NRF1	25.91525	96.92704	3.74	ILMN_9890	NRF1	7
OPRK1	10.17052	38.06311	3.74	ILMN_18793	OPRK1	8
PDZD2	12.63215	47.35221	3.75	ILMN_137237	PDZD2	5
DAXX	254.1386	952.9451	3.75	ILMN_19278	DAXX	6
GJB5	0.744216	2.792842	3.75	ILMN_28687	GJB5	1
TMEM200B	15.27673	57.33623	3.75	ILMN_15308	TMEM200B	1
MREG	24.05363	90.30173	3.75	ILMN_11783	MREG	2
OTOP2	10.53874	39.58538	3.76	ILMN_6423	OTOP2	17
PCDH1	45.31606	170.3107	3.76	ILMN_9390	PCDH1	5
EPHA1	1223.673	4606.285	3.76	ILMN_26326	EPHA1	7
LETMD1	697.8347	2627.241	3.76	ILMN_642	LETMD1	12
MAPK3	978.9944	3686.204	3.77	ILMN_11845	MAPK3	
FAM129C	1.69466	6.38623	3.77	ILMN_26827	FAM129C	19
C11orf88	0.641986	2.420474	3.77	ILMN_2210	C11ORF88	11
CCL21	9.672173	36.53858	3.78	ILMN_4876	CCL21	9
SLC16A14	48.1101	181.7632	3.78	ILMN_5489	SLC16A14	2
DKK2	21.02098	79.42422	3.78	ILMN_20007	DKK2	4
LRRC32	40.82562	154.2768	3.78	ILMN_3568	LRRC32	11
LNPEP	428.4248	1619.124	3.78	ILMN_15141	LNPEP	5
NINJ2	129.55	489.9471	3.78	ILMN_6659	NINJ2	12
HYDIN	56.97466	215.9341	3.79	ILMN_3739	HYDIN	16
SGPL1	489.3811	1856.68	3.79	ILMN_5084	SGPL1	10
CHST10	4.379795	16.63012	3.8	ILMN_8537	CHST10	2
RNF212	2.59975	9.886542	3.8	ILMN_25052	RNF212	4
PCDH19	180.8566	688.1447	3.8	ILMN_20959	PCDH19	X
BOK	481.9624	1838.772	3.82	ILMN_19886	BOK	2
CYP4F2	27.18567	103.7312	3.82	ILMN_18195	CYP4F2	19
PKN3	413.3105	1578.711	3.82	ILMN_30257	PKN3	9
TESK2	126.7078	484.1997	3.82	ILMN_20025	TESK2	1
TNFAIP2	504.1264	1926.567	3.82	ILMN_16372	TNFAIP2	14
USP46	5.434803	20.79074	3.83	ILMN_2873	USP46	4
CLDN16	25.18254	96.40708	3.83	ILMN_166090	CLDN16	3

CLN3	287.2387	1100.259	3.83	ILMN_24228	CLN3	16
MYD88	352.4999	1350.276	3.83	ILMN_15762	MYD88	3
STYXL1	1384.722	5306.879	3.83	ILMN_5068	STYXL1	7
TBL3	274.0806	1050.756	3.83	ILMN_2982	TBL3	16
HSPC171	1963.668	7533.818	3.84	ILMN_20854	HSPC171	
NUAK2	815.3438	3132.232	3.84	ILMN_13538	NUAK2	1
SLC7A5P1	54.73386	210.6832	3.85	ILMN_7550	SLC7A5P1	16
MYOM3	889.0652	3423.192	3.85	ILMN_4488	MYOM3	1
P2RX4	90.4572	348.5487	3.85	ILMN_23982	P2RX4	12
SPAG4	9.850349	37.95852	3.85	ILMN_15424	SPAG4	20
BCAR1	38.70445	149.1648	3.85	ILMN_2703	BCAR1	16
MMAB	78.8821	304.2996	3.86	ILMN_5798	MMAB	12
CYP2D6	11.3455	43.77496	3.86	ILMN_27062	CYP2D6	22
ULBP3	10.07132	38.87709	3.86	ILMN_27663	ULBP3	6
MIER3	659.9073	2550.426	3.86	ILMN_20832	MIER3	5
PRR5	368.0455	1424.509	3.87	ILMN_5069	PRR5	22
AMPD2	5.698205	22.0799	3.87	ILMN_20710	AMPD2	1
LRP1	19.4432	75.43362	3.88	ILMN_22753	LRP1	12
TUBAL3	1204.311	4679.665	3.89	ILMN_13951	TUBAL3	10
DPM3	636.1476	2472.351	3.89	ILMN_29764	DPM3	1
C1orf88	9.234753	35.9174	3.89	ILMN_15462	C1ORF88	1
SH3TC2	5.264086	20.48601	3.89	ILMN_26820	SH3TC2	5
TNFRSF18	264.4725	1029.392	3.89	ILMN_22947	TNFRSF18	1
MT1B	5.734165	22.32964	3.89	ILMN_20450	MT1B	16
TTPA	18.95438	73.92627	3.9	ILMN_911	TTPA	8
AAAS	160	624.3619	3.9	ILMN_22994	AAAS	12
EIF5A	169.1969	660.7839	3.91	ILMN_26100	EIF5A	17
ARMC6	13.5352	52.9049	3.91	ILMN_8868	ARMC6	19
PCK2	585.4476	2290.238	3.91	ILMN_18787	PCK2	14
C12orf28	22.85149	89.41628	3.91	ILMN_1950	C12ORF28	12
FAM124A	79.38186	310.9338	3.92	ILMN_18616	FAM124A	13
MAP9	1.716653	6.742875	3.93	ILMN_27334	MAP9	4
TASP1	636.6725	2505.12	3.93	ILMN_4752	TASP1	20
MAP3K14	42.55124	167.6684	3.94	ILMN_6407	MAP3K14	17
DPP6	1.531884	6.041441	3.94	ILMN_137561	DPP6	7
KARS	2237.546	8825.053	3.94	ILMN_15158	KARS	16
TFAMP1	518.8279	2049.006	3.95	ILMN_3649	TFAMP1	7
ZNF767	218.2547	862.3199	3.95	ILMN_28810	ZNF767	7
LOC401010	22.32134	88.24169	3.95	ILMN_16998	LOC401010	2
EIF5	386.1202	1528.68	3.96	ILMN_17164	EIF5	14
TAF6	138.0461	546.8677	3.96	ILMN_3330	TAF6	7
RAD51L1	24.63883	97.70708	3.97	ILMN_15278	RAD51L1	14
RAB5C	171.65	681.5619	3.97	ILMN_14169	RAB5C	17
PHLPP	1188.809	4721.204	3.97	ILMN_2868	PHLPP	18
SGPP2	55.35651	219.9262	3.97	ILMN_4626	SGPP2	2
TMEM222	87.20263	346.4946	3.97	ILMN_4563	TMEM222	1
APOA1	62.85355	249.8144	3.97	ILMN_23504	APOA1	11

SLC5A1	388.6509	1548.001	3.98	ILMN_24451	SLC5A1	22
CHCHD1	817.8678	3257.702	3.98	ILMN_23494	CHCHD1	10
ZNF692	1256.385	5005.582	3.98	ILMN_22649	ZNF692	1
CYB5A	1097.868	4379.351	3.99	ILMN_24700	CYB5A	18
MB	33.99193	135.6309	3.99	ILMN_5316	MB	22
GDPD3	166.8067	665.6097	3.99	ILMN_9502	GDPD3	16
CDADC1	9.550813	38.14057	3.99	ILMN_19906	CDADC1	13
KRI1	150.8292	602.4358	3.99	ILMN_2432	KRI1	19
PCDHGA12	18.89701	75.6625	4	ILMN_9233	PCDHGA12	5
UCK2	771.3541	3089.092	4	ILMN_23283	UCK2	1
TMEM27	273.0483	1094.151	4.01	ILMN_6413	TMEM27	X
ACOX3	15.29841	61.51751	4.02	ILMN_4422	ACOX3	4
KIAA0195	492.2682	1980.97	4.02	ILMN_16248	KIAA0195	17
IL20RB	8.149994	32.8079	4.03	ILMN_26767	IL20RB	3
C22orf29	806.3181	3252.048	4.03	ILMN_4587	C22ORF29	22
TTC7B	0.991297	3.998276	4.03	ILMN_25827	TTC7B	14
GYPC	2.530989	10.22701	4.04	ILMN_16212	GYPC	2
PYCARD	8.786082	35.54224	4.05	ILMN_1146	PYCARD	16
PANK3	61.91558	250.7059	4.05	ILMN_4465	PANK3	5
TCF3	334.1345	1354.865	4.05	ILMN_5571	TCF3	19
LOC728242	10.79667	43.84966	4.06	ILMN_165057	LOC728242	X
TKTL1	192.002	780.5208	4.07	ILMN_23315	TKTL1	X
NUDT16	294.601	1198.665	4.07	ILMN_16023	NUDT16	3
CREB3L3	19.75197	80.39902	4.07	ILMN_7660	CREB3L3	19
UNC13A	3.563371	14.51678	4.07	ILMN_170667	UNC13A	19
KRT18	31.1862	127.1344	4.08	ILMN_22651	KRT18	12
COX19	27.56134	112.3769	4.08	ILMN_15655	COX19	7
BAT5	1890.468	7709.522	4.08	ILMN_25579	BAT5	6
MBOAT2	20.72191	84.6487	4.08	ILMN_139151	MBOAT2	
KLF13	228.7233	934.6674	4.09	ILMN_16226	KLF13	15
CYP2B6	108.2688	442.4772	4.09	ILMN_182193	CYP2B6	19
TMEM55B	992.692	4058.67	4.09	ILMN_8747	TMEM55B	14
SLC39A12	54.13982	221.5122	4.09	ILMN_7927	SLC39A12	10
FLT3LG	150.2429	617.0941	4.11	ILMN_4754	FLT3LG	19
FZD4	353.7018	1453.246	4.11	ILMN_13859	FZD4	11
KRTDAP	46.31801	190.3181	4.11	ILMN_1241	KRTDAP	19
USP21	97.82396	402.4171	4.11	ILMN_18019	USP21	1
ZYX	204.9299	844.8109	4.12	ILMN_2137	ZYX	7
FAM105A	11.05429	45.65711	4.13	ILMN_10775	FAM105A	5
FOLR1	535.2554	2210.832	4.13	ILMN_17047	FOLR1	11
SH2D3A	446.7231	1847.295	4.14	ILMN_26382	SH2D3A	19
PIWIL4	24.54072	101.5544	4.14	ILMN_14855	PIWIL4	11
AKT1	2212.92	9166.52	4.14	ILMN_4841	AKT1	14
GOLGA2	135.9022	563.0469	4.14	ILMN_3191	GOLGA2	9
FERMT3	10.13583	42.00653	4.14	ILMN_1259	FERMT3	11
FANCA	66.07145	275.5956	4.17	ILMN_7097	FANCA	16
QRSL1	42.20701	176.0666	4.17	ILMN_5122	QRSL1	6

SH3BGR	32.1264	134.039	4.17	ILMN_28466	SH3BGR	21
SMARCC2	276.7168	1155.147	4.17	ILMN_5709	SMARCC2	12
BGLAP	34.86772	145.8011	4.18	ILMN_17038	BGLAP	1
CD3EAP	24.27177	101.5833	4.19	ILMN_23659	CD3EAP	19
CDKN1C	295.8516	1240.565	4.19	ILMN_20689	CDKN1C	11
SNAG1	5.277165	22.17167	4.2	ILMN_24881	SNAG1	5
UTRN	393.4115	1653.374	4.2	ILMN_15375	UTRN	6
HJURP	411.9676	1734.544	4.21	ILMN_137041	HJURP	2
ZIC5	58.60678	247.0567	4.22	ILMN_4790	ZIC5	13
SRA1	63.26855	266.8274	4.22	ILMN_4546	SRA1	5
ATP8B4	19.41424	82.10794	4.23	ILMN_20882	ATP8B4	15
GULP1	374.9214	1586.519	4.23	ILMN_11724	GULP1	2
FBXO16	108.6737	460.0029	4.23	ILMN_1262	FBXO16	8
AURKC	23.8763	101.0744	4.23	ILMN_13261	AURKC	19
PRKAR1B	256.0766	1085.543	4.24	ILMN_13026	PRKAR1B	7
NOPE	729.093	3091.085	4.24	ILMN_183764	NOPE	15
DPYS	1.403282	5.954288	4.24	ILMN_15302	DPYS	8
SETD4	929.4765	3945.618	4.24	ILMN_28718	SETD4	21
LOC400506	60.2156	255.7376	4.25	ILMN_24954	LOC400506	16
DCST1	22.62972	96.23904	4.25	ILMN_1322	DCST1	1
HDHD1A	220.7069	938.7528	4.25	ILMN_6926	HDHD1A	X
TTC18	111.8444	477.6405	4.27	ILMN_2757	TTC18	10
MKL1	9.428506	40.29654	4.27	ILMN_25866	MKL1	22
ECSIT	167.8104	717.7559	4.28	ILMN_27322	ECSIT	19
WWC2	42.01764	179.7172	4.28	ILMN_16250	WWC2	4
CC2D1A	184.3829	788.6674	4.28	ILMN_25829	CC2D1A	19
DGCR6	153.4976	657.3553	4.28	ILMN_32684	DGCR6	22
TIGD2	1282.608	5500.612	4.29	ILMN_24142	TIGD2	4
ZNF320	81.3985	349.7116	4.3	ILMN_165906	ZNF320	19
SFTA2	25.05401	107.8326	4.3	ILMN_7811	SFTA2	6
WAS	90.21098	388.6509	4.31	ILMN_1668	WAS	X
UGT2A3	12.46478	53.76287	4.31	ILMN_14617	UGT2A3	4
MGAT5	50.77369	219.0831	4.31	ILMN_21616	MGAT5	2
TBX20	2.566505	11.07667	4.32	ILMN_170117	TBX20	7
CBS	227.5215	982.8281	4.32	ILMN_5029	CBS	21
CTBP2	19.85962	85.87974	4.32	ILMN_20837	CTBP2	10
RNF31	556.3632	2408.947	4.33	ILMN_16732	RNF31	14
C9orf128	3.830014	16.58698	4.33	ILMN_7037	C9ORF128	9
UCHL5IP	881.0693	3818.448	4.33	ILMN_27285	UCHL5IP	X
TRAPPC6A	28.75736	124.6851	4.34	ILMN_1174	TRAPPC6A	19
PLA2G2F	2.901274	12.57993	4.34	ILMN_9383	PLA2G2F	1
C1orf168	12.96126	56.35231	4.35	ILMN_15128	C1ORF168	1
LST1	9.659168	42.08754	4.36	ILMN_22195	LST1	6
CUL7	10.50113	45.84013	4.37	ILMN_16366	CUL7	6
HSPA1A	275.7164	1204	4.37	ILMN_6623	HSPA1A	6
FLJ20850	7.315145	31.94705	4.37	ILMN_22393	FLJ20850	19
RPS19BP1	1038.89	4541.371	4.37	ILMN_8107	RPS19BP1	22

HPCA	121.868	533.1765	4.38	ILMN_20439	HPCA	1
MKRN3	107.1829	469.5235	4.38	ILMN_26675	MKRN3	15
POU3F2	85.382	374.7345	4.39	ILMN_174216	POU3F2	6
C15orf38	627.4822	2754.121	4.39	ILMN_18724	C15ORF38	15
DUS3L	654.4636	2874.237	4.39	ILMN_3805	DUS3L	19
ABHD11	186.6634	821.315	4.4	ILMN_24421	ABHD11	7
DPH1	87.77486	386.6894	4.41	ILMN_21347	DPH1	17
C10orf27	172.2367	759.0073	4.41	ILMN_21841	C10ORF27	10
SUPV3L1	1212.72	5344.461	4.41	ILMN_23318	SUPV3L1	10
HIST1H2AK	52.87803	233.1102	4.41	ILMN_26266	HIST1H2AK	6
MAPK13	2026.198	8943.256	4.41	ILMN_534	MAPK13	6
MAST4	2.334117	10.30246	4.41	ILMN_13686	MAST4	5
DUSP16	96.8273	427.3994	4.41	ILMN_12340	DUSP16	12
CCL5	29.87552	131.8837	4.41	ILMN_22732	CCL5	17
GNAT1	27.33189	120.9292	4.42	ILMN_21210	GNAT1	3
GPR128	52.23506	231.1997	4.43	ILMN_6384	GPR128	3
CCDC124	133.6386	591.7093	4.43	ILMN_10186	CCDC124	19
RPS6KA1	140.3199	621.6608	4.43	ILMN_2604	RPS6KA1	1
CPSF1	785.4919	3492.953	4.45	ILMN_22094	CPSF1	8
DHCR7	11.02503	49.03395	4.45	ILMN_2138	DHCR7	11
A4GALT	106.9061	475.7281	4.45	ILMN_25424	A4GALT	22
RANBP17	4.112325	18.31895	4.45	ILMN_7208	RANBP17	5
PCSK7	282.1814	1258.448	4.46	ILMN_29531	PCSK7	11
RGMA	121.7058	543.3226	4.46	ILMN_26077	RGMA	15
PSPH	1567.366	7007.266	4.47	ILMN_14445	PSPH	7
C9orf3	904.1131	4044.534	4.47	ILMN_19302	C9ORF3	9
TLE2	1412.612	6322.257	4.48	ILMN_5695	TLE2	19
HIST1H3H	367.3268	1645.531	4.48	ILMN_452	HIST1H3H	6
CCM2	336.9851	1512.619	4.49	ILMN_4086	CCM2	7
ID4	110.7837	497.4482	4.49	ILMN_23627	ID4	6
SLC11A1	32.74228	147.1012	4.49	ILMN_10134	SLC11A1	2
RBM38	74.26327	333.9636	4.5	ILMN_20092	RBM38	20
C1orf142	83.99834	377.86	4.5	ILMN_25687	C1ORF142	1
FAM80B	8.960333	40.41143	4.51	ILMN_19439	FAM80B	12
SECISBP2	376.2164	1697.246	4.51	ILMN_137193	SECISBP2	9
FOXA3	433.5134	1955.831	4.51	ILMN_22171	FOXA3	19
ADAM8	64.52266	291.3915	4.52	ILMN_138980	ADAM8	10
TNFRSF11A	10.23139	46.31801	4.53	ILMN_16552	TNFRSF11A	18
IQCD	385.1296	1750.464	4.55	ILMN_13421	IQCD	12
HHLA3	40.63141	185.1271	4.56	ILMN_14649	HHLA3	1
MTRR	201.4348	917.809	4.56	ILMN_13753	MTRR	5
PTPLA	1648.226	7511.721	4.56	ILMN_137280	PTPLA	10
ID3	1204.969	5492.42	4.56	ILMN_6829	ID3	1
COG7	174.2083	794.3218	4.56	ILMN_30252	COG7	16
CHD5	127.8275	583.4476	4.56	ILMN_26787	CHD5	1
LOC730302	2.15783	9.858815	4.57	ILMN_26794	LOC730302	
DGKH	27.95735	127.878	4.57	ILMN_26565	DGKH	13

RASSF6	32.60402	149.3703	4.58	ILMN_15686	RASSF6	4
ATP4A	39.61608	181.5418	4.58	ILMN_16471	ATP4A	19
PCSK9	18.4035	84.40524	4.59	ILMN_15492	PCSK9	1
RNF146	38.4693	176.5424	4.59	ILMN_3774	RNF146	6
SKIV2L	672.6918	3088.044	4.59	ILMN_646	SKIV2L	6
SYK	36.16063	166.0112	4.59	ILMN_8126	SYK	9
DYNC1I1	64.11423	294.7059	4.6	ILMN_7312	DYNC1I1	7
C1orf66	917.6419	4219.082	4.6	ILMN_20031	C1ORF66	1
DULLARD	122.9604	565.5686	4.6	ILMN_28688	DULLARD	17
DAZ4	6.848538	31.51568	4.6	ILMN_27809	DAZ4	Y
ORC1L	690.9858	3189.273	4.62	ILMN_21836	ORC1L	1
C18orf55	845.0109	3900.875	4.62	ILMN_9697	C18ORF55	18
CNTROB	96.47803	445.825	4.62	ILMN_22489	CNTROB	17
SPINK5	65.55977	302.9603	4.62	ILMN_10983	SPINK5	5
PYCRL	75.28155	348.0355	4.62	ILMN_14239	PYCRL	8
EBP	221.5122	1025.94	4.63	ILMN_12837	EBP	X
GPR37L1	120.3916	557.9942	4.63	ILMN_27850	GPR37L1	1
GCNT3	382.4688	1773.833	4.64	ILMN_11495	GCNT3	15
ZNF750	5.540722	25.71608	4.64	ILMN_12614	ZNF750	17
PXDNL	0.281966	1.309073	4.64	ILMN_10072	PXDNL	8
ALMS1	284.1483	1319.572	4.64	ILMN_138430	ALMS1	2
OGG1	90.92092	422.5275	4.65	ILMN_15380	OGG1	3
MYBBP1A	28.46437	132.3998	4.65	ILMN_10879	MYBBP1A	
EMR1	0.42361	1.971725	4.65	ILMN_12984	EMR1	19
PIGQ	326.732	1521.708	4.66	ILMN_14952	PIGQ	16
B3GNT4	59.55302	277.6497	4.66	ILMN_24050	B3GNT4	12
LRRC14	871.6714	4065.249	4.66	ILMN_29237	LRRC14	8
MED25	595.3946	2783.456	4.67	ILMN_19531	MED25	19
IL3RA	7.4561	34.88044	4.68	ILMN_19423	IL3RA	Y
LILRB3	98.84904	463.2997	4.69	ILMN_14901	LILRB3	19
PCF11	8.463722	39.73962	4.7	ILMN_29970	PCF11	11
GPR68	18.09389	84.97789	4.7	ILMN_25992	GPR68	14
ENTPD2	253.286	1190.996	4.7	ILMN_11079	ENTPD2	9
GRK6	128.9239	606.7352	4.71	ILMN_6856	GRK6	5
STIP1	1426.851	6727.954	4.72	ILMN_28761	STIP1	11
DGAT1	488.1922	2302.984	4.72	ILMN_6607	DGAT1	8
LONRF3	35.93528	169.8503	4.73	ILMN_11517	LONRF3	X
GPER	763.3555	3615.954	4.74	ILMN_13759	GPER	7
MGC16291	13.02742	61.75825	4.74	ILMN_29695	MGC16291	
CABIN1	1274.331	6044.161	4.74	ILMN_11059	CABIN1	22
ACAP3	23.42135	111.3911	4.76	ILMN_3810	ACAP3	1
C11orf2	3358.278	15988.18	4.76	ILMN_10940	C11ORF2	11
TRIM13	44.43452	211.6508	4.76	ILMN_22199	TRIM13	13
SGSM3	97.25211	463.7224	4.77	ILMN_20131	SGSM3	22
FCRL1	0.078606	0.375076	4.77	ILMN_8487	FCRL1	1
C2CD2L	54.31599	259.4214	4.78	ILMN_6441	C2CD2L	11
dJ222E13.2	111.4437	532.4942	4.78	ILMN_21268	DJ222E13.2	22

AP2A2	78.95975	377.6213	4.78	ILMN_3557	AP2A2	11
CLIP2	70.74977	338.3995	4.78	ILMN_14847	CLIP2	7
ADAD2	1.45868	6.979259	4.78	ILMN_870	ADAD2	16
C6orf145	7.405323	35.44197	4.79	ILMN_8767	C6ORF145	
TMEM34	16.3698	78.38764	4.79	ILMN_15674	TMEM34	4
RASD2	355.3421	1702.044	4.79	ILMN_21994	RASD2	22
BTNL3	3.192815	15.29817	4.79	ILMN_6666	BTNL3	5
ZNF213	213.1704	1023.561	4.8	ILMN_19299	ZNF213	16
AKAP14	112.1924	539.0811	4.8	ILMN_9633	AKAP14	X
PHC2	56.73678	273.7911	4.83	ILMN_7686	PHC2	1
DDX60	1318.99	6366.001	4.83	ILMN_17673	DDX60	4
FKSG44	1587.939	7665.622	4.83	ILMN_3387	FKSG44	11
SORCS2	14.12211	68.24877	4.83	ILMN_21543	SORCS2	4
C11orf41	18.51246	89.64007	4.84	ILMN_27502	C11ORF41	11
MAGEB18	6.836482	33.10759	4.84	ILMN_1530	MAGEB18	X
TMEM121	49.69437	240.7152	4.84	ILMN_26726	TMEM121	14
PCDHB12	340.4315	1650.295	4.85	ILMN_7611	PCDHB12	5
SATL1	23.37263	113.435	4.85	ILMN_19103	SATL1	X
WIF1	11.57212	56.22316	4.86	ILMN_7585	WIF1	12
ENTPD4	17.96949	87.42138	4.86	ILMN_19012	ENTPD4	8
NUP214	51.90763	253.6345	4.89	ILMN_29992	NUP214	9
TTC23L	4.841602	23.66307	4.89	ILMN_29633	TTC23L	5
TMEM139	187.4239	917.1527	4.89	ILMN_7268	TMEM139	7
KLF11	601.842	2945.828	4.89	ILMN_138189	KLF11	
PAX9	27.11033	132.8246	4.9	ILMN_1638	PAX9	14
SULT1C4	1.790846	8.775572	4.9	ILMN_4449	SULT1C4	2
MAP1D	5.853755	28.71908	4.91	ILMN_19115	MAP1D	2
MAK	168.0031	825.2372	4.91	ILMN_166619	MAK	6
MIA3	23.43184	115.2038	4.92	ILMN_181799	MIA3	1
MGC13057	154.1818	758.12	4.92	ILMN_181198	MGC13057	2
CCDC134	29.85842	146.8269	4.92	ILMN_2687	CCDC134	22
FRZB	119.1736	586.0378	4.92	ILMN_29091	FRZB	2
CD180	1.328864	6.537371	4.92	ILMN_6958	CD180	5
CACNA1B	25.25489	124.2763	4.92	ILMN_8944	CACNA1B	9
PNCK	80.31796	395.2437	4.92	ILMN_1597	PNCK	X
PDE2A	3.359804	16.53487	4.92	ILMN_14910	PDE2A	11
PWWP2B	193.4756	954.6152	4.93	ILMN_17964	PWWP2B	10
NPAT	105.3054	520.6926	4.94	ILMN_2928	NPAT	11
RBPMS	236.3787	1169.132	4.95	ILMN_13386	RBPMS	8
EHMT2	82.39026	408.0592	4.95	ILMN_18266	EHMT2	6
APOBEC1	49.52946	245.604	4.96	ILMN_17473	APOBEC1	12
PAK4	4.911638	24.3753	4.96	ILMN_19029	PAK4	19
KCNK6	14.37057	71.3262	4.96	ILMN_179974	KCNK6	19
PTPRN2	5.464518	27.12836	4.96	ILMN_778	PTPRN2	7
DKFZP564J	159.4243	795.5183	4.99	ILMN_182780	DKFZP564J102	4
ZNF280A	44.58626	222.5853	4.99	ILMN_22120	ZNF280A	22
RND1	18.14537	90.60603	4.99	ILMN_18161	RND1	12

TGFBR3	266.7193	1332.549	5	ILMN_22620	TGFBR3	1
IMAA	392.8443	1963.498	5	ILMN_138184	IMAA	16
KCNK5	7.009654	35.06723	5	ILMN_137293	KCNK5	6
MRPL34	409.6626	2052.291	5.01	ILMN_5839	MRPL34	19
SCRN2	24.23921	121.5417	5.01	ILMN_2736	SCRN2	17
SGCD	0.450294	2.263595	5.03	ILMN_9959	SGCD	5
SATB1	163.1479	821.596	5.04	ILMN_6836	SATB1	3
BTD	22.62212	113.9894	5.04	ILMN_24816	BTD	3
SOX8	3134.802	15817.07	5.05	ILMN_30180	SOX8	16
CAB39L	105.6631	533.2474	5.05	ILMN_9966	CAB39L	13
SHBG	83.67873	422.6963	5.05	ILMN_18112	SHBG	17
CEP72	163.0131	824.6936	5.06	ILMN_10995	CEP72	5
KCNH2	13.36593	67.691	5.06	ILMN_21609	KCNH2	7
C1orf222	11.90174	60.45197	5.08	ILMN_25308	C1ORF222	1
HLA-H	19.42817	98.68163	5.08	ILMN_5683	HLA-H	6
TFR2	95.08946	483.3984	5.08	ILMN_11788	TFR2	7
FAM69B	7.972346	40.54701	5.09	ILMN_138278	FAM69B	
GRLF1	15.64869	79.61108	5.09	ILMN_139122	GRLF1	19
C16orf45	5.959593	30.33436	5.09	ILMN_20964	C16ORF45	16
PIK3R4	52.25339	265.9959	5.09	ILMN_21306	PIK3R4	3
DDX53	20.49252	104.4623	5.1	ILMN_9714	DDX53	X
GNAT2	50.87162	259.5233	5.1	ILMN_4193	GNAT2	1
CES3	12.13489	61.96389	5.11	ILMN_172552	CES3	16
KANK2	19.15147	97.95815	5.11	ILMN_3271	KANK2	19
MAGEF1	98.1802	502.856	5.12	ILMN_23481	MAGEF1	3
CORO6	134.7459	690.7946	5.13	ILMN_16377	CORO6	17
MPI	18.76474	96.74839	5.16	ILMN_19042	MPI	15
AXIN1	513.713	2649.792	5.16	ILMN_6274	AXIN1	16
ICA1	264.9326	1367.017	5.16	ILMN_29651	ICA1	7
GPR175	50.72502	261.7911	5.16	ILMN_21308	GPR175	3
TAF8	56.97298	294.074	5.16	ILMN_25567	TAF8	6
PADI2	0.892976	4.611505	5.16	ILMN_7631	PADI2	1
UBXN11	51.14095	265.6205	5.19	ILMN_10090	UBXN11	1
MEGF8	4.549509	23.63815	5.2	ILMN_16368	MEGF8	19
PZP	2.077666	10.79667	5.2	ILMN_16323	PZP	12
MRM1	33.74429	175.3734	5.2	ILMN_9680	MRM1	17
PIGN	445.5097	2316.748	5.2	ILMN_163610	PIGN	
ITGB3	5.2962	27.58146	5.21	ILMN_22418	ITGB3	17
CCNE1	402.5705	2099.44	5.22	ILMN_6049	CCNE1	19
C21orf129	182.3716	951.1135	5.22	ILMN_9711	C21ORF129	21
CAPN10	5.485569	28.64589	5.22	ILMN_15144	CAPN10	2
ARX	14.09592	73.70753	5.23	ILMN_4672	ARX	X
SNAP25	512.8938	2683.794	5.23	ILMN_7893	SNAP25	20
CDCA1	6.08755	31.96112	5.25	ILMN_17725	CDCA1	1
GDPD2	7.896815	41.47701	5.25	ILMN_12706	GDPD2	X
RASA2	32.76795	172.6509	5.27	ILMN_15489	RASA2	3
ACVR1C	14.87177	78.51275	5.28	ILMN_11457	ACVR1C	2

C12orf72	12.13839	64.11423	5.28	ILMN_25281	C12ORF72	12
ZNF530	7.951055	42.09807	5.29	ILMN_16961	ZNF530	19
C10orf125	402.1339	2133.511	5.31	ILMN_14268	C10ORF125	10
SLC30A6	414.2202	2199.122	5.31	ILMN_11820	SLC30A6	2
PGAP1	282.9025	1502.695	5.31	ILMN_173416	PGAP1	2
CLDN1	2191.616	11654.28	5.32	ILMN_24855	CLDN1	3
PRKCB1	17.47531	93.09595	5.33	ILMN_12900	PRKCB1	16
GARNL3	106.0528	566.027	5.34	ILMN_16048	GARNL3	9
KRBA2	26.76538	143.0047	5.34	ILMN_21718	KRBA2	17
DNPEP	115.549	618.9415	5.36	ILMN_13146	DNPEP	2
POTE2	34.49108	184.8872	5.36	ILMN_20336	POTE2	2
YBX2	397.4352	2135.002	5.37	ILMN_3458	YBX2	
RAB20	52.07677	279.901	5.37	ILMN_13192	RAB20	13
POLN	3.669623	19.78757	5.39	ILMN_9365	POLN	4
NOD1	420.9178	2270.835	5.39	ILMN_10741	NOD1	7
PTPRO	22.89883	123.5902	5.4	ILMN_7758	PTPRO	12
PRSS3	472.704	2551.854	5.4	ILMN_19426	PRSS3	9
ZNF566	154.2768	836.369	5.42	ILMN_16016	ZNF566	19
MTMR15	122.7611	666.0233	5.43	ILMN_26764	MTMR15	15
KIAA0644	5.090982	27.66755	5.43	ILMN_6143	KIAA0644	7
JMJD2A	5.594975	30.4128	5.44	ILMN_11094	JMJD2A	1
CITED4	436.5604	2374.768	5.44	ILMN_15271	CITED4	1
DOT1L	15.04664	81.89239	5.44	ILMN_28624	DOT1L	19
CNNM4	63.42505	345.6643	5.45	ILMN_16517	CNNM4	2
IL1F7	8.734919	47.61145	5.45	ILMN_13150	IL1F7	2
C21orf119	403.0024	2196.733	5.45	ILMN_748	C21ORF119	21
CAMK2B	57.0816	311.6379	5.46	ILMN_19817	CAMK2B	7
PRKCABP	223.7632	1223.673	5.47	ILMN_138706	PRKCABP	22
HIST2H2BE	7.981238	43.69485	5.47	ILMN_28293	HIST2H2BE	1
NPNT	12.44847	68.2942	5.49	ILMN_20015	NPNT	4
CKB	857.1395	4704.037	5.49	ILMN_10755	CKB	14
LPCAT4	68.52024	376.2164	5.49	ILMN_137968	LPCAT4	15
MSR1	1.647881	9.048601	5.49	ILMN_18744	MSR1	8
B3GNT8	15.19887	83.56354	5.5	ILMN_3909	B3GNT8	19
COX7B2	136.4769	752.4069	5.51	ILMN_19288	COX7B2	4
MYH7	7.428099	41.05693	5.53	ILMN_4628	MYH7	14
KIAA1303	15.12271	83.62344	5.53	ILMN_4192	KIAA1303	17
ATP6V0D1	359.4533	1989.571	5.53	ILMN_139373	ATP6V0D1	16
IGFL4	16.7358	92.79663	5.54	ILMN_7748	IGFL4	19
KCNQ2	383.0941	2125.356	5.55	ILMN_20672	KCNQ2	20
AKAP3	9.040092	50.16034	5.55	ILMN_11088	AKAP3	12
RIPK1	181.6918	1008.596	5.55	ILMN_24351	RIPK1	6
DSCR4	1.683259	9.348625	5.55	ILMN_3382	DSCR4	21
OCLM	8.588411	47.7225	5.56	ILMN_26239	OCLM	1
TMEM80	63.326	352.1914	5.56	ILMN_138465	TMEM80	11
STAT5B	379.4526	2111.826	5.57	ILMN_16004	STAT5B	17
S100A1	65.83412	366.6512	5.57	ILMN_7545	S100A1	1

SLC28A1	0.809148	4.506633	5.57	ILMN_8249	SLC28A1	15
IRAK3	7.25413	40.40321	5.57	ILMN_25857	IRAK3	12
CYP2S1	815.4988	4544.5	5.57	ILMN_137773	CYP2S1	19
SH3GLB2	3184.038	17747.71	5.57	ILMN_14480	SH3GLB2	9
TAT	13.98006	78.02293	5.58	ILMN_6875	TAT	16
NEK5	99.05434	553.2068	5.58	ILMN_2111	NEK5	13
CD1D	17.15455	95.81525	5.59	ILMN_926	CD1D	1
C9orf152	370.0291	2067.762	5.59	ILMN_753	C9ORF152	9
OTX2	1.040714	5.835052	5.61	ILMN_16095	OTX2	14
AMDHD2	73.21616	411.1364	5.62	ILMN_26534	AMDHD2	16
RNASE1	222.0376	1247.913	5.62	ILMN_22847	RNASE1	14
TLN2	696.0605	3919.034	5.63	ILMN_17132	TLN2	15
SMEK3P	8.384879	47.21068	5.63	ILMN_12552	SMEK3P	X
FAM100A	68.14708	383.8956	5.63	ILMN_3918	FAM100A	16
FAM125B	146.8433	829.6611	5.65	ILMN_20760	FAM125B	9
ACP6	551.371	3122.8	5.66	ILMN_24835	ACP6	1
ALPL	717.2797	4063.658	5.67	ILMN_4097	ALPL	1
CHERP	243.4413	1380.3	5.67	ILMN_19450	CHERP	19
CYP26A1	59.02234	335.468	5.68	ILMN_27300	CYP26A1	10
SARDH	46.6878	265.787	5.69	ILMN_8646	SARDH	9
KIAA0802	81.06773	462.1727	5.7	ILMN_22877	KIAA0802	18
IL17RE	18.76427	107.1695	5.71	ILMN_3347	IL17RE	3
PLCB4	3.003418	17.16003	5.71	ILMN_177768	PLCB4	20
GPR120	55.99206	320.3015	5.72	ILMN_17551	GPR120	10
PRSS12	42.75994	244.7005	5.72	ILMN_19375	PRSS12	4
PPP1R14D	75.90652	434.6464	5.73	ILMN_24809	PPP1R14D	15
C9orf98	22.36625	128.263	5.73	ILMN_18587	C9ORF98	9
PRNPIP	2.40833	13.83732	5.75	ILMN_5651	PRNPIP	1
ABLIM2	465.0415	2683.408	5.77	ILMN_5259	ABLIM2	4
PPP2R3B	63.31076	365.6612	5.78	ILMN_5735	PPP2R3B	Y
WNT10A	170.8721	987.5199	5.78	ILMN_12046	WNT10A	2
HLA-A29.1	1131.188	6550.952	5.79	ILMN_168174	HLA-A29.1	
LRRC61	52.85949	306.1615	5.79	ILMN_6674	LRRC61	7
ELOVL4	5.588629	32.37316	5.79	ILMN_9781	ELOVL4	6
ZNF587	35.5102	206.1339	5.8	ILMN_15675	ZNF587	19
ONECUT1	2.876789	16.70033	5.81	ILMN_1269	ONECUT1	15
MPDU1	53.81427	312.8299	5.81	ILMN_28782	MPDU1	17
SNTB1	936.6044	5448.279	5.82	ILMN_23979	SNTB1	8
C6orf168	72.60048	422.5804	5.82	ILMN_24015	C6ORF168	6
PDZD4	14.60536	85.26364	5.84	ILMN_3986	PDZD4	X
ZNF573	55.19764	322.9445	5.85	ILMN_23003	ZNF573	19
CHMP6	147.2044	861.3077	5.85	ILMN_26654	CHMP6	
SDCCAG8	59.43603	348.4397	5.86	ILMN_24496	SDCCAG8	1
BAIAP2L2	677.8925	3978.097	5.87	ILMN_21982	BAIAP2L2	22
MAGEC1	18.99571	111.9134	5.89	ILMN_173346	MAGEC1	X
GPR114	775.7434	4571.689	5.89	ILMN_1415	GPR114	16
WDR45L	293.923	1733.229	5.9	ILMN_10751	WDR45L	17

PCDHA13	1.871226	11.04508	5.9	ILMN_29927	PCDHA13	5
ZADH2	304.1905	1796.643	5.91	ILMN_5633	ZADH2	18
BANP	14.61294	86.32146	5.91	ILMN_9555	BANP	16
ORM1	441.5833	2608.623	5.91	ILMN_17138	ORM1	9
MADD	225.9587	1335.502	5.91	ILMN_9428	MADD	11
PRIM2	187.7003	1112.9	5.93	ILMN_27188	PRIM2	6
RASL11A	591.1727	3517.562	5.95	ILMN_17784	RASL11A	13
ZNF439	16.28545	97.29121	5.97	ILMN_2860	ZNF439	19
KCNK2	3.379501	20.22722	5.99	ILMN_4676	KCNK2	1
STX19	513.9211	3079.26	5.99	ILMN_17396	STX19	3
GMPR	32.0969	192.5041	6	ILMN_2416	GMPR	6
UGT2B10	14.70881	88.42818	6.01	ILMN_4885	UGT2B10	4
TMEM9	912.3795	5488.351	6.02	ILMN_3989	TMEM9	1
KRT26	1.031597	6.205532	6.02	ILMN_22354	KRT26	17
ALOX15	6.005861	36.14192	6.02	ILMN_182092	ALOX15	17
RGS11	7.889206	47.53417	6.03	ILMN_8284	RGS11	16
FGFR4	375.903	2266.67	6.03	ILMN_29492	FGFR4	5
WDR24	216.0784	1303.463	6.03	ILMN_26740	WDR24	16
CCDC12	87.65271	531.0127	6.06	ILMN_20382	CCDC12	3
HNT	1.305367	7.911388	6.06	ILMN_169728	HNT	11
SEC11A	30.4128	184.3829	6.06	ILMN_12976	SEC11A	15
DAZAP1	11.74285	71.20806	6.06	ILMN_8299	DAZAP1	19
FAM101B	223.4237	1357.507	6.08	ILMN_1388	FAM101B	17
ITGB2	112.5165	683.7878	6.08	ILMN_42184	ITGB2	21
CALCR	0.849024	5.185917	6.11	ILMN_5197	CALCR	7
HOXA7	15.22876	93.03408	6.11	ILMN_138212	HOXA7	7
RASSF10	5.283126	32.28822	6.11	ILMN_183301	RASSF10	11
TOP3A	381.7553	2338.324	6.13	ILMN_167915	TOP3A	17
HCCA2	39.43869	242.9682	6.16	ILMN_29092	HCCA2	11
UCK1	148.3091	914.1101	6.16	ILMN_11227	UCK1	9
PAX2	105.7991	652.2426	6.16	ILMN_2482	PAX2	10
DDX49	2.810897	17.35447	6.17	ILMN_13684	DDX49	19
DSG4	74.79826	461.9163	6.18	ILMN_17707	DSG4	18
TAS2R5	23.57205	145.9372	6.19	ILMN_23037	TAS2R5	7
BCL2L14	16.86666	104.4495	6.19	ILMN_14286	BCL2L14	12
FBXL8	73.7272	457.0065	6.2	ILMN_24638	FBXL8	16
C19orf36	4.250726	26.35724	6.2	ILMN_137890	C19ORF36	19
EPHX1	21.28022	132.1513	6.21	ILMN_5212	EPHX1	1
HEPACAM2	2.079599	12.92984	6.22	ILMN_1501	HEPACAM2	7
SLC22A10	0.792225	4.927817	6.22	ILMN_44306	SLC22A10	
C16orf85	0.789956	4.933402	6.25	ILMN_29616	C16ORF85	16
KIF17	76.34241	476.8914	6.25	ILMN_7755	KIF17	1
KCNMB3	6.533367	40.89283	6.26	ILMN_26748	KCNMB3	3
PRIMA1	10.40597	65.15032	6.26	ILMN_7346	PRIMA1	14
C8G	53.50163	335.1874	6.26	ILMN_1016	C8G	9
JRK	174.5659	1094.944	6.27	ILMN_11460	JRK	8
IGSF5	12.57091	78.8821	6.27	ILMN_171639	IGSF5	21

VCP	11.27672	71.3622	6.33	ILMN_25548	VCP	9
SERPINA3	5.36752	33.97297	6.33	ILMN_16107	SERPINA3	14
SYAP1	245.7203	1555.704	6.33	ILMN_8037	SYAP1	X
FUNDC2	22.44485	142.1382	6.33	ILMN_615	FUNDC2	X
HOXB6	14.91514	94.63827	6.35	ILMN_138898	HOXB6	17
SYCP2	11.59742	73.61253	6.35	ILMN_173326	SYCP2	20
DNAH3	4.902044	31.18357	6.36	ILMN_27452	DNAH3	16
ZNF394	68.5606	438.2646	6.39	ILMN_25541	ZNF394	7
DLX3	12.98332	83.67873	6.45	ILMN_4634	DLX3	17
TRIO	39.26464	253.2493	6.45	ILMN_23876	TRIO	5
FAAH	49.7413	321.4483	6.46	ILMN_12578	FAAH	1
APOC2	10.08721	65.3464	6.48	ILMN_26723	APOC2	19
COL22A1	19.19052	124.47	6.49	ILMN_18096	COL22A1	8
C16orf13	212.5759	1379.032	6.49	ILMN_20858	C16ORF13	16
MSH3	370.6981	2407.598	6.49	ILMN_176593	MSH3	5
CCDC114	95.43765	622.1132	6.52	ILMN_16523	CCDC114	19
TMEM104	6.554173	42.81808	6.53	ILMN_14713	TMEM104	17
TESC	68.19286	446.1359	6.54	ILMN_25089	TESC	12
RAB11B	327.5537	2149.209	6.56	ILMN_3799	RAB11B	19
CENPH	1115.706	7321.56	6.56	ILMN_163931	CENPH	5
FGFR1	49.68299	328.0042	6.6	ILMN_7476	FGFR1	8
SDC1	5.632334	37.20969	6.61	ILMN_2541	SDC1	2
C15orf37	6.578671	43.50734	6.61	ILMN_18611	C15ORF37	15
SLC1A4	21.04977	139.4971	6.63	ILMN_12585	SLC1A4	2
VSIG2	4.429685	29.35833	6.63	ILMN_23332	VSIG2	11
SCN9A	1.466189	9.717733	6.63	ILMN_173139	SCN9A	2
C17orf47	1.309607	8.686154	6.63	ILMN_24191	C17ORF47	17
CLCN4	88.03242	585.253	6.65	ILMN_29785	CLCN4	X
C16orf73	482.8584	3219.785	6.67	ILMN_16054	C16ORF73	16
LOC389151	5.119438	34.23042	6.69	ILMN_17549	LOC389151	3
RNASEH2C	4.937475	33.19935	6.72	ILMN_29121	RNASEH2C	11
ZNF544	1.48884	10.06833	6.76	ILMN_183212	ZNF544	19
RDH5	382.3182	2590.715	6.78	ILMN_20119	RDH5	12
DDX58	34.20064	232.3997	6.8	ILMN_139289	DDX58	9
GTF3C5	617.3391	4198.643	6.8	ILMN_814	GTF3C5	9
MPG	12.54935	85.41419	6.81	ILMN_4875	MPG	16
MPDZ	12.29829	83.71458	6.81	ILMN_8353	MPDZ	9
TUBB8	2.785022	18.99408	6.82	ILMN_27641	TUBB8	10
VCY	3.777369	25.76829	6.82	ILMN_14469	VCY	Y
IL20RA	123.885	846.0981	6.83	ILMN_16908	IL20RA	6
PTPN18	20.07518	137.1079	6.83	ILMN_14552	PTPN18	2
CRAMP1L	264.499	1806.61	6.83	ILMN_12672	CRAMP1L	16
PPAN-P2RY	0.411852	2.814068	6.83	ILMN_169217	PPAN-P2RY11	19
LMOD1	8.515432	58.18502	6.83	ILMN_29626	LMOD1	1
OS9	5.572892	38.08783	6.83	ILMN_26656	OS9	12
CENPM	424.4785	2908.254	6.85	ILMN_12351	CENPM	22
WSCD1	12.90264	88.64977	6.87	ILMN_23483	WSCD1	17

PTP4A3	36.6426	252.4029	6.89	ILMN_14079	PTP4A3	8
FLJ45139	9.045718	62.51271	6.91	ILMN_4616	FLJ45139	21
PPARGC1B	11.29199	78.12951	6.92	ILMN_5828	PPARGC1B	5
SUPT6H	102.251	707.595	6.92	ILMN_7343	SUPT6H	17
GOLT1A	387.3212	2684.956	6.93	ILMN_23783	GOLT1A	1
TIA1	8.001587	55.62338	6.95	ILMN_29910	TIA1	2
HAP1	28.68262	199.509	6.96	ILMN_16907	HAP1	17
SIGLEC15	4.13673	28.92355	6.99	ILMN_26132	SIGLEC15	18
C15orf42	178.2088	1246.144	6.99	ILMN_26336	C15ORF42	15
TCEB3	761.8686	5334.042	7	ILMN_6761	TCEB3	1
CCDC57	27.58146	193.4756	7.01	ILMN_26012	CCDC57	17
TNKS	47.24415	331.6778	7.02	ILMN_138872	TNKS	8
METT10D	51.99841	365.4027	7.03	ILMN_9914	METT10D	17
HIST3H2A	79.42422	559.7332	7.05	ILMN_16029	HIST3H2A	1
ADAM17	540.823	3815.002	7.05	ILMN_165100	ADAM17	2
HEPH	273.06	1928.401	7.06	ILMN_7949	HEPH	X
ROCK2	853.7422	6031.012	7.06	ILMN_23757	ROCK2	2
TP53	336.4649	2378.349	7.07	ILMN_11285	TP53	17
TYRO3P	8.32581	58.99857	7.09	ILMN_4506	TYRO3P	
GATA3	30.26138	214.6211	7.09	ILMN_6387	GATA3	10
ANXA13	12.20994	86.75302	7.11	ILMN_19368	ANXA13	8
TGM3	11.35621	80.8798	7.12	ILMN_10351	TGM3	20
NFE2	775.3411	5551.534	7.16	ILMN_5749	NFE2	12
ZC3H12A	24.79227	177.5454	7.16	ILMN_18682	ZC3H12A	1
DALRD3	5.548689	39.89106	7.19	ILMN_12427	DALRD3	3
ALOXE3	6.609578	47.52215	7.19	ILMN_29198	ALOXE3	17
VAX2	15.40218	110.874	7.2	ILMN_20833	VAX2	2
MGC26718	47.47142	342.3373	7.21	ILMN_27823	MGC26718	18
NPM2	43.29572	312.2382	7.21	ILMN_28626	NPM2	8
ONECUT3	16.17429	116.7707	7.22	ILMN_180935	ONECUT3	19
TMPRSS5	47.61077	343.7405	7.22	ILMN_26590	TMPRSS5	11
WASF3	244.4406	1765.424	7.22	ILMN_9535	WASF3	13
NEURL2	7.180059	51.86234	7.22	ILMN_20666	NEURL2	20
UBE2J2	53.7084	388.0699	7.23	ILMN_13593	UBE2J2	1
PI3	29.30158	212.1266	7.24	ILMN_13685	PI3	20
ANXA9	19.49067	141.2393	7.25	ILMN_22287	ANXA9	1
RPP25	180.7098	1309.623	7.25	ILMN_26499	RPP25	15
SLC35E4	40.52102	293.8138	7.25	ILMN_26184	SLC35E4	22
LDLRAD3	53.61244	389.2534	7.26	ILMN_26811	LDLRAD3	11
CNIH3	24.11431	175.6542	7.28	ILMN_1447	CNIH3	1
LRP4	334.3104	2440.387	7.3	ILMN_18077	LRP4	11
GPC3	986.6259	7215.583	7.31	ILMN_24093	GPC3	X
AMIGO3	7.682772	56.19411	7.31	ILMN_25473	AMIGO3	3
ZNF626	1.86592	13.72444	7.36	ILMN_137796	ZNF626	19
HIST1H2AM	128.6073	949.3016	7.38	ILMN_26622	HIST1H2AM	6
ZNF397	78.33949	578.6213	7.39	ILMN_6841	ZNF397	18
PLEKHJ1	7.610446	56.24582	7.39	ILMN_936	PLEKHJ1	19

PHOSPHO1	2.947323	21.79542	7.39	ILMN_4436	PHOSPHO1	17
RPS26	1227.631	9113.008	7.42	ILMN_1912	RPS26	12
NFIB	108.4631	805.7385	7.43	ILMN_14904	NFIB	9
SLC12A8	10.78175	80.14137	7.43	ILMN_11758	SLC12A8	3
DNALI1	585.9063	4356.863	7.44	ILMN_24038	DNALI1	1
CDYL2	19.13923	142.458	7.44	ILMN_19054	CDYL2	16
TBRG4	186.65	1389.53	7.44	ILMN_24003	TBRG4	7
ARSA	0.693113	5.180296	7.47	ILMN_17279	ARSA	22
ONECUT2	13.98639	104.6	7.48	ILMN_27185	ONECUT2	18
CEACAM3	0.540116	4.040619	7.48	ILMN_21499	CEACAM3	19
RNF5	15.98349	119.6895	7.49	ILMN_1529	RNF5	6
WHSC1	12.06687	90.51537	7.5	ILMN_27418	WHSC1	4
PLAC1	32.79068	246.5664	7.52	ILMN_24438	PLAC1	X
CCDC101	100.3964	755.5676	7.53	ILMN_5379	CCDC101	16
C7orf28A	21.09854	158.9043	7.53	ILMN_2087	C7ORF28A	7
FOXF1	5.273596	39.76083	7.54	ILMN_11804	FOXF1	16
UHRF1	92.66936	699.1837	7.54	ILMN_12005	UHRF1	19
P2RY1	136.8908	1037.499	7.58	ILMN_19264	P2RY1	3
AK7	3.915335	29.7211	7.59	ILMN_29133	AK7	14
EPHB2	224.48	1704.248	7.59	ILMN_6179	EPHB2	1
CDKL1	18.01805	137.2057	7.61	ILMN_7869	CDKL1	14
EMX2	4.375477	33.37553	7.63	ILMN_181483	EMX2	10
EFNA4	96.62995	737.6046	7.63	ILMN_18185	EFNA4	1
ASB10	7.863967	60.28288	7.67	ILMN_20447	ASB10	7
USP2	5.168635	39.66745	7.67	ILMN_7894	USP2	11
SH2B1	82.94837	636.6725	7.68	ILMN_26582	SH2B1	16
TFE3	1.371922	10.5333	7.68	ILMN_16136	TFE3	X
SGSH	22.45551	172.4953	7.68	ILMN_7542	SGSH	17
DDAH2	69.74818	535.8364	7.68	ILMN_2018	DDAH2	6
OR9A2	1.015843	7.814619	7.69	ILMN_22019	OR9A2	7
FUT10	114.3191	880.6465	7.7	ILMN_18464	FUT10	8
C20orf191	32.61717	252.5246	7.74	ILMN_25650	C20ORF191	20
FRAT1	9.697061	75.07609	7.74	ILMN_29787	FRAT1	10
ABCC8	5.123323	39.70177	7.75	ILMN_4012	ABCC8	11
GATA6	16.2392	126.0733	7.76	ILMN_15987	GATA6	18
SPTBN2	41.97982	326.0647	7.77	ILMN_5653	SPTBN2	11
RYR1	101.5854	791.7086	7.79	ILMN_163470	RYR1	19
C16orf54	0.160763	1.253004	7.79	ILMN_16593	C16ORF54	16
AIP	973.2538	7592.219	7.8	ILMN_13720	AIP	11
MRTO4	677.2807	5283.582	7.8	ILMN_1930	MRTO4	1
MAGEB6	4.343811	33.91005	7.81	ILMN_11130	MAGEB6	X
GTF3C6	1.34352	10.49523	7.81	ILMN_16996	GTF3C6	6
HSPC268	3.259404	25.46481	7.81	ILMN_485	HSPC268	7
ZNF205	151.8972	1189.366	7.83	ILMN_4939	ZNF205	16
RASSF3	25.90439	203.0747	7.84	ILMN_20097	RASSF3	12
TTTY14	3.80038	29.80566	7.84	ILMN_8832	TTTY14	Y
LRRC19	16.23462	127.4199	7.85	ILMN_21980	LRRC19	9

FCHSD1	4.185102	32.85933	7.85	ILMN_3885	FCHSD1	5
SAMD9	365.1956	2867.625	7.85	ILMN_17677	SAMD9	7
GAL3ST2	21.95076	173.0194	7.88	ILMN_2364	GAL3ST2	2
TNNC1	205.0005	1617.21	7.89	ILMN_3636	TNNC1	3
IGFBP4	707.2228	5579.688	7.89	ILMN_9309	IGFBP4	17
UNC93B1	869.3166	6860.807	7.89	ILMN_179886	UNC93B1	11
TBC1D22A	14.02651	111.147	7.92	ILMN_29869	TBC1D22A	22
FLJ35220	84.97789	673.6957	7.93	ILMN_21251	FLJ35220	17
MBD1	46.64977	370.2471	7.94	ILMN_14073	MBD1	18
OSCAR	33.0453	262.4125	7.94	ILMN_3784	OSCAR	19
KLK11	105.5301	840.6431	7.97	ILMN_3680	KLK11	19
SIRT6	170.3873	1358.145	7.97	ILMN_1560	SIRT6	19
UCP2	353.0772	2817.192	7.98	ILMN_14172	UCP2	11
CAMKK1	9.250757	73.86464	7.98	ILMN_29266	CAMKK1	17
AKR7A3	348.4397	2813.592	8.07	ILMN_173325	AKR7A3	1
IFRD2	461.508	3735.68	8.09	ILMN_17691	IFRD2	3
HAPLN3	160.2644	1297.562	8.1	ILMN_19816	HAPLN3	15
CUX1	35.47744	287.2587	8.1	ILMN_8630	CUX1	7
GNMT	22.30078	180.7098	8.1	ILMN_9426	GNMT	6
SORL1	18.70422	151.5727	8.1	ILMN_162864	SORL1	11
THBS2	6.307381	51.15998	8.11	ILMN_16098	THBS2	6
MOCS2	14.00835	113.6619	8.11	ILMN_27055	MOCS2	5
MUC17	86.27348	700.3734	8.12	ILMN_3632	MUC17	7
HRCT1	249.4019	2025.682	8.12	ILMN_30559	HRCT1	9
LGALS2	1.919179	15.60553	8.13	ILMN_19736	LGALS2	22
EHMT1	20.94953	170.5808	8.14	ILMN_18594	EHMT1	9
ZDHHC24	12.27772	100.1031	8.15	ILMN_10196	ZDHHC24	11
LOC649431	15.99652	130.8309	8.18	ILMN_34578	LOC649431	
LGALS9	2.788307	22.81567	8.18	ILMN_667	LGALS9	17
C9orf80	94.37489	773.6169	8.2	ILMN_27473	C9ORF80	9
CA12	729.9719	5987.72	8.2	ILMN_162051	CA12	15
WHSC2	7.436594	61.01812	8.21	ILMN_4205	WHSC2	4
MBD4	146.1004	1206.455	8.26	ILMN_18891	MBD4	3
RGS14	14.22651	117.6446	8.27	ILMN_24426	RGS14	5
CDKN2A	114.2856	945.5816	8.27	ILMN_30355	CDKN2A	9
GUCA2A	29.05707	240.5589	8.28	ILMN_28166	GUCA2A	1
TMEM105	2.742197	22.76073	8.3	ILMN_12849	TMEM105	17
BTNL9	104.976	872.8219	8.31	ILMN_18011	BTNL9	5
CCDC9	44.68513	371.8739	8.32	ILMN_5456	CCDC9	19
LBX2	33.26169	277.0581	8.33	ILMN_14162	LBX2	2
OVOL2	560.1711	4670.668	8.34	ILMN_17074	OVOL2	20
PKP3	7.069797	59.01303	8.35	ILMN_27019	PKP3	11
ZNF701	12.09412	100.9853	8.35	ILMN_15897	ZNF701	19
TUBGCP2	385.8723	3231.347	8.37	ILMN_138802	TUBGCP2	10
TCEA3	215.2286	1802.961	8.38	ILMN_27218	TCEA3	1
RTDR1	66.22933	554.8978	8.38	ILMN_4141	RTDR1	22
CMPK2	16.061	134.6803	8.39	ILMN_3648	CMPK2	2

STMN4	1.440476	12.11111	8.41	ILMN_24504	STMN4	8
LLGL2	71.81721	606.097	8.44	ILMN_17586	LLGL2	17
GEMIN5	210.1334	1776.612	8.45	ILMN_28383	GEMIN5	5
ARHGAP4	18.08779	152.9847	8.46	ILMN_2811	ARHGAP4	X
VASH2	36.41505	308.0689	8.46	ILMN_3016	VASH2	1
CDH24	11.42892	96.85644	8.47	ILMN_791	CDH24	14
FBXL15	296.5327	2517.912	8.49	ILMN_4176	FBXL15	10
LUM	10.05989	85.73994	8.52	ILMN_4223	LUM	12
LOC65375	5.927487	50.62252	8.54	ILMN_43990	LOC653759	2
TRPC3	47.53651	406.5685	8.55	ILMN_1898	TRPC3	4
ATP8B1	132.8246	1136.051	8.55	ILMN_27796	ATP8B1	18
KCNN1	1.759022	15.04664	8.55	ILMN_18517	KCNN1	19
RCC2	1465.846	12556.8	8.57	ILMN_2123	RCC2	1
KIAA1324	124.8143	1071.263	8.58	ILMN_9289	KIAA1324	1
DAPK2	91.22498	784.3278	8.6	ILMN_2606	DAPK2	15
HYOU1	65.01265	559.1628	8.6	ILMN_167916	HYOU1	11
TXNDC12	1789.086	15424.22	8.62	ILMN_22592	TXNDC12	1
KCNJ3	50.23192	433.1866	8.62	ILMN_10841	KCNJ3	2
PNPLA2	103.1453	890.3889	8.63	ILMN_27831	PNPLA2	11
HCK	37.42601	323.1306	8.63	ILMN_16321	HCK	20
TMEM63C	45.02567	389.7856	8.66	ILMN_13193	TMEM63C	14
OSAP	7.736088	67.02222	8.66	ILMN_7551	OSAP	4
SCLT1	21.50408	186.4795	8.67	ILMN_19208	SCLT1	4
CYP2C18	11.19519	97.1628	8.68	ILMN_4940	CYP2C18	10
EPPB9	55.41657	481.0978	8.68	ILMN_9318	EPPB9	17
EMID1	34.58247	300.6412	8.69	ILMN_18992	EMID1	22
KLF9	160.4428	1397.122	8.71	ILMN_2670	KLF9	9
SNCAIP	525.7431	4584.042	8.72	ILMN_9515	SNCAIP	5
ARFGAP2	27.82706	243.21	8.74	ILMN_12944	ARFGAP2	11
C15orf39	15.21651	133.4785	8.77	ILMN_18382	C15ORF39	15
PRR17	0.317606	2.788187	8.78	ILMN_10135	PRR17	20
MAP3K15	66.65401	586.4588	8.8	ILMN_22910	MAP3K15	X
C21orf24	1.533532	13.50746	8.81	ILMN_1369	C21ORF24	21
SLC22A16	58.41114	514.8676	8.81	ILMN_26256	SLC22A16	6
ZNF615	291.4635	2573.392	8.83	ILMN_26774	ZNF615	19
CDH11	188.477	1669.686	8.86	ILMN_11789	CDH11	16
DSCR6	46.8665	416.0488	8.88	ILMN_18308	DSCR6	21
C17orf55	37.82184	335.8026	8.88	ILMN_17830	C17ORF55	17
EDN2	12.45736	111.2153	8.93	ILMN_21872	EDN2	1
LOC44224	1.069052	9.55565	8.94	ILMN_33094	LOC442245	6
TBCD	305.7176	2732.803	8.94	ILMN_23465	TBCD	17
IPPK	25.91562	232.0032	8.95	ILMN_29452	IPPK	9
MED19	143.8794	1288.161	8.95	ILMN_24030	MED19	11
TRIM38	66.07188	592.6497	8.97	ILMN_13190	TRIM38	6
CPSF4	602.5042	5406.554	8.97	ILMN_178236	CPSF4	7
ABHD12B	209.6185	1882.046	8.98	ILMN_3950	ABHD12B	14
SLC38A5	33.58558	301.6877	8.98	ILMN_26284	SLC38A5	X

BRD4	96.08138	868.1434	9.04	ILMN_22169	BRD4	19
FLJ44112	7.803576	70.95259	9.09	ILMN_27749	FLJ44112	12
PTGDS	12.77188	116.1607	9.1	ILMN_19248	PTGDS	9
KCNK10	13.66422	124.4108	9.1	ILMN_22948	KCNK10	14
CACNB2	20.43315	186.2181	9.11	ILMN_2192	CACNB2	10
CTCF	201.2383	1839.443	9.14	ILMN_22198	CTCF	16
AKNA	84.25967	770.8203	9.15	ILMN_5357	AKNA	
PDZK1IP1	34.50398	315.8477	9.15	ILMN_968	PDZK1IP1	1
MTA2	94.97527	870.5527	9.17	ILMN_11985	MTA2	11
TNFAIP8L3	30.69669	282.7829	9.21	ILMN_9624	TNFAIP8L3	15
NPTX1	79.85725	735.7716	9.21	ILMN_5048	NPTX1	17
KIAA0133	183.0045	1687.334	9.22	ILMN_5835	KIAA0133	1
DOK4	34.51673	318.5249	9.23	ILMN_5422	DOK4	16
ATG16L2	30.45865	281.4026	9.24	ILMN_16649	ATG16L2	11
C6orf170	38.04233	351.6074	9.24	ILMN_17001	C6ORF170	6
PTHR1	5.254133	48.57388	9.24	ILMN_19894	PTHR1	3
PPAN	194.7353	1801.416	9.25	ILMN_25948	PPAN	19
SLC16A13	9.217072	85.42312	9.27	ILMN_7483	SLC16A13	17
GALNT6	7.791725	72.39185	9.29	ILMN_23051	GALNT6	12
CRCT1	0.326494	3.034246	9.29	ILMN_20027	CRCT1	1
FKBP14	101.9659	953.9438	9.36	ILMN_18132	FKBP14	7
ZFYVE27	86.3929	810.2574	9.38	ILMN_3015	ZFYVE27	10
ETS2	768.9476	7221.277	9.39	ILMN_21293	ETS2	21
PTCH1	9.730213	91.45061	9.4	ILMN_18640	PTCH1	9
MGC20983	78.68009	739.9391	9.4	ILMN_26137	MGC20983	19
CIZ1	16.87436	159.4243	9.45	ILMN_10927	CIZ1	9
ADK	9.203711	87.06892	9.46	ILMN_29192	ADK	10
FLJ46082	33.3139	315.3964	9.47	ILMN_25383	FLJ46082	9
C16orf84	26.63674	252.737	9.49	ILMN_27759	C16ORF84	16
ALDH3B2	3.626334	34.41378	9.49	ILMN_2557	ALDH3B2	11
C1orf35	90.60603	861.7542	9.51	ILMN_28904	C1ORF35	1
ASAH2B	13.07448	124.3858	9.51	ILMN_176170	ASAH2B	10
DLC1	2.355806	22.41537	9.51	ILMN_10230	DLC1	8
CCDC88B	17.13842	163.0903	9.52	ILMN_137122	CCDC88B	11
SLC43A2	285.5106	2717.44	9.52	ILMN_26932	SLC43A2	17
VSIG8	32.49463	309.6947	9.53	ILMN_11464	VSIG8	1
FAM184A	59.68407	568.9404	9.53	ILMN_1091	FAM184A	6
MARK2	17.29672	164.9204	9.53	ILMN_138955	MARK2	11
NES	134.8634	1292.662	9.58	ILMN_3947	NES	1
ZNF687	83.05343	798.8956	9.62	ILMN_24389	ZNF687	1
ZFP41	8.307719	80.06534	9.64	ILMN_137505	ZFP41	8
ZBTB12	14.03398	135.4539	9.65	ILMN_640	ZBTB12	6
ETS1	278.9893	2695.179	9.66	ILMN_173009	ETS1	11
PRODH	308.4303	2986.73	9.68	ILMN_17486	PRODH	22
ARHGEF10I	18.76553	181.7321	9.68	ILMN_6822	ARHGEF10L	1
IL34	10.89622	105.7275	9.7	ILMN_5634	IL34	16
TTC9C	82.72047	807.3983	9.76	ILMN_5250	TTC9C	11

VPS72	238.6602	2333.683	9.78	ILMN_17901	VPS72	1
WNK4	81.507	799.0974	9.8	ILMN_7644	WNK4	17
BHLHB8	2.25076	22.06917	9.81	ILMN_27959	BHLHB8	7
FAM50B	222.8126	2187.454	9.82	ILMN_3693	FAM50B	6
PRKX	76.71832	754.7645	9.84	ILMN_167650	PRKX	X
CCDC64	1.244694	12.25696	9.85	ILMN_775	CCDC64	12
LINCR	67.25848	666.6124	9.91	ILMN_162693	LINCR	2
LOC201164	135.1017	1342.509	9.94	ILMN_28101	LOC201164	17
FOXJ1	12.74342	126.8136	9.95	ILMN_13823	FOXJ1	17
PLGLB1	18.80373	187.3808	9.97	ILMN_14164	PLGLB1	2
HTATIP	1.167187	11.65648	9.99	ILMN_3027	HTATIP	11
6-Sep	44.43369	443.7761	9.99	ILMN_413	6-Sep	X
ATP8B3	92.33757	927.9435	10.05	ILMN_25622	ATP8B3	19
TDRD5	35.05964	352.3803	10.05	ILMN_28461	TDRD5	1
TTC15	46.36065	466.3543	10.06	ILMN_21565	TTC15	2
ARHGAP26	47.93308	482.8584	10.07	ILMN_138719	ARHGAP26	5
PRF1	9.251883	93.20124	10.07	ILMN_28175	PRF1	10
ZNF324	39.00415	394.2765	10.11	ILMN_29920	ZNF324	19
RAPGEFL1	113.5632	1152.93	10.15	ILMN_20958	RAPGEFL1	17
PDIA2	2.85126	29.0764	10.2	ILMN_2281	PDIA2	16
DLX1	146.2542	1504.447	10.29	ILMN_10991	DLX1	2
RFX2	29.03149	298.8241	10.29	ILMN_4417	RFX2	19
ARVCF	2.349592	24.21534	10.31	ILMN_7739	ARVCF	22
ST6GALNAC	45.46439	469.1092	10.32	ILMN_137665	ST6GALNAC1	17
NRN1L	4.032497	41.61232	10.32	ILMN_8454	NRN1L	16
PPP1R1B	345.078	3579.737	10.37	ILMN_11681	PPP1R1B	17
RTF1	41.30761	428.976	10.38	ILMN_1866	RTF1	15
FCGBP	164.4662	1708.685	10.39	ILMN_181020	FCGBP	19
VPS39	63.58688	662.6158	10.42	ILMN_5610	VPS39	15
F2R	880.2919	9177.269	10.43	ILMN_165617	F2R	5
NME3	425.3752	4435.931	10.43	ILMN_23571	NME3	16
MAF1	44.25718	464.5953	10.5	ILMN_19711	MAF1	8
PCBP3	12.32492	129.3991	10.5	ILMN_9338	PCBP3	21
TMEM89	5.582386	58.67392	10.51	ILMN_17371	TMEM89	3
C9orf66	202.9837	2135.499	10.52	ILMN_3927	C9ORF66	9
CTAG1A	0.747311	7.91451	10.59	ILMN_22541	CTAG1A	X
CRAT	98.82864	1047.296	10.6	ILMN_21374	CRAT	9
PROM1	1227.929	13015.4	10.6	ILMN_21827	PROM1	4
ARNT2	90.27193	958.1911	10.61	ILMN_13881	ARNT2	15
FCRL2	0.379147	4.045781	10.67	ILMN_3325	FCRL2	1
EBF3	126.8547	1354.245	10.68	ILMN_986	EBF3	10
NRXN2	108.1144	1155.296	10.69	ILMN_25233	NRXN2	11
IQGAP2	25.71608	275.0995	10.7	ILMN_13961	IQGAP2	5
CHST13	318.5249	3418.565	10.73	ILMN_8583	CHST13	3
OCA2	117.1087	1257.11	10.73	ILMN_8155	OCA2	15
SLC7A8	34.19363	367.534	10.75	ILMN_22403	SLC7A8	14
SNHG3-RC(	7.855078	84.52283	10.76	ILMN_167484	SNHG3-RCC1	1

CCDC28B	35.93039	388.3369	10.81	ILMN_26263	CCDC28B	1
LIPC	0.906449	9.797298	10.81	ILMN_182953	LIPC	15
HIST2H4A	8.12297	87.86098	10.82	ILMN_22069	HIST2H4A	1
PRX	2.001107	21.68133	10.83	ILMN_26947	PRX	19
SLC22A18	386.8417	4192.578	10.84	ILMN_18647	SLC22A18	11
ALG12	3.022158	32.93289	10.9	ILMN_19759	ALG12	22
RAB4B	31.34723	341.7453	10.9	ILMN_25106	RAB4B	19
C12orf36	194.5816	2141.71	11.01	ILMN_1264	C12ORF36	12
CALR3	3.604244	39.92009	11.08	ILMN_20160	CALR3	19
C21orf90	9.801606	108.6737	11.09	ILMN_23114	C21ORF90	21
RASIP1	130.7278	1451.025	11.1	ILMN_25004	RASIP1	19
LRAP	6.316808	70.54835	11.17	ILMN_22173	LRAP	5
ANKRD16	115.9816	1297.283	11.19	ILMN_3845	ANKRD16	10
C16orf14	29.44218	330.1962	11.22	ILMN_9509	C16ORF14	16
ZNF521	193.0792	2167.095	11.22	ILMN_22533	ZNF521	18
VSTM2L	0.570784	6.407334	11.23	ILMN_20968	VSTM2L	20
PKNOX1	6.455498	72.80827	11.28	ILMN_29895	PKNOX1	21
ZNF549	1.086512	12.28363	11.31	ILMN_12970	ZNF549	19
SOCS3	0.341703	3.881481	11.36	ILMN_167297	SOCS3	17
GLS2	38.91741	442.6208	11.37	ILMN_2822	GLS2	12
GALIG	27.5903	313.8853	11.38	ILMN_26595	GALIG	14
CCDC17	13.20965	150.5388	11.4	ILMN_9579	CCDC17	1
TBXAS1	4.502741	51.34124	11.4	ILMN_23430	TBXAS1	7
DIS3L2	16.67839	190.9593	11.45	ILMN_24690	DIS3L2	2
FOXD3	4.910762	56.26582	11.46	ILMN_12222	FOXD3	1
EFNB1	244.937	2811.237	11.48	ILMN_9793	EFNB1	X
NDUFA4L2	134.2487	1541.867	11.49	ILMN_11091	NDUFA4L2	12
RDH16	29.01362	334.1345	11.52	ILMN_18171	RDH16	12
EEFSEC	31.62307	365.3422	11.55	ILMN_18983	EEFSEC	3
SMC1A	57.18486	661.4203	11.57	ILMN_19149	SMC1A	X
C9orf37	28.17378	326.3712	11.58	ILMN_12183	C9ORF37	9
AGFG2	8.364461	96.98646	11.6	ILMN_28557	AGFG2	7
SLC25A24	10.58083	122.7659	11.6	ILMN_15753	SLC25A24	1
TEX10	45.48339	527.7634	11.6	ILMN_25785	TEX10	9
MYO1F	54.22013	632.6275	11.67	ILMN_4379	MYO1F	19
XRCC2	2.796265	32.64342	11.67	ILMN_23276	XRCC2	7
WNK1	32.72603	382.4688	11.69	ILMN_6043	WNK1	12
GRPR	0.952594	11.14137	11.7	ILMN_176938	GRPR	X
RHOV	1.163744	13.62176	11.71	ILMN_27155	RHOV	15
RGL2	203.3213	2387.534	11.74	ILMN_5005	RGL2	6
DOHH	0.867818	10.22033	11.78	ILMN_22212	DOHH	19
HELB	12.50245	147.2933	11.78	ILMN_15253	HELB	12
TRIM31	6.499524	76.62847	11.79	ILMN_22919	TRIM31	6
DYRK1B	30.25032	357.2991	11.81	ILMN_12913	DYRK1B	19
HIST1H4H	61.88877	731.9333	11.83	ILMN_30163	HIST1H4H	6
CYorf14	23.13325	274.4778	11.87	ILMN_8952	CYORF14	Y
SLCO6A1	2.922091	34.70451	11.88	ILMN_15327	SLCO6A1	5

CTGLF1	7.884645	93.84391	11.9	ILMN_22934	CTGLF1	10
FGL1	88.19639	1050.857	11.91	ILMN_2104	FGL1	8
RMND5A	53.05122	633.8383	11.95	ILMN_16174	RMND5A	2
PELI1	906.8707	10895.42	12.01	ILMN_11771	PELI1	2
MMP11	67.3544	809.4979	12.02	ILMN_16214	MMP11	22
DSG3	69.67582	839.3528	12.05	ILMN_1906	DSG3	18
INF2	406.9761	4903.562	12.05	ILMN_137125	INF2	14
STON2	5.951618	72.00123	12.1	ILMN_13642	STON2	14
ST8SIA6	13.9186	169.0626	12.15	ILMN_19943	ST8SIA6	10
THPO	3.763008	45.89746	12.2	ILMN_2370	THPO	3
HOXB3	28.95451	354.7058	12.25	ILMN_137307	HOXB3	17
TBX1	12.70427	156.0773	12.29	ILMN_27549	TBX1	22
C14orf172	68.99734	847.8891	12.29	ILMN_11479	C14ORF172	14
MYBPC1	18.87259	231.949	12.29	ILMN_29899	MYBPC1	12
ZNF30	64.4063	794.7982	12.34	ILMN_8558	ZNF30	19
MNS1	247.805	3060.48	12.35	ILMN_8027	MNS1	15
NPAS1	71.60852	884.7823	12.36	ILMN_5949	NPAS1	19
C10orf55	0.444111	5.510311	12.41	ILMN_1489	C10ORF55	10
ABCC6	5.810988	72.28398	12.44	ILMN_175756	ABCC6	16
MYOM2	13.03685	162.3061	12.45	ILMN_9751	MYOM2	8
CISH	66.92418	835.7386	12.49	ILMN_10462	CISH	3
LRRC8D	94.55705	1186.173	12.54	ILMN_16484	LRRC8D	1
NLP	60.78465	765.3191	12.59	ILMN_20940	NLP	20
HBA2	91.0621	1148.041	12.61	ILMN_10268	HBA2	16
MUTYH	66.3617	838.9982	12.64	ILMN_2374	MUTYH	1
GPHA2	9.357157	118.377	12.65	ILMN_20130	GPHA2	11
EPHA3	4.490993	56.85606	12.66	ILMN_10375	EPHA3	3
FLJ41649	1.139746	14.47287	12.7	ILMN_26912	FLJ41649	
C20orf118	64.67502	821.4246	12.7	ILMN_22654	C20ORF118	20
HOXB8	123.6697	1570.782	12.7	ILMN_138137	HOXB8	17
GRHL3	116.8066	1487.915	12.74	ILMN_27092	GRHL3	1
WASF2	27.76434	354.8679	12.78	ILMN_5099	WASF2	1
RILPL2	77.56445	991.5031	12.78	ILMN_2260	RILPL2	12
S1PR4	17.72229	226.5893	12.79	ILMN_139127	S1PR4	19
EIF4EBP1	61.85378	791.1528	12.79	ILMN_6626	EIF4EBP1	8
SLC37A1	36.78771	470.9743	12.8	ILMN_28349	SLC37A1	21
CEACAM5	60.15495	770.2316	12.8	ILMN_16092	CEACAM5	19
CD80	0.168035	2.156079	12.83	ILMN_2131	CD80	3
NPHP4	100.4051	1289.828	12.85	ILMN_17800	NPHP4	1
FABP5L3	22.66724	291.3125	12.85	ILMN_39358	FABP5L3	7
FAM84A	1.551754	19.96082	12.86	ILMN_4138	FAM84A	2
DAB2IP	6.501101	84.23015	12.96	ILMN_20457	DAB2IP	9
TMEM186	16.95225	220.0351	12.98	ILMN_30047	TMEM186	16
PILRA	100.5527	1306.381	12.99	ILMN_27709	PILRA	7
DIP2C	23.83014	309.7606	13	ILMN_16576	DIP2C	10
ZNF503	75.0218	977.0511	13.02	ILMN_2048	ZNF503	10
MAP1A	29.23086	381.3444	13.05	ILMN_13521	MAP1A	15

OSR2	59.03193	773.6957	13.11	ILMN_22030	OSR2	
GOLGA1	16.65646	218.3892	13.11	ILMN_13164	GOLGA1	9
ALPI	6.266406	82.19833	13.12	ILMN_29550	ALPI	2
RHBDL1	32.43513	425.8262	13.13	ILMN_28423	RHBDL1	16
RDHE2	0.841346	11.0915	13.18	ILMN_11660	RDHE2	8
NCF2	11.14607	147.5845	13.24	ILMN_8146	NCF2	1
CSAG1	18.83887	249.5019	13.24	ILMN_15324	CSAG1	X
FLJ40125	5.000969	66.29256	13.26	ILMN_4621	FLJ40125	19
ADCY4	16.09328	213.5825	13.27	ILMN_26146	ADCY4	14
PBX1	55.97689	748.3288	13.37	ILMN_27588	PBX1	1
DCPS	79.30994	1062.098	13.39	ILMN_24626	DCPS	11
NEK8	30.9268	415.4562	13.43	ILMN_26729	NEK8	17
TTC22	11.83759	159.3605	13.46	ILMN_4344	TTC22	1
RTN4RL1	22.29632	300.1987	13.46	ILMN_11868	RTN4RL1	17
CORO1A	0.778619	10.58012	13.59	ILMN_6768	CORO1A	16
PLSCR1	33.01116	448.784	13.59	ILMN_4441	PLSCR1	3
CORO7	35.34978	481.7475	13.63	ILMN_139130	CORO7	16
NPR3	25.4558	347.0938	13.64	ILMN_21860	NPR3	5
NCOR1	36.20703	494.0899	13.65	ILMN_26533	NCOR1	17
SARM1	53.76287	734.0018	13.65	ILMN_23861	SARM1	17
NPDC1	85.99122	1175.363	13.67	ILMN_5190	NPDC1	9
ZNF607	4.575973	62.75479	13.71	ILMN_42496	ZNF607	19
MAD1L1	86.84686	1192.041	13.73	ILMN_9328	MAD1L1	7
SPTLC3	9.092884	124.9409	13.74	ILMN_7329	SPTLC3	20
NRP2	13.05467	180.4649	13.82	ILMN_6157	NRP2	2
FGD4	4.319426	59.75069	13.83	ILMN_28018	FGD4	12
HOXA6	13.38447	185.6073	13.87	ILMN_16941	HOXA6	7
SH2B2	9.748736	135.2051	13.87	ILMN_23870	SH2B2	7
PLA2G12B	40.1612	557.5598	13.88	ILMN_962	PLA2G12B	10
RG9MTD3	16.3624	227.6624	13.91	ILMN_29468	RG9MTD3	9
HCG9	2.194938	30.6263	13.95	ILMN_3917	HCG9	6
CRLF1	51.92132	724.8782	13.96	ILMN_4898	CRLF1	19
NCOA6IP	61.28791	858.3499	14.01	ILMN_8359	NCOA6IP	8
ZNF500	29.94005	420.8042	14.05	ILMN_12312	ZNF500	16
PON1	3.320619	46.70905	14.07	ILMN_24208	PON1	7
JUB	2.626804	36.95198	14.07	ILMN_21630	JUB	14
REEP4	129.7736	1831.22	14.11	ILMN_1061	REEP4	8
MYBPC2	12.63748	178.36	14.11	ILMN_16419	MYBPC2	19
DRD4	44.52757	628.6931	14.12	ILMN_17354	DRD4	11
RNF126	96.88477	1374	14.18	ILMN_5287	RNF126	19
IHPK3	1.155249	16.3975	14.19	ILMN_139316	IHPK3	6
PLXDC2	68.42486	972.9941	14.22	ILMN_29366	PLXDC2	10
FFAR1	1.880739	26.80942	14.25	ILMN_27779	FFAR1	19
HRASLS2	6.463092	92.20287	14.27	ILMN_28302	HRASLS2	11
GRM4	88.14709	1264.818	14.35	ILMN_11058	GRM4	6
PNPLA7	46.48258	672.898	14.48	ILMN_22221	PNPLA7	9
CTSS	3.980192	57.65057	14.48	ILMN_13149	CTSS	1

RPS7	137.8953	1998.993	14.5	ILMN_10275	RPS7	2
PRTN3	35.64867	517.2286	14.51	ILMN_2363	PRTN3	19
SLC14A2	0.214196	3.109882	14.52	ILMN_19280	SLC14A2	18
UNKL	17.23648	250.319	14.52	ILMN_23459	UNKL	16
DDO	1.913798	27.83759	14.55	ILMN_18804	DDO	6
ARRDC1	78.02293	1138.342	14.59	ILMN_11748	ARRDC1	9
PROC	44.45718	651.2076	14.65	ILMN_20582	PROC	2
LOC340069	0.278943	4.087409	14.65	ILMN_24305	LOC340069	5
TRAF2	140.3875	2062.597	14.69	ILMN_15779	TRAF2	9
KCNH8	128.728	1896.172	14.73	ILMN_25904	KCNH8	3
ARHGAP6	1.183531	17.4819	14.77	ILMN_17180	ARHGAP6	X
GPM6B	53.53852	791.726	14.79	ILMN_23991	GPM6B	X
ZNF114	17.86772	268.9593	15.05	ILMN_3101	ZNF114	19
DNAH2	302.9603	4593.694	15.16	ILMN_13178	DNAH2	17
SLC29A3	1.076593	16.3363	15.17	ILMN_2134	SLC29A3	10
GTF3C4	62.21589	947.5139	15.23	ILMN_7794	GTF3C4	9
FBXL4	4.485037	68.36116	15.24	ILMN_8755	FBXL4	6
BARX2	13.75322	210.0531	15.27	ILMN_20099	BARX2	11
C9orf89	151.8649	2321.086	15.28	ILMN_17058	C9ORF89	9
PRG-3	12.96833	198.5215	15.31	ILMN_25528	PRG-3	9
LOC389816	477.1447	7328.938	15.36	ILMN_182870	LOC389816	9
FOXP1	7.215422	110.9722	15.38	ILMN_21389	FOXP1	3
APLN	5.45082	83.85925	15.38	ILMN_7244	APLN	X
TBX6	1.192864	18.58209	15.58	ILMN_17505	TBX6	16
NKX2-5	46.4305	724.5374	15.6	ILMN_12736	NKX2-5	5
GUCY2D	4.235943	66.14791	15.62	ILMN_24829	GUCY2D	17
AQP6	4.810681	75.56308	15.71	ILMN_137333	AQP6	12
PAPLN	109.7047	1725.036	15.72	ILMN_4286	PAPLN	14
INHBE	2.968631	46.6878	15.73	ILMN_7067	INHBE	12
MACROD1	90.37892	1425.667	15.77	ILMN_13854	MACROD1	11
MADCAM1	1.613205	25.47503	15.79	ILMN_1646	MADCAM1	19
PRDM2	4.364013	68.91865	15.79	ILMN_180797	PRDM2	1
ADAMTS2	17.44993	276.1245	15.82	ILMN_29307	ADAMTS2	5
CKMT2	1.129659	17.9152	15.86	ILMN_8974	CKMT2	5
FAM19A4	0.837134	13.30313	15.89	ILMN_29743	FAM19A4	3
CAMKK2	3.221578	51.29614	15.92	ILMN_9437	CAMKK2	12
MMP15	89.54041	1435.564	16.03	ILMN_16459	MMP15	16
EOMES	33.92444	546.7975	16.12	ILMN_19998	EOMES	3
SLC35D3	96.35657	1553.665	16.12	ILMN_16642	SLC35D3	6
NFKBIL1	3.926376	63.34686	16.13	ILMN_8342	NFKBIL1	6
NUDT17	2.919189	47.1219	16.14	ILMN_5983	NUDT17	1
N-PAC	25.68565	414.7875	16.15	ILMN_13959	N-PAC	16
CCL20	17.22697	280.2113	16.27	ILMN_1789	CCL20	2
TMPRSS3	8.472491	137.8509	16.27	ILMN_20260	TMPRSS3	21
PDK4	42.98855	699.5477	16.27	ILMN_23211	PDK4	7
PIGS	65.95478	1074.123	16.29	ILMN_7735	PIGS	17
ZNF367	61.06242	995.7119	16.31	ILMN_23631	ZNF367	9

CRYBA4	87.17175	1424.509	16.34	ILMN_20721	CRYBA4	22
RPRML	9.797298	160.543	16.39	ILMN_24865	RPRML	17
CNDP1	38.56038	633.3741	16.43	ILMN_28642	CNDP1	18
C9orf41	64.20598	1060.898	16.52	ILMN_13662	C9ORF41	9
GZMM	1.098688	18.17457	16.54	ILMN_528	GZMM	19
TGFBRAP1	54.20218	898.6243	16.58	ILMN_30176	TGFBRAP1	2
RYR2	15.79495	262.5902	16.62	ILMN_23697	RYR2	1
C1QTNF5	10.29469	171.7022	16.68	ILMN_23052	C1QTNF5	11
TMIE	1.433577	23.95211	16.71	ILMN_533	TMIE	3
LOC200810	76.24183	1294.085	16.97	ILMN_651	LOC200810	
TRAF3	13.04345	222.7095	17.07	ILMN_29663	TRAF3	14
ZNF69	8.428915	144.1078	17.1	ILMN_23104	ZNF69	19
DHX35	24.6342	423.428	17.19	ILMN_18259	DHX35	20
PHACS	28.41851	491.7094	17.3	ILMN_3829	PHACS	11
UNC93A	60.05074	1039.802	17.32	ILMN_25955	UNC93A	6
PRKCG	9.560624	165.7374	17.34	ILMN_23304	PRKCG	19
IFITM1	829.7617	14541.66	17.53	ILMN_24466	IFITM1	11
EDG7	18.82444	330.3277	17.55	ILMN_18518	EDG7	1
SLC45A4	29.03505	510.4509	17.58	ILMN_168340	SLC45A4	8
PINX1	33.29966	586.9904	17.63	ILMN_14670	PINX1	8
NPSR1	53.36288	946.4102	17.74	ILMN_16921	NPSR1	7
PPP1R1A	43.51792	775.0316	17.81	ILMN_17363	PPP1R1A	12
B4GALT2	3.83469	68.31567	17.82	ILMN_3553	B4GALT2	1
C4BPB	15.47044	275.6738	17.82	ILMN_21736	C4BPB	1
RANBP10	2.569239	45.78352	17.82	ILMN_21091	RANBP10	16
TIMM44	104.8579	1877.535	17.91	ILMN_29473	TIMM44	19
WNK2	32.31434	579.4327	17.93	ILMN_20038	WNK2	9
ANKAR	27.44711	492.4205	17.94	ILMN_176429	ANKAR	2
ACSBG1	37.55411	674.2964	17.96	ILMN_2697	ACSBG1	15
PDE10A	2.094586	37.76784	18.03	ILMN_22388	PDE10A	6
KIAA1553	60.48839	1093.74	18.08	ILMN_168708	KIAA1553	6
IFT140	14.06609	255.6284	18.17	ILMN_3437	IFT140	16
AQP1	0.346169	6.312066	18.23	ILMN_24349	AQP1	7
GPRIN3	0.825494	15.06871	18.25	ILMN_8803	GPRIN3	4
GYLTL1B	0.969139	17.71306	18.28	ILMN_23778	GYLTL1B	11
ANKS3	61.60429	1126.26	18.28	ILMN_25038	ANKS3	16
AGR2	15.17153	277.9749	18.32	ILMN_15860	AGR2	7
NFKBIL2	1.74806	32.03593	18.33	ILMN_11110	NFKBIL2	8
CDKN2D	80.87885	1482.643	18.33	ILMN_28866	CDKN2D	19
MRPL28	58.88153	1087.458	18.47	ILMN_22546	MRPL28	16
LARGE	141.2303	2625.143	18.59	ILMN_5209	LARGE	22
STS	33.41158	624.6558	18.7	ILMN_25374	STS	X
ST3GAL4	165.9092	3105.543	18.72	ILMN_19390	ST3GAL4	11
DLL1	204.2546	3823.828	18.72	ILMN_1802	DLL1	6
CSF2RA	33.25584	630.2518	18.95	ILMN_5061	CSF2RA	Y
KIAA0367	140.3244	2663.296	18.98	ILMN_23214	KIAA0367	9
USH1C	24.75997	470.4244	19	ILMN_30277	USH1C	11

ANO4	32.90929	625.3271	19	ILMN_173162	ANO4	12
C19orf47	36.38847	691.7385	19.01	ILMN_11163	C19ORF47	19
MGC42367	351.5178	6695.892	19.05	ILMN_8681	MGC42367	2
ALOX15B	1.075	20.54329	19.11	ILMN_6877	ALOX15B	17
CEACAM7	30.51054	583.804	19.13	ILMN_14231	CEACAM7	19
NTRK2	8.801978	168.6293	19.16	ILMN_4716	NTRK2	9
HKDC1	145.0702	2785.136	19.2	ILMN_4255	HKDC1	10
CLRN3	83.0134	1601.866	19.3	ILMN_27145	CLRN3	10
GLIS2	15.02798	290.4228	19.33	ILMN_3031	GLIS2	16
CDKN2C	8.602597	166.4539	19.35	ILMN_18926	CDKN2C	1
C11orf49	14.73576	287.489	19.51	ILMN_29991	C11ORF49	11
C17orf28	14.11514	275.4056	19.51	ILMN_5120	C17ORF28	17
GHRL	0.811177	15.86852	19.56	ILMN_19385	GHRL	3
IFIT3	72.43916	1423.386	19.65	ILMN_22925	IFIT3	10
GALNT9	1.756235	34.56654	19.68	ILMN_16714	GALNT9	12
IL12RB1	0.247316	4.90585	19.84	ILMN_4594	IL12RB1	19
SMPD2	53.27496	1065.1	19.99	ILMN_2791	SMPD2	6
PRLR	2.062145	41.24965	20	ILMN_22505	PRLR	5
NTRK1	4.871431	97.48964	20.01	ILMN_5114	NTRK1	1
LINGO1	33.56913	671.8899	20.02	ILMN_28172	LINGO1	15
SCN4B	0.62615	12.56027	20.06	ILMN_1870	SCN4B	11
ETHE1	5.497849	110.2878	20.06	ILMN_18269	ETHE1	19
IL2RG	2.610412	52.48917	20.11	ILMN_16342	IL2RG	X
FLJ22662	502.1331	10097.77	20.11	ILMN_27776	FLJ22662	12
NAT8B	23.15965	467.2687	20.18	ILMN_8663	NAT8B	2
C3orf25	6.343671	128.728	20.29	ILMN_23409	C3ORF25	3
TGFBI	233.5548	4745.765	20.32	ILMN_24587	TGFBI	5
NEK4	19.35907	393.4115	20.32	ILMN_6182	NEK4	3
CCDC102A	61.46921	1251.957	20.37	ILMN_12942	CCDC102A	16
IGF1R	29.09192	592.925	20.38	ILMN_16324	IGF1R	15
TCEAL3	1.992883	40.95039	20.55	ILMN_20249	TCEAL3	X
HPDL	204.6889	4207.148	20.55	ILMN_27879	HPDL	1
ZNF652	29.54819	608.3193	20.59	ILMN_3215	ZNF652	17
TNFRSF14	246.5664	5091.958	20.65	ILMN_3329	TNFRSF14	1
CACNA2D4	27.82043	574.7678	20.66	ILMN_15285	CACNA2D4	12
TEPP	2.992973	61.891	20.68	ILMN_21753	TEPP	16
C21orf34	12.9977	270.5206	20.81	ILMN_27527	C21ORF34	21
RASL11B	25.16969	525.0484	20.86	ILMN_7453	RASL11B	4
SSSCA1	18.46082	385.2847	20.87	ILMN_7443	SSSCA1	11
CHP2	3.377379	70.69991	20.93	ILMN_7582	CHP2	16
TRAM1L1	7.354411	154.4619	21	ILMN_22940	TRAM1L1	4
FHDC1	33.43349	703.09	21.03	ILMN_18005	FHDC1	4
FAM26F	0.359226	7.583973	21.11	ILMN_172470	FAM26F	6
C2orf83	0.299864	6.367607	21.24	ILMN_16756	C2ORF83	2
CDH23	4.381487	93.11632	21.25	ILMN_16568	CDH23	10
ZNF575	3.490618	74.83156	21.44	ILMN_5850	ZNF575	19
CIDEC	141.3002	3051.084	21.59	ILMN_174646	CIDEC	3

STX17	10.61341	230.5918	21.73	ILMN_29240	STX17	9
TAPBPL	21.79542	481.2442	22.08	ILMN_21656	TAPBPL	12
PDZD3	11.38113	251.6907	22.11	ILMN_25526	PDZD3	11
NDUFB6	57.2794	1267.672	22.13	ILMN_9342	NDUFB6	9
MFAP5	98.86201	2192.461	22.18	ILMN_19364	MFAP5	12
UNK	9.02902	200.7717	22.24	ILMN_165760	UNK	17
DOC2B	12.34799	274.9538	22.27	ILMN_3915	DOC2B	17|NT_113933.1
TRIM15	83.41891	1864.837	22.36	ILMN_18211	TRIM15	6
ASAH1	52.06301	1174.953	22.57	ILMN_26236	ASAH1	8
EPC2	0.573307	12.94589	22.58	ILMN_26742	EPC2	2
ENG	17.05842	386.823	22.68	ILMN_18184	ENG	9
CCDC52	5.009554	114.4952	22.86	ILMN_23129	CCDC52	3
TRIM74	3.29788	75.40145	22.86	ILMN_5731	TRIM74	7
HYAL2	17.43559	399.038	22.89	ILMN_19286	HYAL2	3
ABTB2	25.69045	589.5943	22.95	ILMN_22109	ABTB2	11
TAS2R49	7.720369	178.2719	23.09	ILMN_6664	TAS2R49	
RCC1	40.40321	940.6647	23.28	ILMN_14892	RCC1	1
JMJD2B	1.381837	32.35996	23.42	ILMN_2379	JMJD2B	19
SIX2	5.741653	134.8634	23.49	ILMN_8167	SIX2	2
CD84	0.097652	2.294808	23.5	ILMN_16790	CD84	1
ZNF83	59.04197	1391.851	23.57	ILMN_23108	ZNF83	19
CLEC7A	0.215061	5.077364	23.61	ILMN_33785	CLEC7A	12
C13orf18	5.108163	120.6605	23.62	ILMN_19053	C13ORF18	13
HNF4G	161.1441	3811.641	23.65	ILMN_12180	HNF4G	8
TBC1D1	21.01397	497.0794	23.65	ILMN_3070	TBC1D1	4
TMED6	42.34304	1002.244	23.67	ILMN_8281	TMED6	16
TM4SF4	12.26923	292.4799	23.84	ILMN_1556	TM4SF4	3
LOC440925	6.422012	153.7194	23.94	ILMN_19833	LOC440925	2
TSPAN8	42.64507	1026.434	24.07	ILMN_578	TSPAN8	12
MAT1A	1.555261	37.44165	24.07	ILMN_15458	MAT1A	10
SOAT2	3.094915	74.55789	24.09	ILMN_3634	SOAT2	12
SLC2A10	16.21449	390.6467	24.09	ILMN_1430	SLC2A10	20
AGXT	1.08009	26.18276	24.24	ILMN_21738	AGXT	2
CBR1	97.53138	2376.43	24.37	ILMN_18628	CBR1	21
NCK2	3.072027	75.09064	24.44	ILMN_163588	NCK2	2
UPK3B	3.784548	92.6996	24.49	ILMN_24270	UPK3B	7
ATP8A1	37.29811	913.7272	24.5	ILMN_7068	ATP8A1	4
SERF1A	2.618436	64.1807	24.51	ILMN_13897	SERF1A	5
MYCN	59.45612	1459.969	24.56	ILMN_178034	MYCN	2
FUT2	0.853378	21.16813	24.81	ILMN_5822	FUT2	19
KRT20	70.35029	1749.175	24.86	ILMN_161882	KRT20	17
ODZ3	219.0831	5463.67	24.94	ILMN_179907	ODZ3	4
DOC2A	12.82842	322.3886	25.13	ILMN_15589	DOC2A	16
PIWIL2	8.63949	217.3123	25.15	ILMN_26555	PIWIL2	8
IGSF1	4.972242	125.4529	25.23	ILMN_4473	IGSF1	X
HIST1H2BG	36.19323	920.867	25.44	ILMN_21089	HIST1H2BG	6
NR1H2	6.70263	173.9479	25.95	ILMN_137460	NR1H2	19

DCN	2.581168	67.04523	25.97	ILMN_29913	DCN	12
CATSPER2	0.26622	6.949671	26.11	ILMN_28667	CATSPER2	15
DEGS2	69.0918	1806.123	26.14	ILMN_25949	DEGS2	14
HOXA3	10.48649	275.826	26.3	ILMN_138124	HOXA3	7
GRTP1	133.2638	3506.882	26.32	ILMN_24814	GRTP1	13
ATAD4	99.8395	2640.52	26.45	ILMN_24488	ATAD4	17
UNQ830	2.266872	60.39806	26.64	ILMN_18124	UNQ830	2
SPINK1	3.771747	100.5716	26.66	ILMN_25404	SPINK1	5
BTN2A3	1.969006	52.72094	26.78	ILMN_19904	BTN2A3	6
CASKIN1	8.640175	231.6094	26.81	ILMN_20836	CASKIN1	16
CALB1	19.3212	518.6754	26.84	ILMN_13367	CALB1	8
KLRG2	51.75877	1402.032	27.09	ILMN_38286	KLRG2	7
PRAP1	7.37698	200.6954	27.21	ILMN_15947	PRAP1	10
TCL1B	0.866364	23.6366	27.28	ILMN_30300	TCL1B	14
ABCB8	5.746238	157.0645	27.33	ILMN_16943	ABCB8	7
REEP6	196.6096	5378.315	27.36	ILMN_15192	REEP6	19
PGM5P2	0.322938	8.944704	27.7	ILMN_19104	PGM5P2	9
LRIG1	51.03047	1415.722	27.74	ILMN_3319	LRIG1	3
KLK1	19.13042	539.3322	28.19	ILMN_19429	KLK1	19
APOL6	4.558978	129.6624	28.44	ILMN_138012	APOL6	22
PAX6	4.535755	129.6606	28.59	ILMN_634	PAX6	11
KCNJ4	3.363443	96.45058	28.68	ILMN_14221	KCNJ4	22
GAGE4	7.331597	210.2684	28.68	ILMN_9440	GAGE4	X
ZNF91	8.5723	246.0336	28.7	ILMN_13294	ZNF91	19
PSMB8	173.2501	4990.513	28.81	ILMN_12139	PSMB8	6
TMEM184B	22.49709	652.3781	29	ILMN_10219	TMEM184B	22
OAF	215.0023	6272.631	29.17	ILMN_12751	OAF	11
TPPP3	25.16286	737.7331	29.32	ILMN_754	TPPP3	16
SPNS3	10.52745	310.8047	29.52	ILMN_17661	SPNS3	17
WFDC1	9.990226	299.0909	29.94	ILMN_26086	WFDC1	16
CPNE6	6.247946	187.2481	29.97	ILMN_19559	CPNE6	14
ASCL2	43.34837	1320.729	30.47	ILMN_18051	ASCL2	11
HNF4A	9.902835	303.3011	30.63	ILMN_12529	HNF4A	20
D4S234E	108.2288	3345.361	30.91	ILMN_9299	D4S234E	4
DENND1C	52.4179	1632.141	31.14	ILMN_16949	DENND1C	19
ANKS4B	28.71908	910.8334	31.72	ILMN_9791	ANKS4B	16
ZNF816A	205.8695	6647.985	32.29	ILMN_15160	ZNF816A	19
SLC40A1	1.023333	33.04906	32.3	ILMN_182851	SLC40A1	2
NADSYN1	10.46379	340.7135	32.56	ILMN_22444	NADSYN1	11
CDX1	129.023	4215.785	32.67	ILMN_27843	CDX1	5
GLTPD1	1.209253	39.82624	32.93	ILMN_180743	GLTPD1	1
FLJ14107	2.026118	66.76125	32.95	ILMN_603	FLJ14107	
AIM1L	1.215343	40.05368	32.96	ILMN_45818	AIM1L	1
ZNF268	18.00203	594.0287	33	ILMN_4870	ZNF268	12
FUSIP1	36.25683	1203.445	33.19	ILMN_30145	FUSIP1	1
LXN	91.40157	3035.315	33.21	ILMN_10044	LXN	3
SLC1A7	1.014369	33.72925	33.25	ILMN_26632	SLC1A7	1

HGD	44.72254	1494.07	33.41	ILMN_7177	HGD	3
SORCS1	9.641303	325.1387	33.72	ILMN_4541	SORCS1	10
ZNF598	102.6295	3470.714	33.82	ILMN_24766	ZNF598	16
MAP1LC3A	45.87187	1553.062	33.86	ILMN_25755	MAP1LC3A	20
SEMA4G	3.02753	102.6295	33.9	ILMN_22778	SEMA4G	10
CAPN9	86.67075	2948.939	34.02	ILMN_934	CAPN9	1
TCN2	14.71916	503.6277	34.22	ILMN_6136	TCN2	22
PDE3A	11.73405	403.3903	34.38	ILMN_5575	PDE3A	12
ODF3L2	11.50018	395.6451	34.4	ILMN_17282	ODF3L2	19
RSAD2	6.383257	220.1815	34.49	ILMN_138156	RSAD2	2
ZNF136	13.55866	471.6573	34.79	ILMN_7813	ZNF136	
NRIP1	53.63551	1876.209	34.98	ILMN_4339	NRIP1	21
TPPP	4.514782	158.2892	35.06	ILMN_655	TPPP	5
DLL3	48.34777	1708.778	35.34	ILMN_21363	DLL3	19
SLC23A1	1.964139	69.63219	35.45	ILMN_3326	SLC23A1	5
IFI27	142.5522	5081.082	35.64	ILMN_17548	IFI27	14
LOC39990(	19.00016	683.2802	35.96	ILMN_15719	LOC399900	11
ACRBP	4.710326	169.5141	35.99	ILMN_22648	ACRBP	12
ADAMTS6	0.516746	18.6815	36.15	ILMN_13736	ADAMTS6	5
C17orf73	34.16829	1236.237	36.18	ILMN_25284	C17ORF73	17
RARRES3	86.74757	3170.836	36.55	ILMN_1164	RARRES3	11
COMMD4	113.3268	4204.99	37.1	ILMN_33223	COMMD4	15
PIP5K1B	16.79063	624.7824	37.21	ILMN_5052	PIP5K1B	9
SLC26A3	0.913405	34.09851	37.33	ILMN_21833	SLC26A3	7
REG4	44.35491	1658.019	37.38	ILMN_8328	REG4	1
POMT1	1.652085	63.02107	38.15	ILMN_18145	POMT1	9
TCF4	1.671234	65.22928	39.03	ILMN_12126	TCF4	18
HRK	13.96821	545.5273	39.05	ILMN_9421	HRK	12
TMEM63A	79.96239	3130.246	39.15	ILMN_932	TMEM63A	1
TCF2	8.877125	348.0536	39.21	ILMN_21720	TCF2	17
CEP97	1.674214	66.00218	39.42	ILMN_10437	CEP97	3
CENTD2	1.176527	46.39934	39.44	ILMN_12595	CENTD2	11
MAPK15	58.21009	2311.922	39.72	ILMN_13601	MAPK15	8
OR2T1	0.18955	7.539054	39.77	ILMN_19442	OR2T1	1
CALML3	1.253004	50.19765	40.06	ILMN_20293	CALML3	10
GNAO1	2.621887	105.9851	40.42	ILMN_3743	GNAO1	16
C7orf33	31.74582	1301.316	40.99	ILMN_10420	C7ORF33	7
UBA7	3.625981	149.1091	41.12	ILMN_22067	UBA7	3
NEDD9	18.71224	769.6446	41.13	ILMN_137978	NEDD9	6
ZMAT3	0.269202	11.09672	41.22	ILMN_9027	ZMAT3	3
RNF44	57.68698	2408.393	41.75	ILMN_20290	RNF44	5
ELF5	23.53257	993.2526	42.21	ILMN_13737	ELF5	11
CHI3L2	17.2588	729.0151	42.24	ILMN_1036	CHI3L2	1
BATF2	16.55201	713.2513	43.09	ILMN_10220	BATF2	11
DACH1	54.90359	2410.872	43.91	ILMN_19600	DACH1	13
TUSC3	1.865059	82.14027	44.04	ILMN_2352	TUSC3	8
IZUMO1	0.385033	17.03848	44.25	ILMN_5623	IZUMO1	19

CLOCK	0.782956	34.99572	44.7	ILMN_15530	CLOCK	4
CLEC4D	0.138246	6.198254	44.83	ILMN_30135	CLEC4D	12
NSUN5B	0.58885	26.48923	44.98	ILMN_32951	NSUN5B	7
RDH13	57.49864	2593.94	45.11	ILMN_10594	RDH13	19
C15orf48	14.83018	672.256	45.33	ILMN_2226	C15ORF48	15
OAS1	32.13896	1457.522	45.35	ILMN_2717	OAS1	12
ACHE	11.02567	500.7375	45.42	ILMN_43873	ACHE	7
TMEM45B	20.59341	940.4139	45.67	ILMN_15699	TMEM45B	11
ATCAY	0.068156	3.117504	45.74	ILMN_27014	ATCAY	19
DMBT1	2.476618	113.5632	45.85	ILMN_5328	DMBT1	10
C4orf35	2.235816	102.6695	45.92	ILMN_136996	C4ORF35	4
CLDN4	0.41421	19.08793	46.08	ILMN_23316	CLDN4	7
SLC7A7	25.53177	1177.589	46.12	ILMN_16478	SLC7A7	14
SYNGR4	17.78939	825.4533	46.4	ILMN_20845	SYNGR4	19
EPS8L3	8.668395	406.7801	46.93	ILMN_23672	EPS8L3	1
ZNF137	8.038833	378.985	47.14	ILMN_138821	ZNF137	
DAGLA	23.15173	1094.373	47.27	ILMN_2615	DAGLA	11
ZNF614	36.48435	1730.045	47.42	ILMN_715	ZNF614	19
CCDC123	9.957741	473.1645	47.52	ILMN_19249	CCDC123	19
FMO5	3.386282	161.5578	47.71	ILMN_12373	FMO5	1
SLC15A1	21.7041	1047.512	48.26	ILMN_11327	SLC15A1	13
TMEM82	10.58562	515.5491	48.7	ILMN_18250	TMEM82	1
ZNF260	112.4592	5480.352	48.73	ILMN_6166	ZNF260	19
ANKRD56	0.76559	37.39474	48.84	ILMN_162776	ANKRD56	4
ZNF649	14.45879	707.012	48.9	ILMN_15045	ZNF649	19
NOXO1	16.60336	819.3944	49.35	ILMN_15286	NOXO1	16
ZNF20	41.43285	2049.658	49.47	ILMN_10297	ZNF20	19
NRTN	69.77982	3462.767	49.62	ILMN_25657	NRTN	19
PIK3IP1	19.01835	951.3393	50.02	ILMN_15026	PIK3IP1	22
NUBP2	32.51154	1682.564	51.75	ILMN_10701	NUBP2	16
BTBD16	44.22974	2309.326	52.21	ILMN_23857	BTBD16	10
COL5A1	0.646775	34.1397	52.78	ILMN_139036	COL5A1	9
LOC60649[illegible]	4.700032	249.4818	53.08	ILMN_4186	LOC606495	1
MESDC1	0.446911	23.76498	53.18	ILMN_18570	MESDC1	15
NFE2L1	96.51402	5216.908	54.05	ILMN_13955	NFE2L1	17
C1orf183	1.145005	61.9529	54.11	ILMN_9599	C1ORF183	1
DNHD2	2.143858	118.8913	55.46	ILMN_6160	DNHD2	
HNMT	14.49552	806.3878	55.63	ILMN_179818	HNMT	2
ZNF331	0.301438	16.82583	55.82	ILMN_28697	ZNF331	19
VASP	1.341576	75.33281	56.15	ILMN_28263	VASP	19
AGR3	19.89758	1131.369	56.86	ILMN_20719	AGR3	7
GPR124	5.884462	334.8618	56.91	ILMN_16235	GPR124	8
PRB3	1.98865	113.8022	57.23	ILMN_180495	PRB3	12
PLA2G2A	155.8462	9008.913	57.81	ILMN_13069	PLA2G2A	1
ALOX5	18.93693	1100.122	58.09	ILMN_2997	ALOX5	10
ATP9B	9.162422	532.9504	58.17	ILMN_8061	ATP9B	18
HIST1H2AE	40.68477	2366.705	58.17	ILMN_1822	HIST1H2AE	6

THAP4	6.482443	380.0753	58.63	ILMN_8784	THAP4	2
SELM	42.78265	2513.051	58.74	ILMN_1005	SELM	22
LRRC45	22.1255	1301.584	58.83	ILMN_10648	LRRC45	17
LGALS12	5.821346	342.8392	58.89	ILMN_26017	LGALS12	11
MLL4	12.55346	740.2246	58.97	ILMN_28047	MLL4	19
CEP27	20.0328	1186.642	59.23	ILMN_15131	CEP27	15
OSTbeta	3.832522	228.0933	59.52	ILMN_30037	OSTBETA	15
CLDND2	105.478	6325.353	59.97	ILMN_15940	CLDND2	19
LDB2	2.12845	127.7782	60.03	ILMN_2220	LDB2	4
HLA-F	5.705681	347.9045	60.98	ILMN_3012	HLA-F	6
CREG2	21.60016	1329.633	61.56	ILMN_4426	CREG2	2
NGF	0.09297	5.749192	61.84	ILMN_18062	NGF	1
KIF18B	0.888461	55.103	62.02	ILMN_172485	KIF18B	17
FOXA2	26.79882	1668.101	62.25	ILMN_2357	FOXA2	20
NPTX2	54.62694	3400.997	62.26	ILMN_22638	NPTX2	7
EPN2	8.798129	551.5392	62.69	ILMN_21232	EPN2	17
P8	7.983866	503.7695	63.1	ILMN_26003	P8	16
NECAB2	16.12798	1021.944	63.36	ILMN_12534	NECAB2	16
C3orf70	7.124422	454.0115	63.73	ILMN_175119	C3ORF70	3
EPHB1	61.30107	3908.116	63.75	ILMN_5567	EPHB1	3
HCRT	5.224772	336.9203	64.49	ILMN_25303	HCRT	17
LRRC26	42.13764	2798.395	66.41	ILMN_41107	LRRC26	9
ACE2	1.173752	79.8319	68.01	ILMN_26080	ACE2	X
ZNF300	51.39768	3505.143	68.2	ILMN_23333	ZNF300	5
ABP1	13.55645	927.1451	68.39	ILMN_4987	ABP1	7
WNT1	0.885809	61.03716	68.91	ILMN_22389	WNT1	12
PLAC8	57.89442	4001.683	69.12	ILMN_17809	PLAC8	4
PC-3	0.322968	22.49709	69.66	ILMN_168723	PC-3	9
ATP6V1C2	13.19338	925.403	70.14	ILMN_42208	ATP6V1C2	2
AXIN2	304.0267	21402.23	70.4	ILMN_26857	AXIN2	17
VENTXP7	0.03819	2.708234	70.91	ILMN_925	VENTXP7	3
ZNF695	18.57763	1335.544	71.89	ILMN_11705	ZNF695	1
EHF	76.05241	5520.221	72.58	ILMN_16913	EHF	11
VWCE	15.65546	1145.886	73.19	ILMN_18416	VWCE	11
FAM158A	13.57794	1010.243	74.4	ILMN_6095	FAM158A	14
LAMA4	11.18437	834.8129	74.64	ILMN_4021	LAMA4	6
NPFF	0.333469	25.1245	75.34	ILMN_11221	NPFF	12
TRMU	9.332028	706.8647	75.75	ILMN_137256	TRMU	22
SLIT1	0.554448	42.18534	76.09	ILMN_17043	SLIT1	10
FCGRT	103.3733	7881.395	76.24	ILMN_17135	FCGRT	19
SLC43A1	0.473276	36.35929	76.82	ILMN_20441	SLC43A1	11
RTN4RL2	0.695833	53.47557	76.85	ILMN_22507	RTN4RL2	11
DOCK8	19.62578	1509.677	76.92	ILMN_1726	DOCK8	9
PLEKHA4	7.848195	613.9988	78.23	ILMN_5126	PLEKHA4	19
RXFP4	0.242145	19.16504	79.15	ILMN_16937	RXFP4	1
BRUNOL5	2.005221	161.3132	80.45	ILMN_8461	BRUNOL5	19
CDH17	90.82848	7364.575	81.08	ILMN_14774	CDH17	8

TMPRSS2	75.40145	6136.511	81.38	ILMN_13877	TMPRSS2	21
C14orf49	0.9465	77.33706	81.71	ILMN_8022	C14ORF49	14
MORN3	2.139057	174.9789	81.8	ILMN_4514	MORN3	12
CAMKV	17.53514	1457.001	83.09	ILMN_136941	CAMKV	3
C10orf93	3.773067	313.5704	83.11	ILMN_139313	C10ORF93	
IL1R2	10.93143	913.5078	83.57	ILMN_6394	IL1R2	2
INHBB	26.87621	2256.788	83.97	ILMN_7166	INHBB	2
MXRA5	18.47421	1555.307	84.19	ILMN_29922	MXRA5	X
SOX2	15.93087	1342.972	84.3	ILMN_171554	SOX2	3
NOSTRIN	38.4978	3320.915	86.26	ILMN_10508	NOSTRIN	2
ABCA1	23.21292	2027.527	87.34	ILMN_21049	ABCA1	9
ZNF28	9.96152	874.9276	87.83	ILMN_8130	ZNF28	19
GAL3ST1	1.530926	135.1411	88.27	ILMN_28424	GAL3ST1	22
LYZ	43.69485	3871.794	88.61	ILMN_4879	LYZ	12
MYO1A	65.93996	5847.706	88.68	ILMN_9275	MYO1A	12
MGC50722	6.546609	584.8334	89.33	ILMN_29957	MGC50722	
EPB41L3	43.67072	3960.826	90.7	ILMN_14465	EPB41L3	18
OVOL1	1.051902	96.27125	91.52	ILMN_14373	OVOL1	11
SLC44A4	20.65364	1893.578	91.68	ILMN_8031	SLC44A4	6
ALDH1A1	70.78556	6515.324	92.04	ILMN_177898	ALDH1A1	9
ADORA2A	1.794986	166.9599	93.01	ILMN_8227	ADORA2A	22
GIPC2	31.07404	2897.35	93.24	ILMN_168071	GIPC2	1
NRG3	0.109354	10.31268	94.31	ILMN_13343	NRG3	10
PELI2	5.789031	554.4917	95.78	ILMN_138073	PELI2	14
FSTL5	0.23589	23.21292	98.41	ILMN_2509	FSTL5	4
ERN2	23.07415	2342.641	101.53	ILMN_2550	ERN2	16
TMPRSS4	6.842782	705.3517	103.08	ILMN_29020	TMPRSS4	11
RPS4Y1	27.52694	2863.723	104.03	ILMN_8579	RPS4Y1	Y
LOC440157	21.97846	2297.139	104.52	ILMN_20657	LOC440157	14
METRN	6.222208	655.611	105.37	ILMN_25836	METRN	16
CYP2W1	5.043413	538.312	106.74	ILMN_19493	CYP2W1	7
ACY3	2.058517	219.9156	106.83	ILMN_28192	ACY3	11
MT1M	16.03053	1713.687	106.9	ILMN_25245	MT1M	16
HOXB9	5.73768	614.2275	107.05	ILMN_137452	HOXB9	17
STAT5A	2.462564	265.0344	107.63	ILMN_10520	STAT5A	17
ProSAPiP1	21.87175	2354.76	107.66	ILMN_638	PROSAPIP1	20
CGI-96	2.572982	280.1055	108.86	ILMN_12816	CGI-96	
TNFRSF1B	3.430366	374.2765	109.11	ILMN_19893	TNFRSF1B	1
PAPSS2	7.82636	866.8304	110.76	ILMN_9981	PAPSS2	10
SORBS2	6.61355	738.7047	111.7	ILMN_24864	SORBS2	4
HIST1H3G	1.608643	179.8674	111.81	ILMN_29118	HIST1H3G	6
SLC13A5	11.24389	1308.751	116.4	ILMN_24584	SLC13A5	17
TRIM44	0.735622	86.89785	118.13	ILMN_5006	TRIM44	11
ADAMTSL4	4.968101	588.3683	118.43	ILMN_138028	ADAMTSL4	1
PRTFDC1	7.097993	842.7615	118.73	ILMN_19592	PRTFDC1	10
IL17RA	3.906149	464.1034	118.81	ILMN_19124	IL17RA	22
C1orf92	1.873835	223.9	119.49	ILMN_12965	C1ORF92	1

RNF157	5.694868	681.4727	119.66	ILMN_7339	RNF157	17
RNF186	1.663439	200.866	120.75	ILMN_20153	RNF186	1
SNAI3	12.11721	1471.526	121.44	ILMN_12922	SNAI3	16
SLC5A2	0.536188	66.03602	123.16	ILMN_17020	SLC5A2	16
ITGB6	2.639924	336.4649	127.45	ILMN_7787	ITGB6	2
SPATA18	2.009165	258.0338	128.43	ILMN_20280	SPATA18	4
SMOC2	7.819204	1005.54	128.6	ILMN_4650	SMOC2	6
SOD3	18.63371	2420.96	129.92	ILMN_20909	SOD3	4
SPINK4	5.80156	761.8686	131.32	ILMN_13769	SPINK4	9
BZRAP1	0.706909	94.59401	133.81	ILMN_11647	BZRAP1	17
PRMT7	2.581734	351.3502	136.09	ILMN_16564	PRMT7	16
LOC641522	0.792165	108.4631	136.92	ILMN_19721	LOC641522	17
DDC	71.19042	9764.058	137.15	ILMN_13520	DDC	7
FLJ10916	21.72226	2982.414	137.3	ILMN_28363	FLJ10916	2
TUBB2B	15.43952	2128.268	137.85	ILMN_11689	TUBB2B	6
CYP27A1	0.613948	84.94441	138.36	ILMN_2033	CYP27A1	2
GJB6	13.6998	1941.914	141.75	ILMN_14068	GJB6	13
AVP	4.734814	674.1046	142.37	ILMN_20332	AVP	
ODAM	21.09183	3008.952	142.66	ILMN_11065	ODAM	4
GPA33	9.277994	1326.763	143	ILMN_10719	GPA33	1
TTYH1	5.614807	807.2734	143.78	ILMN_23713	TTYH1	19
AQP12A	0.623433	90.71554	145.51	ILMN_7810	AQP12A	2
LOC390748	0.025041	3.707013	148.04	ILMN_180684	LOC390748	16
CEACAM6	85.67488	13170.25	153.72	ILMN_21866	CEACAM6	19
RPLP0	10.11024	1561.709	154.47	ILMN_22954	RPLP0	12
C19orf45	3.178663	504.4767	158.71	ILMN_16641	C19ORF45	19
TFF3	37.09296	5892.893	158.87	ILMN_6502	TFF3	21
SMOC1	4.021753	645.1943	160.43	ILMN_138307	SMOC1	14
GPT	1.480318	241.821	163.36	ILMN_11770	GPT	8
SPHK1	4.293174	701.7354	163.45	ILMN_19411	SPHK1	17
NUPR1	7.128844	1182.973	165.94	ILMN_171287	NUPR1	16
PNMA6A	4.575704	762.6991	166.68	ILMN_21456	PNMA6A	X
ENPP6	7.441016	1244.734	167.28	ILMN_4749	ENPP6	4
LY6H	2.075538	352.0347	169.61	ILMN_4079	LY6H	8
NOTUM	0.800666	136.9564	171.05	ILMN_169767	NOTUM	17
DUSP9	6.767826	1166.538	172.37	ILMN_15543	DUSP9	X
NAALAD2	0.023753	4.185102	176.19	ILMN_6830	NAALAD2	11
NEUROG2	18.48304	3262.948	176.54	ILMN_20030	NEUROG2	4
SPOCK1	14.93848	2649.591	177.37	ILMN_136935	SPOCK1	5
GSDMC	0.272046	51.24359	188.36	ILMN_15792	GSDMC	8
CYorf15B	0.872734	172.062	197.15	ILMN_182614	CYORF15B	Y
SH3BP1	5.350849	1063.05	198.67	ILMN_139251	SH3BP1	22
MT1A	10.75856	2145.278	199.4	ILMN_1797	MT1A	16
LGR5	51.44716	10318.77	200.57	ILMN_20455	LGR5	12
MANSC1	3.273968	664.0988	202.84	ILMN_14144	MANSC1	12
REG1B	18.36518	3808.565	207.38	ILMN_12437	REG1B	2
FLJ20433	0.750928	157.485	209.72	ILMN_5776	FLJ20433	9

ZNF702P	4.096591	881.843	215.26	ILMN_24422	ZNF702P	19
BAIAP3	0.153763	33.27725	216.42	ILMN_7057	BAIAP3	16
NUDT4	0.193313	42.73075	221.04	ILMN_17055	NUDT4	12
KIT	17.01032	3789.623	222.78	ILMN_8258	KIT	4
MTG1	5.391161	1207.195	223.92	ILMN_19544	MTG1	10
CD27	0.073517	16.92231	230.18	ILMN_4066	CD27	12
ZNF613	7.343125	1728.757	235.43	ILMN_27695	ZNF613	19
RHCG	0.179595	42.59236	237.16	ILMN_11870	RHCG	15
ANKRD47	2.309356	569.5677	246.63	ILMN_12421	ANKRD47	19
UPP1	5.708539	1483.585	259.89	ILMN_1606	UPP1	7
RNASE6	4.417512	1262.291	285.75	ILMN_14848	RNASE6	14
PRSS21	5.068359	1466.036	289.25	ILMN_4305	PRSS21	16
RCN3	2.725212	859.8717	315.52	ILMN_7768	RCN3	19
PODXL2	7.565604	2474.84	327.12	ILMN_26815	PODXL2	3
GCSH	5.070634	1688.25	332.95	ILMN_24106	GCSH	16
ALKBH7	5.188676	1742.14	335.76	ILMN_7229	ALKBH7	19
TSP50	4.28818	1475.938	344.19	ILMN_13617	TSP50	3
RASL12	8.065422	2827.973	350.63	ILMN_7345	RASL12	15
CNTFR	3.795155	1362.35	358.97	ILMN_12225	CNTFR	9
MT1G	18.65955	6979.014	374.02	ILMN_22286	MT1G	16
REG1A	22.33834	9130.261	408.73	ILMN_17810	REG1A	2
CNTNAP2	9.858815	4067.477	412.57	ILMN_137475	CNTNAP2	7
DFNB31	0.43402	180.1321	415.03	ILMN_8468	DFNB31	9
ME3	1.815202	758.1838	417.69	ILMN_24802	ME3	11
GPX2	12.51682	5446.975	435.17	ILMN_777	GPX2	14
CBLN1	1.522661	712.7545	468.1	ILMN_28176	CBLN1	16
CLCC1	1.886649	892.6639	473.15	ILMN_19789	CLCC1	1
AKAP10	0.503483	239.9242	476.53	ILMN_5307	AKAP10	17
DNAH7	2.792842	1346.627	482.17	ILMN_3644	DNAH7	2
CCND2	13.85193	6837.551	493.62	ILMN_166706	CCND2	12
C10orf99	0.917433	458.9367	500.24	ILMN_3635	C10ORF99	10
HMGCS2	9.122698	4634.474	508.02	ILMN_4183	HMGCS2	1
LEFTY1	6.490412	3753.013	578.24	ILMN_19746	LEFTY1	1
BEX5	1.686233	986.3549	584.95	ILMN_10339	BEX5	X
OXT	3.501514	2526.401	721.52	ILMN_23514	OXT	20
AGMAT	3.099308	2244.498	724.19	ILMN_24467	AGMAT	1
MRPL20	5.999738	4347.081	724.55	ILMN_859	MRPL20	1
NUDT11	3.507689	2601.835	741.75	ILMN_163686	NUDT11	X
HPGD	3.616816	2836.856	784.35	ILMN_174705	HPGD	4
HTRA1	6.280941	5862.015	933.3	ILMN_10981	HTRA1	10
FAM133A	1.950607	1820.614	933.36	ILMN_4401	FAM133A	X
AUTS2	2.625888	2504.43	953.75	ILMN_4348	AUTS2	7
TEX101	0.70335	684.5078	973.21	ILMN_27346	TEX101	19
A2ML1	8.591258	8506.108	990.09	ILMN_17375	A2ML1	12
ZDHHC19	1.75545	1756.125	1000.38	ILMN_27596	ZDHHC19	3
ZNF350	0.813382	840.1799	1032.95	ILMN_20125	ZNF350	19
PCDHB2	1.985324	2283.682	1150.28	ILMN_23902	PCDHB2	5

KLK4	0.469024	551.8898	1176.68	ILMN_137300	KLK4	19
SLCO2B1	0.900916	1092.106	1212.22	ILMN_1897	SLCO2B1	11
MON1B	2.390437	2902.657	1214.28	ILMN_27844	MON1B	16
SLC15A2	0.754435	1022.538	1355.37	ILMN_24842	SLC15A2	3
C19orf24	0.94995	1367.127	1439.16	ILMN_5267	C19ORF24	19
ACTG2	0.560897	846.8071	1509.74	ILMN_13364	ACTG2	2
VIL1	0.550488	905.1092	1644.19	ILMN_14423	VIL1	2
6-Mar	1.916738	3841.417	2004.14	ILMN_2629	6-Mar	5
METT11D1	0.00343	8.363373	2438.28	ILMN_15490	METT11D1	14
IRF8	0.256131	695.5698	2715.68	ILMN_20480	IRF8	16
CNIH2	0.038077	121.9326	3202.28	ILMN_19340	CNIH2	11
SOCS1	0.028821	129.607	4497.02	ILMN_3038	SOCS1	16
SLC25A2	0.008891	57.14733	6427.8	ILMN_13632	SLC25A2	5
TFF1	0.674112	4482.102	6648.9	ILMN_18189	TFF1	21
TM4SF5	0.344825	5138.946	14903.08	ILMN_28551	TM4SF5	17
